# Transcriptome Sequencing, and Rapid Development and Application of SNP Markers for the Legume Pod Borer *Maruca vitrata* (Lepidoptera: Crambidae)

**DOI:** 10.1371/journal.pone.0021388

**Published:** 2011-07-06

**Authors:** Venu M. Margam, Brad S. Coates, Darrell O. Bayles, Richard L. Hellmich, Tolulope Agunbiade, Manfredo J. Seufferheld, Weilin Sun, Jeremy A. Kroemer, Malick N. Ba, Clementine L. Binso-Dabire, Ibrahim Baoua, Mohammad F. Ishiyaku, Fernando G. Covas, Ramasamy Srinivasan, Joel Armstrong, Larry L. Murdock, Barry R. Pittendrigh

**Affiliations:** 1 Department of Entomology, Purdue University, West Lafayette, Indiana, United States of America; 2 United States Department of Agriculture – Agricultural Research Service, Corn Insect and Crop Genetics Research Unit, Genetics Laboratory, Iowa State University, Ames, Iowa, United States of America; 3 Department of Entomology, University of Illinois at Urbana-Champaign, Champaign, Illinois, United States of America; 4 Department of Crop Sciences, University of Illinois at Urbana-Champaign, Champaign, Illinois, United States of America; 5 Institut de l'Environnement et de Recherches Agricoles (INERA), Station de Kamboinsé, Ouagadougou, Burkina Faso; 6 Institut National de Recherche Agronomique du Niger, Maradi, Niger; 7 Department of Plant Science, Institute for Agricultural Research, Ahmadu Bello University, Zaria, Nigeria; 8 University of Puerto Rico, Mayaguez, Puerto Rico; 9 AVRDC-The World Vegetable Center, Tainan, Taiwan; 10 Ecosystem Sciences, The Commonwealth Scientific and Industrial Research Organization, Black Mountain, Australian Capital Territory, Australia; University of Wyoming, United States of America

## Abstract

The legume pod borer, *Maruca vitrata* (Lepidoptera: Crambidae), is an insect pest species of crops grown by subsistence farmers in tropical regions of Africa. We present the *de novo* assembly of 3729 contigs from 454- and Sanger-derived sequencing reads for midgut, salivary, and whole adult tissues of this non-model species. Functional annotation predicted that 1320 *M. vitrata* protein coding genes are present, of which 631 have orthologs within the *Bombyx mori* gene model. A homology-based analysis assigned *M. vitrata* genes into a group of paralogs, but these were subsequently partitioned into putative orthologs following phylogenetic analyses. Following sequence quality filtering, a total of 1542 putative single nucleotide polymorphisms (SNPs) were predicted within *M. vitrata* contig assemblies. Seventy one of 1078 designed molecular genetic markers were used to screen *M. vitrata* samples from five collection sites in West Africa. Population substructure may be present with significant implications in the insect resistance management recommendations pertaining to the release of biological control agents or transgenic cowpea that express *Bacillus thuringiensis* crystal toxins. Mutation data derived from transcriptome sequencing is an expeditious and economical source for genetic markers that allow evaluation of ecological differentiation.

## Introduction

The legume pod borer (LPB), *Maruca vitrata* (Lepidoptera: Crambidae) occurs throughout tropical and subtropical regions of the world. The larvae feed upon flowers and pods of more than 39 host plants mainly from the Family Fabaceae (leguminous plants) [1], [2], [3]. Host plants include the cultivated *Vigna unguiculata* subsp. *unguiculata* (cowpea), *Vigna unguiculata* subsp. *sesquipedalis* (yard-long bean), *V. radiata* (mung bean), *Glycine max* (soybean), *Pueraria phaseoloids* (puero), *Phaseolus lunatus* (lima bean), and *Cajanus cajan* (pigeonpea), as well as many wild species. Larvae feed on flowers, pods and peduncles [1]. This can lead to 20–80% yield losses in sub-Saharan Africa [2], [3], Southeast Asia [4], [5], [6], South Asia [7], [8], [9], and Central and South America. The control of *M. vitrata* damage to crops largely relies upon the timely application and availability of chemical insecticides [10], but their effectiveness is hindered by the tight larval webbing that reduces pesticide exposure [3]. Furthermore, the cost of insecticides is prohibitive to most subsistence farmers in developing nations [11], [12], [13]. The losses and subsequent control challenges posed by *M. vitrata* have led to the emergence of this species as a major threat to economic and humanitarian well-being in developing and under-developed nations. Over the past several decades significant advances have been made in the understanding of the life-history and distribution patterns of *M. vitrata*, but extensive genomic- and population-level data are still lacking.

Expressed sequence tags (ESTs) are collections of short sequence reads from cDNA templates and are representative of a suite of genes expressed in particular tissues, developmental stages, phenotypes, or treatment conditions [14], [15], [16]. EST datasets are often generated as entry points to genomic research that is aimed at solving agricultural problems [17], and such data have mainly been obtained for digestive tissues of crop pest species [18], [19], [20]. “Next Generation” sequencing (NGS) technologies are based on micro-scale pyrosequencing reactions [21], [22] carried out in parallel on a PicoTitrePlate™ [23] or flow cell [24]. These advances have resulted in the high throughput and low cost acquisition of EST reads from understudied species [25]. Functional annotation of EST-derived gene sequences is dependent upon their assignment to biochemical pathways using homology-based predictions with those of evolutionarily proximal model organisms. This approach has identified target molecules of biological insecticides in crop pest species [18], [20]. Members of the midgut-expressed carboxylesterase, glutathione S-transferase, and cytochrome P450 monoxygenase gene families are known to be involved in the detoxification of chemical insecticides (xenobiotics), and have been identified from lepidopteran EST resources [26]. Similarly, adaptation of insects to the host plant defenses often results from successful modulation of digestive enzymes and the ability to neutralize toxic defensive substances [27]. These features influence the ability of larvae to utilize a given plant as a food source. Additionally, cells in the midgut modulate peritrophic membrane permeability through the action of chitin synthases, chitinases, and associated matrix proteins, which have been implicated as factors affecting pathogen entry [28].

Since EST sequences are obtained at random from a library, homologous gene regions often are re-sampled and can be assembled into contiguous sequences (contigs) that are representative of genes [29] and alleles at specific loci [30]. Single nucleotide polymorphisms (SNPs) are point mutations that occur among alleles at a locus, and can be readily identified computationally from contig assemblies [31], [32], [33]. SNPs tend to be biallelic mutations and are represented at high density within genomes [34]. SNPs can be developed into molecular genetic markers that incur low cost and with minimal error during high throughput genotyping screens [35], [36]. Variation at putative SNP loci can rapidly be developed into molecular genetic markers, and applied to population genetic inference [37], and genome mapping [38]. SNP-based genetic markers show a low incidence of non-PCR amplifying “null” alleles and a high rate of successful marker development in Lepidoptera [18]. This contrasts with reports of microsatellite-based markers that tend to be problematical when used to genotype natural populations due to associations with repetitive DNA elements [39], [40], [41]. Microsatellites have also been shown to hitchhike within actively mobile transposons [42], [43], [44], or to be target sites for the integration of *Helitron*-like transposons [45]. This may justify avoiding the use of microsatellite markers for genotyping Lepidopteran species [43].

In the present paper we describe the 454-based pyrosequencing (i.e. Roche GS-FLX) of larval *M. vitrata* midgut and salivary gland transcripts. Functional annotation of these EST assemblies dramatically increases the genomic information for this non-model species, and has the potential to contribute to knowledge of larval gut physiology. Additionally, we demonstrate that EST assemblies are a source of mutation information from which high throughput SNP-based molecular genetic markers can readily be developed to assess population genetic structure and gene flow.

## Materials and Methods

### 454-based sequencing of larval midgut and salivary gland cDNA

Premolt 3^rd^, 4^th^ and 5^th^ instar *M. vitrata* larvae were collected in *RNAlater* (Ambion Inc.) in 2008 from: (i) *Sesbania cannabina* plants at the World Vegetable Center, Tainan County, southern Taiwan (ii) a laboratory colony raised at the Commonwealth Scientific and Industrial Research Organization, Canberra, Australia, and (iii) common bean *Phaseolous vulgaris* plants at Lares, Puerto Rico. Twenty larvae from each collection site were immersed in ice-cold phosphate buffered saline (PBS; 137 mM NaCl, 2.7 mM KCl, 4.3 mM Na2HPO4×7H2O, 1.4 mM KH2PO4, pH 7.4) (with 10% *RNAlater* solution) for 20 min, then salivary gland and midgut tissues were dissected, pooled, and placed in 500 uL ice-cold PBS. Dissected tissues were centrifuged at 800× g for 5 min and washed 3 times with ice-cold 1× PBS. Total RNA was extracted from these tissues using a TRIzol® Reagent (Invitrogen, Carlsbad, CA) according to the manufacturer's instructions. RNA was quantified on a NanoDrop 2000 (Thermo Scientific, Wilmington, DE). First-strand cDNA was synthesized from 1 µg of total RNA using a BD Smart PCR cDNA synthesis kit (BD Biosciences, San Jose, CA). The cDNA was then amplified using a BD mix (BD Biosciences, San Jose, CA) for 15 cycles following the manufacturer's protocol except that a modified CDS II/3′ primer 5′ – TAG AGG CCG AGG CGG CCG ACA TGT TTT GTT TTT TTT TCT TTT TTT TTT VN -3′ (IDT Inc.) was used to avoid long homopolymer repeats. Subsequent to first-strand synthesis, the cDNA was then amplified using PCR Advantage II polymerase (Clontech Inc.) with the following thermal cycling program: (i) 1 min at 95°C, (ii) 21 cycles of 95°C for 7 sec, and (iii) 21 cycles of 68°C for 6 min. A 2 µl aliquot of the PCR product was analyzed on a 1% agarose gel to determine the amplification efficiency. The PCR product was then subjected to SfiI digestion (10 units) for 2 h at 50°C to remove the concatemers formed by CDSIII/3′ and the SMART IV primers. A Qiaquick PCR purification kit (Qiagen, Valencia, CA) was used to remove the leftover primers and nucleotides from the amplified cDNA. The quality and quantity of the cDNA library was evaluated by both spectrophotometry and gel electrophoresis.

Sequencing and assembly: Amplified cDNA was submitted to the Keck Genomic Center (University of Illinois at Urbana Champaign) for library construction and sequencing. Two µg of amplified cDNA was used for library construction followed by pyro-sequencing on a Roche 454 GS-FLX (Roche, Basel, Switzerland) using established protocols [23]. The adaptor sequences were identified and the trim positions were changed in .sff files using the Cross-match (http://www.phrap.org), sff tools from Roche (https://www.rocheapplied-science.com) and custom-built Java scripts. Sequences shorter than 50 nucleotides or containing homopolymers (in which 60% over the entire length of the read is represented by one nucleotide) were not included for assembly. Raw sequence data were obtained from .sff files, and assembled into contigs using the Roche GS *De Novo* Assembler (*i.e.*, Newbler assembler) using default parameters (Seed step: 12, Seed length: 16, Min overlap length: 40, Min overlap identity: 90%, Alignment identity score: 2, and Alignment difference score: −3), and all of the non-redundant contigs were exported to a file in FASTA format.

### Sanger-based sequencing of whole adult cDNA

Two adult *M. vitrata* moths were collected in *RNAlater* in 2006 from a light trap at Samaru-Zaria, Nigeria. Total RNA from these adults was extracted using TRIzol® Reagent (Invitrogen) according to the manufacturer's instructions. RNA was quantified on a NanoDrop 2000 (Thermo Scientific). First strand cDNA synthesis was created using 1 µg of total RNA with a BD Smart PCR cDNA synthesis kit (BD Biosciences, San Jose, CA). The cDNA was then amplified by a BD mix for 15 cycles (BD Biosciences, San Jose, CA). The amplified cDNA was then polished with T4 DNA polymerase following a Proteinase K treatment. The resulting amplified cDNA was purified by a Qiaquick PCR Purification Kit (Qiagen, Valencia, CA). The amplified cDNA was normalized using a Trimmer Kit (Evrogen, Moscow, Russia). The normalization utilizes duplex-specific nuclease (DSN) digestion to remove abundant transcripts. Briefly, the cDNA was denatured and subsequently allowed to reassociate. The hybridization kinetics leads to equalization of the single- stranded cDNA fraction. Addition of DSN was used to remove double stranded cDNA formed by abundant transcripts. The normalized cDNA was then amplified using an Advantage 2 Polymerase with modified primers containing a *Not* I adapter site and then checked for quality. Following digestion by the *Not* I restriction enzyme and purification, the normalized cDNA was ligated into the *Not* I site in pBluescript II SK+ vector. The ligated cDNA was transformed to MAX Efficiency® DH5™ Competent Cells. The library titer was determined from an aliquot of transformation reaction without culture amplification. Sequencing reactions and fragment analyses on an ABI 3730XL sequencer was conducted by the Purdue University Genomics Core Facility, West Lafayette, IN. The Purdue Core Facility also performed vector sequence trimming, as well as PHRED quality parameter assessment and trimming which was q<30 (99% base call accuracy).

### Homology searches and functional gene annotation

The Newbler assembler was used to create a reference assembly of all EST data (combined set of 454- and Sanger-derived sequences), and this *M. vitrata* EST dataset was imported into the Blast2Go suite [46], [47]. Homology searches were carried out by query of the NCBI non-restricted protein database using the blastx algorithm ([48]; *E*-value≤1×10^−6^ and Hsp≥33 cutoffs). Prediction of putative genes, and annotation based on biological process (P), molecular functions (F), and cellular component (C) was accomplished by search of the Gene Ontology (GO) database (The Gene Ontology Consortium; http://www.geneontology.org/) using top-blastx descriptors output from Blast2go (*E*-value-Hit-Filter ≤10^−5^; Annotation Cutoff = 55; GO Weight = 5; Hsp-Hit Coverage Cutoff = 0), and subsequent retrieval of Enzyme Code (EC) designations directly from the GO website. Final sequence annotation steps used search of the inclusive InterPro databases (http://www.ebi.ac.uk/interpro/) using the InterProScan (IPS) tool [49], [50]. Biochemical pathway information was collected by downloading relevant maps from the Kyoto Encyclopedia of Genes and Genomes (KEGG; http://www.genome.jp/kegg/; [51]) using EC terms. Resulting biological process (P), molecular functions (F), and cellular component (C) at GO level 2 were reported, as were statistics on (i) distribution of sequence lengths, (ii) *E*-values, (iii) percent sequence similarity, and (iv) species from which blast hits were derived.

The derived (translated) protein products from gene model v. 2.3 of the model lepidopteran species *B. mori* in the file silkworm_glean_pep.fa.tar.gz from http://www.silkdb.org/silkdb/doc/download.html were downloaded. These sequences represented all predicted genes from the whole genome assembly of *B. mori*. This GLEAN-predicted protein sequence data was imported into a local database using the program BioEdit [52], queried with *M. vitrata* ESTs using the tblastx algorithm, and the results were filtered for *E*-values≤1×10^−40^. Due to the presence of paralogs or conserved protein domains within eukaryotic organisms, an individual *M. vitrata* EST resulted in >1 putative ortholog within the *B. mori* gene model. Protein coding frames were predicted for *M. vitrata* ESTs that were assigned the functional annotation as a putative membrane alanine aminopeptidase (APN) coding sequence using the program Virtual Ribosome (http://www.cbs.dtu.dk/services/VirtualRibosome/; [53]). FASTA formatted *M. vitrata* APNs were individually aligned against the nine predicted APNs within the *B. mori* GLEAN proteins (BGIBMGA001641, BGIBMGA001642, BGIBMGA008017, BGIBMGA008018, BGIBMGA008059 to BGIBMGA008063; Supplemental Data S3) using the ClustalW algorithm with default parameters (gap opening penalty 15, gap extension penalty 6.66, weight matrix IUB, and transition weight of 0.5). Derived full-length APN sequences were retrieved from the NCBI nr protein database (downloaded Oct 25, 2010 using keyword search “Lepidoptera” and “aminopeptidase N”), and aligned with the 9 GLEAN-predicted *B. mori* APNs with the ClustalW algorithm as described previously. APN alignments were imported into the MEGA 5.0 software package [54], and hierarchical relationships among APN paralogs (gene family members) were inferred using the Neighbor-Joining method [55] from the Poisson-corrected per site amino acid substitution model [56]. The NJ reconstruction method incorporated 1000 bootstrap pseudoreplicates to determine the number of possible trees supporting each node [57], and each reported as a strict consensus tree. Additionally, a Maximum Likelihood (ML) based model was also used to infer the phylogenetic relationship among lepidopteran aminopeptidases using MEGA 5.0 using the Jones-Taylor-Thornton (JTT) model of amino acids substitution from partial deleted site data (95% cutoff). The gamma distribution was estimated from empirical data, a heuristic tree search performed using the Close-Neighbor-Interchange (CNI) method, and branch support at each node estimated using 10000 bootstrap pseudoreplications and results reported as a strict consensus tree.

### Single nucleotide polymorphism (SNP) prediction and assay development

By design, the *de novo* assembler collapses the SNPs at a position into a single base call (the most commonly found base at that position, i.e. majority rule nucleotide calls). High confidence nucleotide differences, including but not limited to SNPs, were detected with the Roche GS Reference Mapper by mapping the sequencing reads to the corresponding *M. vitrata* reference assembly (section 2.2). The high confidence differences were saved to a flat file specifying full descriptions for the detected variants and treating each read as a separate read rather than grouping them into duplicates for variation detection. A custom script was used to parse a high confidence difference file that was output into both FASTA and tabular formats for all the SNPs that met quality criteria. These filtered SNPs were required to have at least 150 bp of flanking sequence on both sides of the SNP to support requirements for assay development. Molecular assays were developed to detect putative SNP variation identified within the combined *M. vitrata* EST assembly using a Sequenom MassARRAY® Designer software (Sequenom, San Diego, CA), and unmodified oligonucleotides were synthesized by Integrated DNA Technologies (Coralville, IA). Each SNP detection assay consists of an initial multiplex PCR step that amplifies genome regions containing mutations, followed by a single base extension reaction that incorporates mass-modified dideoxynucleotides complementary to the allele at each polymorphic locus using the iPLEX-Gold mastermix (Sequenom; [58]). Each multiplex PCR reaction typically can be designed to co-amplify up to 35 loci.

### SNP genotyping and population genetic analyses

**Figure 1:**
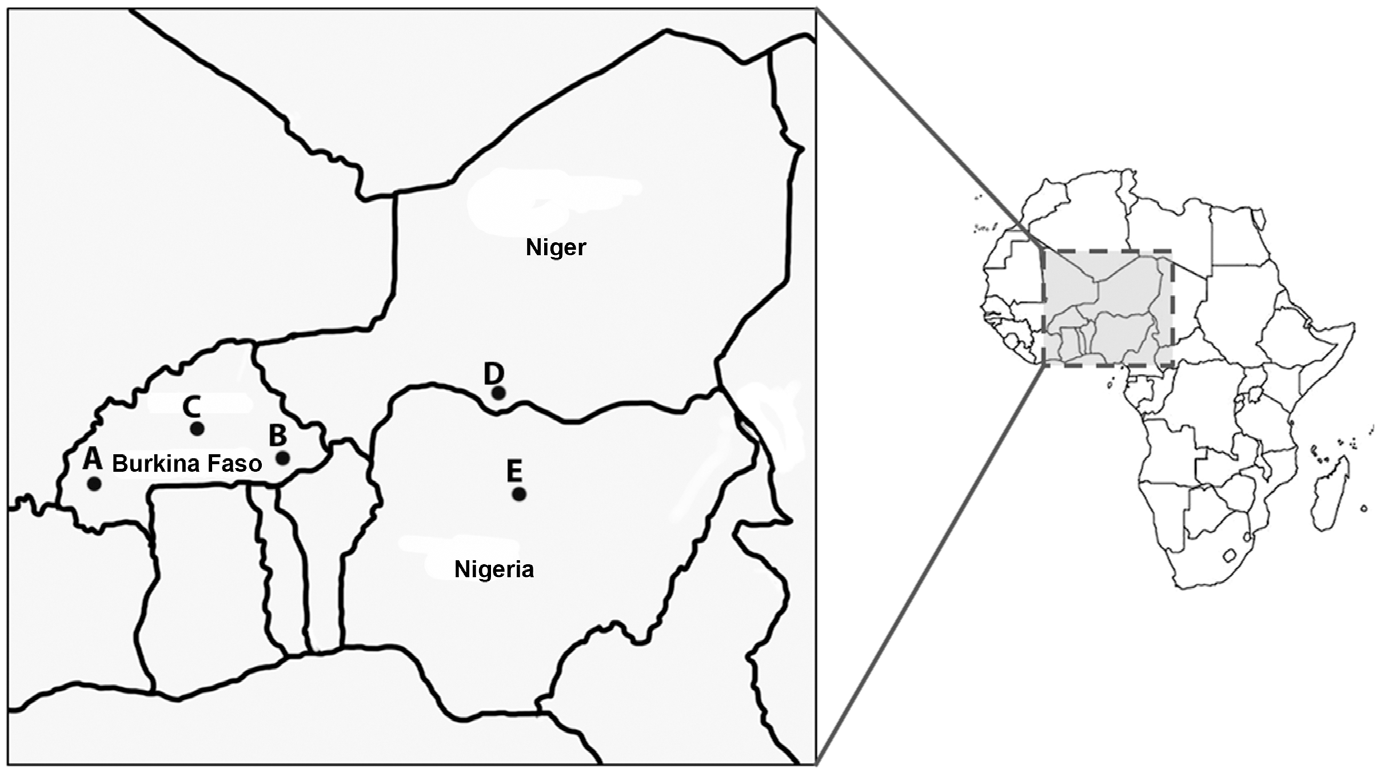
The location of collection sites for *Maruca vitrata* population samples.

*Maruca vitrata* samples were collected from sites in Burkina Faso, Niger, and Nigeria Africa from 2005 to 2007 (Fig. 1). DNA was extracted using Qiagen DNeasy Blood and Tissue Kit (Qiagen, Valencia, CA), and quantified on a Nanodrop 2000 (Thermo Scientific). Each SNP genotyping assay was performed on a Sequenom MassARRAY®, and consisted of an initial PCR step that amplified the genome region that contains an individual SNP, followed by a single base extension reaction that incorporated mass-modified dideoxynucleotides that are complementary to the polymorphic locus within each allele included in the iPLEX-Gold mastermix (Sequenom, San Diego, CA; Tang *et al.* 2004). Allele discrimination and subsequent genotyping was accomplished by Matrix-assisted laser desorption/ionization-time of flight (MALDI-TOF) mass spectrometry [59] on a Sequenom MassARRAY® located at the Iowa State Center for Plant Genomics (Ames, IA).

Exact tests for sample differentiation among pairwise *F*_ST_ estimates, and locus-by-locus *F*_ST_, *F*_IS_, and *F*_IT_ estimates for all populations were performed with Arlequin (v. 3.1; [60]; see references therein for all tests). Hierarchical population structure was assessed by an analysis of molecular variance (AMOVA) test using global genotypes and *F*_ST_ estimates as an average across all loci [61], [62], [63] for two assumed geographic partitions of *M. vitrata* collected from cowpea plants in eastern (Maradi, Niger, and Samaru, Zaria, Nigeria) and western regions (Station Agricole du Farakob, Fada N'gourma, and Kamboinsé sites in Burkina Faso; Fig. 1). Subsequent tests of hierarchical structure used all possible groupings of the population samples to investigate the range of *F*_ST_ values. An isolation-by-distance model was tested by the relationship between *F*_ST_/(1−*F*_ST_) employing SNP-based *F*_ST_ estimates and geographic distance between sample sites (log_10_ km) using the IBD web service v.3.15 (http://ibdws.sdsu.edu/~ibdws/; [64]), and significance was estimated by 1000 jackknifed permutation steps.

STRUCTURE 2.3.2.1 [65]) was used to estimate the number of distinct populations (*K*). Runs were carried out for each value of *K* from 1 to 10. Each run consisted of 9×10^6^ iterations, preceded by a burn-in of 10^5^ iterations that used an admixture model of individual ancestry. The median value of the estimated log probability of the data, conditional on K, (ln Pr(X|K)), was used to compute the posterior probability of *K*, Pr(*K*|X), assuming a uniform prior distribution for *K*. STRUCTURE 2.3.2.1 was also used to estimate the number of distinct populations (*K*; *K*_max_ = 10) given the *a priori* information of *K* = 5 (LOCPRIOR command; [66]), with all other parameters identical as in the 1st run except for the location information was included in a separate LocData column.

## Results

### *Maruca vitrata* expressed sequence tags and sequence assembly

The 454-based sequencing of the larval *M. vitrata* midgut and salivary gland library on a 1/2 gasketed plate resulted in a total of 88,841 reads, of which 12.8 Mb from 38,001 high quality reads were aligned using the Newbler Assembler. This assembly of digestive tissue-derived ESTs encompassed 3499 contigs with a mean of 452.9±279.9 bases, and contained a maximum contig length of 3299 bases (Table 1). Read data from the 454 sequencing run was submitted to the National Center for Biotechnology Information (NCBI) Short Read Archive (SRA), and can be retrieved as accession SRA020876.1. Additionally, assembled contig sequences are provided as online supporting information. A total of 2229 reads were obtained from Sanger sequencing of the *M. vitrata* whole adult cDNA library. These reads encompassed 1.3 Mb of sequencing data following trimming of vector and low-quality sequence. The processed read data was submitted to the NCBI EST database (dbEST) under accessions HS097571–HS099476. Assembly of the raw Sanger read data resulted in 2229 unique sequences that comprised of 1892 contigs and 337 singletons with a mean length of 561.4±300.5 bp (maximum 1812 bp; Table 1). The combined assembly of 454- and Sanger-based EST contig sequences (i.e. the Reference Assembly) resulted in 3729 contig sequences with a mean length of 459.6±287.3 bp (maximum 3299 bp), of which 430 sequences were not shared between libraries. This reference assembly was used for all subsequent functional gene annotation and single nucleotide polymorphism (SNP) predictions.

**Table 1:** Summary of *Maruca vitrata* expressed sequence tag (EST) data and assemblies.

	Sanger library	454 GS	Reference (combined)
Tissue	Whole adult	Salivary gland & midgut	-
Number of raw reads	2229	88,841	-
Number of raw bases	1,251,439	29,630,098	-
Number of reads assembled	2229	38,001	40,230
Number of bases assembled	1,251,439	12,767,552	14,018,991
Number of assembled contigs	1892	3499	3729
Mean contig length (bp)	561.4±300.5	452.9±279.9	459.6±287.3
Range of contig lengths (bp)	41–1812	92–3299	96–3299

### Homology, orthology, and functional gene annotation

**Figure 2:**
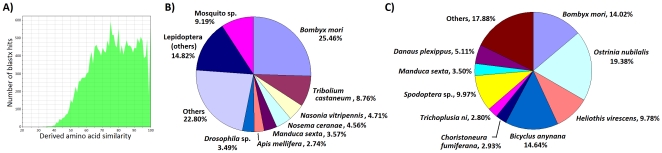
The distribution of sequence similarity values (A) and species distribution (B) among the top blastx “hits” resulting from queries of the NCBI non-restricted protein database with *M. vitrata* ESTs. The distribution of species among top blastn “hits” to the NCBI expressed sequence tag (EST) database, dbEST (C).

Search of the NCBI non-restricted protein database using 3729 unique *M. vitrata* sequences from the reference assembly (combined assembly of 454- and Sanger-derived sequence data) as queries using the blastx algorithm resulted in 1320 sequences (39.4%) with “hits” having an *E*-value≤9.0×10^−7^ and similarity ≥36.67% (mean = 75.8±13.7%; Fig. 2A). The greatest individual species representation among results of the blastx homology search was from the model insect species *B. mori* (25.46%) and *Tribolium castaneum* (8.66%; Fig. 2B). *Manduca sexta* (Lepidoptera: Sphingidae) comprised 3.57% of the hits, and other species of Lepidoptera accounted for 14.82%. Additionally, 4.7% of sequences showed homology to the endocellular microsporidial parasite *Nosema ceranae* (Fig. 2B), and following reference back to the source 454- and Sanger-based EST libraries indicated that microsporidial contamination came from the larval midgut and salivary gland sequences. A search of the NCBI dbEST resource using the tblastx algorithm resulted in predictions of homology for 2358 *M. vitrata* sequences (63.2%) to previously obtained ESTs. These “hits” to dbEST accessions in the “est_others” database showed *E*-values≤9.0×10^−7^ and sequence similarities ≥36.67% (mean = 75.8±13.7%), and a corresponding species distribution that was highly representative of lepidopteran ESTs sequences including the model species *B. mori* (Fig. 2C).

**Figure 3:**
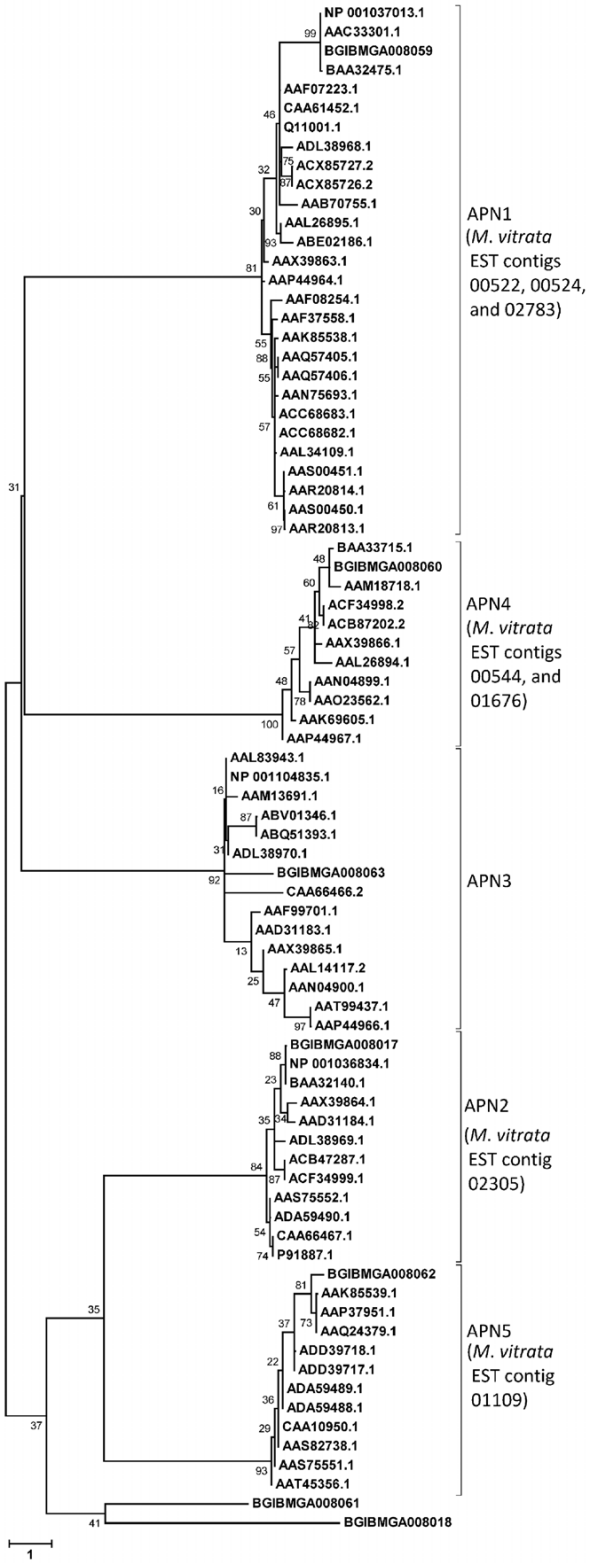
Phylogenetic relationship among aminopeptidase N (APN) proteins encoded by members of nine gene families in Lepidoptera. Clustering was used to assign gene orthology to derived APN peptide sequences sampled with the *Maruca vitrata* reference EST assembly. Predicted coding frame, derived peptide sequence, and multiple sequence alignments with the *Bombyx mori* GLEAN proteins sequences are shown in Supplementary Data S3).

Results from querying a local database comprised of the GLEAN predicted protein sequences from the *B. mori* gene model v. 2.3 using the tblastx algorithm provided putative ortholog assignments for 631 *M. vitrata* EST-derived contig sequences (*E*-values≤3.0×10^−41^; similarities ≥26.1%; Supplementary Data S1), which also resulted in homology matches of *M. vitrata* ESTs to ≥1 gene sequence in the *B. mori* gene (2.04±3.59 ortholog matches per *M. vitrata* EST). The blastx results indicated for the ortholog matches were confounded by the presence of closely related gene family members, such that definitive orthologous gene relationships could not depend solely upon homology estimates. Protein coding frames were predicted for *M. vitrata* ESTs with functional annotation of putative membrane alanine aminopeptidase (APN) encoding genes using the program Virtual Ribosome (http://www.cbs.dtu.dk/services/VirtualRibosome/; [53]; Supplementary Data S2), which were subsequently aligned against 9 predicted APNs within the *B. mori* GLEAN proteins (BGIBMGA001641, BGIBMGA001642, BGIBMGA008017, BGIBMGA008018, BGIBMGA008059 to BGIBMGA008063; Supplemental Data S3). Phylogenetic reconstruction of the aminopeptidase N gene family from Lepidoptera using NJ and Maximum Likelihood methods both resulted in 9 distinct groups (APN1 to APN9; Fig. 3) and showed congruence in tree topology between methods. The APN clades defined solely by BGIBMGA001641, BGIBMGA001642, BGIBMGA008018, and BGIBMGA008061 were monophyletic, whereas BGIBMGA008059 (APN1), BGIBMGA008017 (APN2), BGIBMGA008063 (APN3), BGIBMGA008060 (APN4), and BGIBMGA008062 (APN5) encompassed all midgut-expressed orthologs previously identified from other species of Lepidoptera.

**Figure 4:**
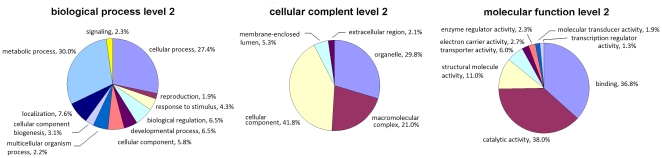
Gene ontologies ascribed to *Maruca vitrata* EST top blast hit terms by the Gene Ontology (GO) database. Assignments for biological process (P), cellular complement (C), and molecular function (F) are shown at GO level 2 (Note: a given EST sequence may be assigned >1 GO term).

A total of 3676 gene ontology (GO) annotations were obtained for *M. vitrata* EST sequences (5.50±1.83 GO annotations per sequence). At level 2, the distribution of GO terms among biological process (P), cellular component (C), and molecular function (F) showed metabolic process, cellular component, and catalytic activity respectively to be at the highest frequency (Fig. 4A to 4C). A total of 543 unique InterPro annotations were made for *M. vitrata* EST data, and the 15 most frequent InterPro entries encountered are listed in Table 2. Roles in catalytic function among predicted genes within the annotated ESTs at GO level F (molecular function) and metabolic process (GO level P; biological process) was corroborated by the observation of 36 serine protease (IPR009003, IPR001254, and IPR018114), esterase (IPR002018), and hydrolase activities (IPR017853 and IPR013781; Table 2). GO terms for cellular component (C) show that structural cellular component proteins are highly represented, including insect cuticle protein coding genes, are highly represented (InterPro accession IPR000618; Table 1). Full annotation information for *M. vitrata* ESTs from Sanger reads and the reference assembly are available at LepDB.org (Coates *et al.* unpublished).

**Table 2:** The 15 most encountered InterPro accessions present with annotated *Maruca vitrata* EST sequences, and corresponding *B. mori* gene models with matching InterPro annotation.

InterPro Entry	No.	IPR description(s)
IPR013032	9	EGF-like region, conserved site
IPR007087	9	Zinc finger (Znf) domain, C2H2-type
IPR009003	8	Peptidase, trypsin-like serine and cysteine
IPR001254	8	Peptidase_S1/S6, chymotrypsin-like/Hap
IPR016040	7	NAD- and NADP-binding domain
IPR018114	5	Peptidase S1/S6, chymotrypsin/Hap active site
IPR017853	5	Glycoside hydrolase, catalytic core
IPR013781	5	Glycoside hydrolase, subgroup, catalytic core
IPR002198	5	Short-chain dehydrogenase/reductase SDR
IPR002018	5	Carboxyesterase, type B
IPR000215	5	Protease inhibitor I4, serpin
IPR015590	4	Aldehyde dehydrogenase domain
IPR012335	4	Thioredoxin fold
IPR009072	4	Histone-fold
IPR008978	4	Heat shock protein 20-like chaperone

### Single nucleotide polymorphism (SNP) prediction and assay development

The Newbler Assembler generated a *de novo* reference assembly of *O. nubilalis* midgut and antennal EST reads that contained 3729 contigs (i.e., combined assembly of 454- and Sanger-based EST contig sequences; mean length 459.6±287.3 bp). Polymorphism among constituent reads within each contig was used to predict 2620 putative SNPs using the Newbler Mapping suite. Quality score criteria and proximity to 5′ and 3′ ends of respective contigs resulted in the removal of 1542 of 2620 putative SNPs (58.9%) from the pool of candidate loci, and the 1078 remaining loci were used for SNP marker development. Single base extension-based PCR assays were designed using the Sequenom MassARRAY Designer Software to detect 139 of the remaining 1078 predicted SNP loci (12.9%). The 139 SNP assays were also designed to co-amplify within 4 separate PCR multiplex reactions (multiplexes W1 to W4; 31.25±5.68 markers per multiplex).

Within the present study, 70 SNP loci from multiplex PCR reactions W1 and W2 (markers MvSMA-0001 to −0070; Supplementary Data S4) were used to genotype 375 of 383 DNA samples (97.9%) extracted from *M. vitrata* that were collected from 5 locations in Africa (**Fig. 1**). Genotyping results showed that 29 of 70 markers (41.4%) failed to produce a signal in >75% of individuals from population samples when separated on the Sequenom MassARRAY®, and 6 of 70 markers showed no polymorphism among samples (8.6% = SNP false discovery rate). In total, 35 of 70 SNP loci (50.0%) resulted in polymorphic genetic markers that were scored from Sequenom MassARRAY® output and further analyzed for population genetic parameters.

### SNP genotyping and population genetic analyses

In total, output from the Sequenom MassARRAY® contained 20,836 of 22,302 possible genotypes (6.6% failure rate). Permutation tests show that 17 of the 35 SNP marker loci that were polymorphic within the African samples (48.6%), and also showed heterozygosity levels that do not significantly deviate from that expected under Hardy-Weinberg Equilibrium (HWE) in any population (*P*≥0.05; Supplementary Data S5). A total of 11 loci, MARVI- Contig1478_266, -Contig1663_832, -Contig172_349, -Contig172_430, -Contig180_816, -Contig315_310, -Contig355_182, -Contig702_243, -Contig896_1927, -Contig98_663, and -Contig_1692_590, were polymorphic in ≥5 of 6 populations wherein a total of 22 alleles were scored among 166 individuals. SNP minor allele frequencies (MAFs) ranged from 0.294 (marker MARVI-Contig180_816) to 0.053 (marker MARVI-Contig315_310) across all populations and all loci (mean = 0.141±0.092; Table 3). Inbreeding coefficient (*F*_IS_) and *F*_IT_ estimates across all populations and all loci ranged from −0.007 to 0.085 and −0.034 to 0.086, respectively. The global estimates of *F*_IS_ and *F*_IT_ averaged across loci also were not significant (*P*≥0.05; Table 3).

**Table 3:** Population statistics for African *Maruca vitrata* collection sites (Site ID correspond to those given in Fig. 1).

Contig		1478	1663	0172	0172	0180	0315	0355	0702	0896	0098	1692
Position		266	832	349	430	816	310	182	243	1927	663	590
MAF	SiteA	0.011	0.138	0.108	0.044	0.178	0.000	0.078	0.110	0.092	0.000	0.176
	SiteB	0.023	0.083	0.238	0.239	0.278	0.000	0.133	0.000	0.114	0.048	0.105
	SiteC	0.068	0.182	0.214	0.000	0.308	0.079	0.000	0.000	0.143	0.000	0.059
	SiteD	0.159	0.250	0.068	0.068	0.423	0.083	0.033	0.067	0.158	0.048	0.344
	SiteE	0.063	0.267	0.197	0.027	0.265	0.053	0.125	0.000	0.206	0.026	0.318
	μ	0.065	0.145	0.165	0.076	0.290	0.043	0.074	0.035	0.143	0.024	0.200
	σ	0.058	0.077	0.073	0.095	0.089	0.041	0.058	0.051	0.044	0.024	0.127
	H_E_	0.045	0.157	0.372	0.048	0.413	0.089	0.239	0.241	0.000	0.093	0.193
	H_O_	0.045	0.167	0.286	0.048	0.556	0.091	0.133	0.273	0.000	0.000	0.105
	*F*_ST_	0.026	0.164	−0.009	−0.005	−0.007	0.001	−0.017	−0.019	0.018	−0.024	0.107
	*F*_IS_	0.298	0.492	0.130	−0.031	−0.277	−0.050	0.555	0.660	0.075	0.489	−0.009
	*F*_IT_	0.329	0.631	0.126	−0.034	−0.268	−0.040	0.553	0.664	0.102	0.794	0.100

The minor SNP allele frequency (MAF) is given by locus within and across all population samples. The overall North American population mean (μ) and variance (σ) as well as observed (H_O_) and expected heterozygosity (H_E_) are given for each locus. Lastly, locus specific *F*_ST_, *F*_IS_, and *F*_IT_ estimates among populations are given.

**Figure 5:**
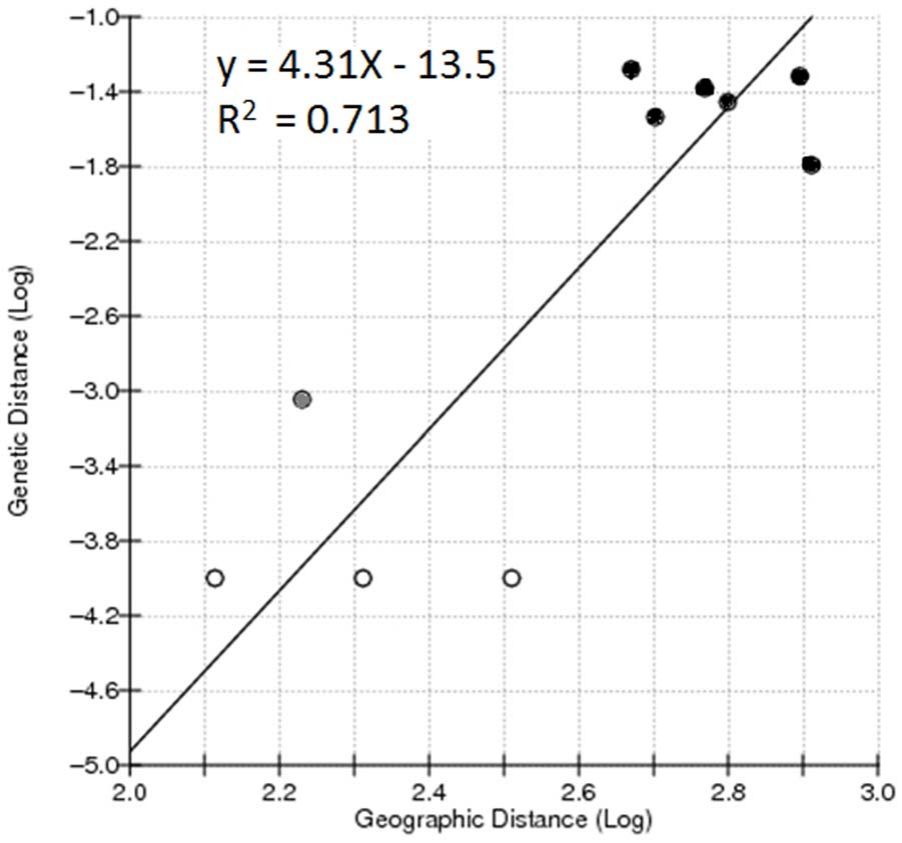
Genetic isolation-by-distance analysis by regression of Log_10_(*F*_ST_) and Log_10_(geographic distance, km). Comparisons among Burkina Faso (Sites A, B, and C) are represented as open dots (○), between Burkina Faso and Niger (Site D) or Nigeria (Site E) closed dots (•), and are between Niger and Nigeria (grey dot).

The locus-by-locus *F*_ST_ estimates for the 11 *M. vitrata* SNP markers ranged from −0.024 to 0.164 among subpopulations and showed a global estimate of 0.021±0.060 (Table 3). The pairwise *F*_ST_ estimates calculated between sample sites ranged from −0.0192 to 0.0524, and were significant only for four comparisons: 1) between Station Agricole du Farakoba, Burkina Faso and Maradi, Niger; 2) between Station Agricole du Farakoba, Burkina Faso and Samaru, Zaria, Nigeria; 3) Fada N'gourma, Burkina Faso and Maradi, Niger; and 4) Kamboninse, Burkina Faso and Samaru, Zaria, Nigeria (*P*≤0.0451). No comparisons surpassed a Bonferonni adjusted significance threshold of 0.005 (α = 0.05÷10; Table 4). Regression of Log_10_(*F*_ST_) and Log_10_(geographic distance, km) values showed a correlation between the two parameters (r = 0.740; Z = 129.5; Fig. 5), but Mantel Tests for significance did not reject the null hypothesis (*P*-value = 0.1000). Exact tests of sample differentiation based on genotype frequencies from a non-differentiation exact *P*-value = 0.12276 indicated that no significant pairwise comparisons were present among the five *M. vitrata* sample sites in Africa (Fig. 1; *P*-values≥0.1461; data not shown).

**Table 4:** Pairwise *F*_ST_ estimates (below diagonal) and corresponding *P*-values (above diagonal).

Site ID	SiteA	SiteB	SiteC	SiteD	SiteE
SiteA	–	0.6577	0.6036	0.0090*	0.0451*
SiteB	−0.0102	–	0.9369	0.0180*	0.0812
SiteC	−0.0074	−0.0192	–	0.1081	0.0180*
SiteD	0.0482	0.0524	0.0416	–	0.3694
SiteE	0.0161	0.0291	0.0350	0.0009	–

Comparisons surpassing a significance threshold of α = 0.05 (*) and a Bonferroni adjusted α = 0.005 (**) are indicated. Site ID correspond to those given in Fig. 1).

Tests of hierarchical population structure conducted using AMOVA and *F*-statistics. An assumed structure comprised on an eastern (Maradi, Niger, and Samaru, Zaria, Nigeria) and a western group (Station Agricole du Farakoba, Fada N'gourma, and Kamboinse sites in Burkina Faso; Fig. 1) resulted in an estimated *F*_ST_ = 0.040 (*P* = 0.112) and 4.0% of total genetic variation accounted for by differences between groups when calculated using the percentage of pairwise differences between genotypes. The same method of estimation also indicated an *F*_IS_ = −0.018 (*P*<0.651) and *F*_IT_ = 0.017 (*P*<0.576). Analogously, when calculated as a weighted average across all 11 loci, the *M. vitrata* from the eastern and western sample sites showed an *F*_ST_ estimate of 0.046 (*P*<0.002) and 4.63% of total genetic variation due to differences among groups, and the corresponding values of *F*_IS_ = 0.110 (*P*<0.049) and *F*_IT_ = 0.172 (*P*<0.021) and 82.8% of total genetic variation within populations was estimated to be partitioned within individuals. No other hierarchical tests, using random grouping of genotypes from the five *M. vitrata* sample sites, resulted in *F*_ST_ estimates that reached a significance threshold ≥0.05.

**Figure 6:**
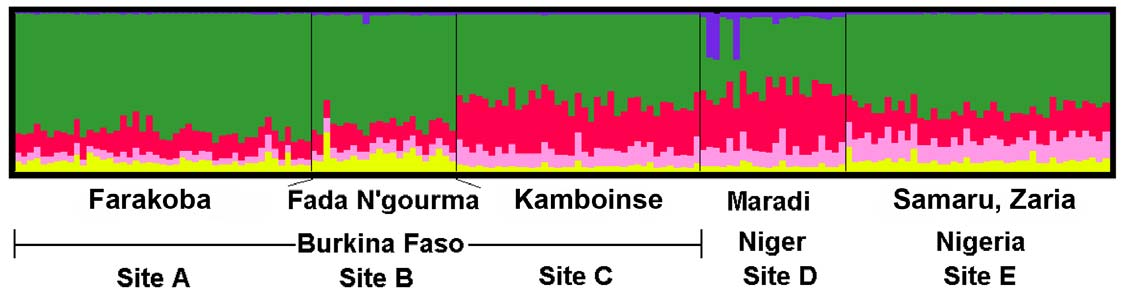
The estimated ancestry coefficients (*Q*) for *Maruca vitrata* individuals from STRUCTURE output generated by the LOCPRIOR command. The ancestry of individual SNP genotypes from sampled subpopulations. Each of the *M. vitrata* individuals are represented as vertical lines, and are composed of the proportion of the *K* ^th^ segment that representative of the ancestry in the respective genotype.

Analyses using the program STRUCTURE revealed that population subdivision may exist, where the posterior probabilities for a value of *K*>1 approached a maximum of 0.3679 at *K* = 3. The lnPr(X|K) decreased drastically for *K* = 2 (9.2×10^−9^) and *K* = 4 (4.8×10^−23^). The partitioning of ancestry within individual genotypes indicated that 82.8% was contained within two clusters (cluster 1 = 21.3±9.1%; cluster 2 = 61.5±11.8%; Fig. 6).

## Discussion

### *Maruca vitrata* expressed sequence tags and sequence assembly

The sequence libraries from midgut, salivary, and whole adult tissues are the first EST resources reported for *M. vitrata*, and one of a few dozen currently available from the NCBI dbEST database for species of Lepidoptera. Vera *et al.* (2008) [25] were the first to report the use of NGS technology for the rapid acquisition of transcriptome sequence data from a non-model insect species that was subsequently analyzed via functional gene annotation, gene orthology estimation with a model species, and prediction of putative mutations. 454-based transcriptome sequencing for non-model organisms has since become more commonplace [26], but it is still fraught with errors derived from sensitivity to homopolymer stretches and from the overestimation of total gene number caused by fragmented assemblies [23], [25], [67]. These phenomena were also observed in our *M. vitrata* EST assemblies, where the mean contig length for the 454-based EST assembly was lower compared to that resulting from Sanger-based read data (albeit, direct comparisons are difficult since assemblies were derived from different sets of genes in different tissues). Although independent reads and mean contig lengths are shorter for 454-based assemblies, the maximum length of contigs tends to be higher than that observed for Sanger sequencing [26]. This may result from assemblies that use relaxed parameters for joining overlapping sequences wherein chimeras may be produced that are comprised of closely related paralogous [68]. Conversely, highly stringent parameters may place alleles at a locus into unique contigs. This latter strategy likely is a cause for the overestimation of total gene counts and fragmented assemblies due to an effective reduction in sequence coverage per gene, and is not ideal for SNP discovery.

### Homology, orthology, and functional gene annotation

The prediction of gene-coding sequence within EST datasets from non-model species is typically achieved via functional annotation, a process that relies upon the homology-based identification of related genes within model organisms [46], [47]. Difficulties arise when a significant proportion of genes cannot be assigned functions or are given vague functions [69], which is common for other non-model species [18], [19], [26] and for our *M. vitrata* EST dataset. This may prove especially difficult with ESTs since individual sequences rarely encompass the entire gene, have nebulous reading frames, or are comprised mostly of non-coding regions. Furthermore, non-model species often have unique gene lineages that are not represented within the molecular databases typically used for comparative annotation. This last issue was addressed by Coates *et al* (2008) [18], where authors predicted open reading frames within un-annotated EST sequences from *Ostrinia nubilalis*. Although these *O. nubilalis* hypothetical proteins had no homologs within the NCBI protein database, similar proteins of unknown function were predicted within ESTs from related non-model species. These findings indicate that current annotation protocols are not adequately built to describe the utility of EST datasets. Given the accelerating pace of NGS data production and sequence assembly submissions to molecular databases from lesser-studied groups, researchers should focus on developing annotation resources specific to different branches of evolution.

### Single nucleotide polymorphism (SNP) prediction and assay development

Next generation sequencing (NGS) technology offers a means for researchers to rapidly acquire genomic data that have a bearing on biological questions, accelerate genetic discoveries, and develop genetic tools in non-model species. Expressed sequence tags are a rich source of mutation data from which SNPs can readily be identified [30], [70]. These SNPS can be validated by several novel genotyping technologies [36], [71]. The usefulness of a given EST resource for identifying SNP loci *in silico* depends upon the assembly depth and the diversity of tissues and individuals sampled within the library [72], [73]. Additionally, SNPs can be predicted from pools of closely related species, from which shared ancestral and uniquely derived mutations within lineages can be identified [74]. Earlier studies have described the development of SNP markers from NGS data and their subsequent application for individual genotyping [75], [76], [77], but few have been described for species of Lepidoptera [18].

Molecular genetic markers based upon microsatellite variation among alleles have been used to estimate population genetic parameters, Notable exceptions include crustaceans [78], mollusks [79] and lepidopterans [80]. Microsatellite loci in lepidopteran genomes have been described as either hitchhiking within transposons [42], [44], as sites for the integration of transposable elements [45], or being the consequence of target site duplication by retrotransposons [80]. A number of variables can prevent the validation of putative SNPs. When a SNP is predicted within an EST assembly from species that lacks a whole genome assembly, unknown intron positions and sequence similarity among paralogous genes may lead to failure of PCR amplification and a lack of locus specificity. Despite these inherent difficulties the rate of validation at putative SNP loci ranges from 29 to 50% [80], [81], where the contributing factor to marker failure is in the generation of consistent nucleotide calls and presence of monomorphic loci [82]. The 50% rate of validation for putative *M. vitrata* SNPs was typical of that observed for other non-model species, and provided a pool of marker genetic markers that were subsequently used to genotype individuals from 5 collections sites in West Africa.

### SNP genotyping and population genetic analyses

SNPs have been used to generate genetic linkage maps of the model lepidopteran species, *Bombyx mori* [82], and the butterfly *Bicyclus anynana* [83], but the utility of SNPs for population genetic inference within a species of Lepidoptera has not yet been proven [18]. Lack of genetic and genomic resources for insect pest species has hindered efforts to control pests and determine their movement within populations. These are processes essential for the development of resistance management strategies. Genetic markers have been developed from putative SNPs identified from sequence datasets of the crop pest species *Aphis glycines* [84]. Similar markers were used to genotype *O. nubilalis* populations where additional criteria, including adherence to Hardy-Weinberg Equilibrium (HWE), were applied during validation [18]. In total, 24 of 41 *M. vitrata* SNP markers (58.5%) deviated significantly from HWE expectations; with an excess of observed homozygosity being a potential source of the skew. The proportion of *M. vitrata* SNP markers that deviated significantly from HWE was higher than that reported for *O. nubilalis* [18], and may be caused by inbreeding, assortative mating, the Wahlund effect, or selection. A reduction in overall heterozygosity due to the Wahlund effect may be a likely cause since *M. vitrata* is a migratory species that moves northward into sub-Saharan regions from coastal regions during the rainy season, with no evidence of reverse migration. This seasonal movement may result in the temporal admixture, and be a potential explanation for the observed reduction in estimated heterozygosity frequency of *M. vitrata* sampled from West Africa, but additional and more detailed studies are required in order to draw any additional conclusions.

*Maruca vitrata* is the major insect pest of cowpea in West Africa [85]. In this region insecticides and sprayers are often prohibitively expensive or otherwise unavailable to low-income farmers in West Africa [86]. The analyses of *M. vitrata* SNP markers suggested that genetic structuring may occur within West Africa. Specifically, the pairwise *F*_ST_ estimates show that significant levels of differentiation were present between eastern and western sample sites, whereas all other comparisons indicated a lack of genetic divergence. Additionally, a positive correlation between pairwise *F*_ST_ estimates (used as a genetic distance estimate) and linear geographic distances suggests that the *M. vitrata* population may show genetic isolation by distance. Although this distance model was not fully supported by the estimation of co-ancestry contained within individual genotypes, STRUCTURE results did indicate that two divergent genotypes exist within West Africa. Understanding *M. vitrata* population dynamics and migratory patterns has important implications for resistance management plans for *Bt* cowpea and for the deployment of biocontrol agents in endemic zones. These tactics may help increase local food production in West Africa, reduce malnutrition, and stabilize grain commodity prices. Gene flow barriers and significant levels of genetic differentiation within a target pest populations influence that scale of insect resistance management practices typically implemented to maintain susceptibility and preserve the efficacy of pest management tools such as Bt crops and lay the foundation for cost effective bio-control agent release programs. For example, as these data are in keeping with an endemic zone to migratory zone hypothesis, the deployment of biocontrol agents (for classical biological control) would be most logical in the endemic zone directly south of migratory regions where *M. vitrata* is a significant pest during the cowpea growing season. Moreover, these results demonstrate that putative SNP data predicted within transcriptome sequence assemblies can be used to develop molecular genetic markers for the evaluation of real-world populations and the collection of data relevant to insect control.

## Supporting Information

Data S1 Prediction of putative gene orthology between the gene sequences within the *Maruca vitrata* combined EST assembly (i.e. reference assembly) and translated products from the GLEAN predicted *Bombyx mori* gene model v. 2.3.
(DOC)

Data S2 Derived amino acid sequence of Maruca vitrata EST contigs assigned putative functional annotation as alanine aminopeptidase (APN) encoding gene sequences.
(DOC)

Data S3 CLUSTAL 2.0.12 multiple sequence alignments of derived Maruca vitrata alanine aminopeptidase (APN) amino acid sequences with nine Bombyx mori APNs from the GLEAN-predicted gene model v. 2.3.
(DOC)

Data S4 Oligonucleotide primers used in Maruca vitrata PCR multiplex W1.
(DOC)

Data S5 Exact test to determine the adherence of Maruca vitrata single nucleotide polymorphism (SNP) markers to Hardy-Weinberg Equilibrium (HWE) proportions within natural populations collected from Africa. Markov chain (for all loci) used a forecasted chain length of 1,000,000 and dememorization steps of 100,000. P-values that surpassed a significance threshold of α = 0.05 are indicated with an asterisk (*).
(DOC)
