# Supplementary material for: Transcriptome Sequencing, and Rapid Development and Application of SNP Markers for the Legume Pod Borer Maruca vitrata (Lepidoptera: Crambidae)
Source: PLoS One. 2011 Jul 6;6(7):e21388. doi: 10.1371/journal.pone.0021388 (PMC3130784; doi:10.1371/journal.pone.0021388)
Supplement: Data S1 — Prediction of putative gene orthology between the gene sequences within the Maruca vitrata combined EST assembly (i.e. reference assembly) and translated products from the GLEAN predicted Bombyx mori gene model v. 2.3. (DOC) [file pone.0021388.s001.doc]

**Supplementary Data S1:** Prediction of putative gene orthology between the gene sequences within the *Maruca vitrata* combined EST assembly (i.e. reference assembly) and translated products from the GLEAN predicted *Bombyx mori* gene model v. 2.3.

| *Maruca vitrata*  EST contig | *Bombyx mori* gene  model | InterProScan annotation | Gene description |
| --- | --- | --- | --- |
| contig00003 | BGIBMGA013930 | IPR000639 | Epoxide |
| contig00003 | BGIBMGA013929 | IPR000639 | Epoxide |
| contig00003 | BGIBMGA013994 | IPR012336 | Thioredoxin-like |
| contig00003 | BGIBMGA009211 | IPR000639 | Epoxide |
| contig00003 | BGIBMGA013793 | IPR010497 | Epoxide |
| contig00003 | BGIBMGA011468 | IPR000639 | Epoxide |
| contig00006 | BGIBMGA009863 | IPR006646 | KOW |
| contig00012 | BGIBMGA005641 | IPR000719 | Protein |
| contig00012 | BGIBMGA007238 | IPR000719 | Protein |
| contig00012 | BGIBMGA007646 | IPR011009 | Protein |
| contig00012 | BGIBMGA001458 | IPR002290 | Serine/threonine |
| contig00019 | BGIBMGA012981 | IPR009038 | GOLD |
| contig00021 | BGIBMGA007258 | IPR002198 | Short-chain |
| contig00021 | BGIBMGA007259 | IPR002347 | Glucose/ribitol |
| contig00027 | BGIBMGA008280 | IPR001254 | Peptidase |
| contig00027 | BGIBMGA008281 | IPR001254 | Peptidase |
| contig00027 | BGIBMGA008279 | IPR001254 | Peptidase |
| contig00027 | BGIBMGA008278 | IPR001254 | Peptidase |
| contig00027 | BGIBMGA008242 | IPR001254 | Peptidase |
| contig00034 | BGIBMGA005953 | IPR001251 | Cellular |
| contig00034 | BGIBMGA005955 | IPR001251 | Cellular |
| contig00034 | BGIBMGA005954 | IPR001251 | Cellular |
| contig00034 | BGIBMGA013015 | IPR001251 | Cellular |
| contig00034 | BGIBMGA013016 | IPR001251 | Cellular |
| contig00037 | BGIBMGA008501 | IPR006186 | Serine/threonine-specific |
| contig00052 | BGIBMGA010130 | IPR001464 | Annexin, |
| contig00054 | BGIBMGA002670 | IPR000734 | Lipase, |
| contig00054 | BGIBMGA002669 | IPR000734 | Lipase, |
| contig00055 | BGIBMGA002669 | IPR000734 | Lipase, |
| contig00055 | BGIBMGA002670 | IPR000734 | Lipase, |
| contig00057 | BGIBMGA012549 | IPR000793 | ATPase, |
| contig00057 | BGIBMGA012555 | IPR004100 | ATPase, |
| contig00057 | BGIBMGA003901 | IPR000194 | ATPase, |
| contig00059 | BGIBMGA009208 | IPR002672 | Ribosomal |
| contig00060 | BGIBMGA005793 | IPR003204 | Cytochrome |
| contig00067 | BGIBMGA007941 | IPR000210 | BTB, |
| contig00068 | BGIBMGA003340 | IPR008934 | Acid |
| contig00075 | BGIBMGA008192 | IPR008594 | Scavenger |
| contig00083 | BGIBMGA003177 | IPR001680 | WD-40 |
| contig00087 | BGIBMGA001252 | undefined |  |
| contig00095 | BGIBMGA007948 | IPR002048 | Calcium-binding |
| contig00103 | BGIBMGA001509 | IPR001680 | WD-40 |
| contig00104 | BGIBMGA005131 | IPR000010 | Proteinase |
| contig00105 | BGIBMGA011803 | IPR000608 | Ubiquitin-conjugating |
| contig00106 | BGIBMGA003160 | IPR007087 | Zinc |
| contig00117 | BGIBMGA007690 | IPR004843 | Metallophosphoesterase, |
| contig00119 | BGIBMGA013536 | IPR001623 | Heat |
| contig00123 | BGIBMGA007559 | IPR006696 | Protein |
| contig00126 | BGIBMGA011168 | IPR000043 | S-adenosyl-L-homocysteine |
| contig00126 | BGIBMGA009808 | IPR000043 | S-adenosyl-L-homocysteine |
| contig00129 | BGIBMGA000681 | IPR004145 | Protein |
| contig00137 | BGIBMGA003332 | IPR001910 | Inosine/uridine-preferring |
| contig00137 | BGIBMGA003330 | IPR001910 | Inosine/uridine-preferring |
| contig00137 | BGIBMGA003331 | IPR001910 | Inosine/uridine-preferring |
| contig00138 | BGIBMGA000398 | IPR004165 | Coenzyme |
| contig00149 | BGIBMGA000664 | IPR008506 | Protein |
| contig00157 | BGIBMGA004908 | IPR005937 | 26S |
| contig00157 | BGIBMGA004782 | IPR005937 | 26S |
| contig00157 | BGIBMGA014177 | IPR003959 | AAA |
| contig00157 | BGIBMGA007332 | IPR003960 | AAA-protein |
| contig00157 | BGIBMGA010794 | IPR005937 | 26S |
| contig00164 | BGIBMGA006907 | IPR004790 | Isocitrate |
| contig00164 | BGIBMGA007586 | IPR013814 | Isocitrate/isopropylmalate |
| contig00182 | BGIBMGA003186 | IPR014014 | DEAD-box |
| contig00182 | BGIBMGA004822 | IPR014014 | DEAD-box |
| contig00182 | BGIBMGA010673 | IPR014001 | DEAD-like |
| contig00185 | BGIBMGA011029 | IPR002155 | Thiolase |
| contig00186 | BGIBMGA011922 | IPR011032 | GroES-like, |
| contig00188 | BGIBMGA010173 | IPR001857 | Ribosomal |
| contig00191 | BGIBMGA008868 | IPR002110 | Ankyrin |
| contig00195 | BGIBMGA005928 | IPR001854 | Ribosomal |
| contig00201 | BGIBMGA006179 | IPR002469 | Peptidase |
| contig00204 | BGIBMGA006840 | IPR012340 | Nucleic |
| contig00208 | BGIBMGA013777 | IPR004345 | TB2/DP1 |
| contig00214 | BGIBMGA012954 | IPR005290 | Ribosomal |
| contig00219 | BGIBMGA002640 | IPR001578 | Peptidase |
| contig00223 | BGIBMGA011696 | undefined |  |
| contig00225 | BGIBMGA009021 | IPR007052 | CS, |
| contig00226 | BGIBMGA006269 | IPR009066 | Alpha-2-macroglobulin |
| contig00228 | BGIBMGA012537 | undefined |  |
| contig00232 | BGIBMGA007469 | IPR008991 | Translation |
| contig00237 | BGIBMGA011584 | IPR011339 | FeS |
| contig00238 | BGIBMGA004830 | IPR000834 | Peptidase |
| contig00246 | BGIBMGA005023 | undefined |  |
| contig00253 | BGIBMGA004913 | IPR009025 | RNA |
| contig00257 | BGIBMGA004965 | IPR002213 | UDP-glucuronosyl/UDP-glucosyltransferase, |
| contig00263 | BGIBMGA012360 | undefined |  |
| contig00271 | BGIBMGA004806 | IPR002345 | Lipocalin, |
| contig00273 | BGIBMGA012866 | IPR006619 | Animal |
| contig00275 | BGIBMGA009033 | IPR002198 | Short-chain |
| contig00275 | BGIBMGA013160 | IPR002347 | Glucose/ribitol |
| contig00275 | BGIBMGA013270 | IPR002347 | Glucose/ribitol |
| contig00275 | BGIBMGA013220 | IPR002198 | Short-chain |
| contig00275 | BGIBMGA013271 | IPR002347 | Glucose/ribitol |
| contig00275 | BGIBMGA013221 | IPR002198 | Short-chain |
| contig00275 | BGIBMGA008509 | IPR002198 | Short-chain |
| contig00275 | BGIBMGA013192 | IPR002198 | Short-chain |
| contig00275 | BGIBMGA010193 | IPR002347 | Glucose/ribitol |
| contig00275 | BGIBMGA013193 | IPR002198 | Short-chain |
| contig00275 | BGIBMGA013161 | IPR002347 | Glucose/ribitol |
| contig00275 | BGIBMGA008889 | IPR002198 | Short-chain |
| contig00275 | BGIBMGA001323 | IPR002347 | Glucose/ribitol |
| contig00275 | BGIBMGA010512 | IPR002198 | Short-chain |
| contig00275 | BGIBMGA008096 | IPR002198 | Short-chain |
| contig00280 | BGIBMGA001236 | IPR005631 | Protein |
| contig00281 | BGIBMGA010129 | IPR013283 | ABC |
| contig00283 | BGIBMGA007092 | IPR001715 | Calponin-like |
| contig00283 | BGIBMGA008050 | IPR001715 | Calponin-like |
| contig00289 | BGIBMGA002186 | IPR012335 | Thioredoxin |
| contig00289 | BGIBMGA002406 | IPR012336 | Thioredoxin-like |
| contig00289 | BGIBMGA000064 | IPR000866 | Alkyl |
| contig00290 | BGIBMGA000918 | IPR001232 | SKP1 |
| contig00292 | BGIBMGA003475 | IPR001326 | Translation |
| contig00296 | BGIBMGA003924 | IPR001450 | 4Fe-4S |
| contig00302 | BGIBMGA001537 | IPR010793 | Ribosomal |
| contig00306 | BGIBMGA011824 | IPR000182 | GCN5-related |
| contig00308 | BGIBMGA012624 | IPR006094 | FAD |
| contig00312 | BGIBMGA003328 | IPR001283 | Allergen |
| contig00316 | BGIBMGA000382 | IPR007052 | CS, |
| contig00321 | BGIBMGA009951 | IPR010987 | Glutathione |
| contig00326 | BGIBMGA008815 | IPR002048 | Calcium-binding |
| contig00326 | BGIBMGA008814 | IPR002048 | Calcium-binding |
| contig00326 | BGIBMGA006444 | IPR002048 | Calcium-binding |
| contig00326 | BGIBMGA008813 | IPR002048 | Calcium-binding |
| contig00339 | BGIBMGA002939 | IPR002198 | Short-chain |
| contig00365 | BGIBMGA011108 | IPR002347 | Glucose/ribitol |
| contig00365 | BGIBMGA011785 | IPR002198 | Short-chain |
| contig00373 | BGIBMGA007962 | IPR002942 | RNA-binding |
| contig00379 | BGIBMGA002669 | IPR000734 | Lipase, |
| contig00385 | BGIBMGA002570 | IPR007676 | Ribophorin |
| contig00400 | BGIBMGA000837 | IPR002018 | Carboxylesterase, |
| contig00400 | BGIBMGA003517 | IPR002018 | Carboxylesterase, |
| contig00400 | BGIBMGA008141 | IPR002018 | Carboxylesterase, |
| contig00400 | BGIBMGA002901 | IPR002018 | Carboxylesterase, |
| contig00400 | BGIBMGA002796 | IPR002018 | Carboxylesterase, |
| contig00400 | BGIBMGA001629 | IPR002018 | Carboxylesterase, |
| contig00400 | BGIBMGA002902 | IPR002018 | Carboxylesterase, |
| contig00400 | BGIBMGA007670 | IPR002018 | Carboxylesterase, |
| contig00400 | BGIBMGA001630 | IPR002018 | Carboxylesterase, |
| contig00400 | BGIBMGA000875 | IPR002018 | Carboxylesterase, |
| contig00400 | BGIBMGA007671 | IPR008262 | Lipase, |
| contig00400 | BGIBMGA002899 | IPR002168 | Lipolytic |
| contig00400 | BGIBMGA004229 | IPR002018 | Carboxylesterase, |
| contig00400 | BGIBMGA012722 | IPR002018 | Carboxylesterase, |
| contig00400 | BGIBMGA012729 | IPR002018 | Carboxylesterase, |
| contig00400 | BGIBMGA012728 | IPR002018 | Carboxylesterase, |
| contig00400 | BGIBMGA004147 | IPR002168 | Lipolytic |
| contig00400 | BGIBMGA011218 | IPR002018 | Carboxylesterase, |
| contig00400 | BGIBMGA010987 | IPR002018 | Carboxylesterase, |
| contig00400 | BGIBMGA004205 | IPR002018 | Carboxylesterase, |
| contig00400 | BGIBMGA004684 | IPR002018 | Carboxylesterase, |
| contig00400 | BGIBMGA012031 | IPR002018 | Carboxylesterase, |
| contig00400 | BGIBMGA004934 | IPR002018 | Carboxylesterase, |
| contig00400 | BGIBMGA010988 | IPR002018 | Carboxylesterase, |
| contig00400 | BGIBMGA004683 | IPR002018 | Carboxylesterase, |
| contig00400 | BGIBMGA012122 | IPR002018 | Carboxylesterase, |
| contig00400 | BGIBMGA002698 | IPR002018 | Carboxylesterase, |
| contig00400 | BGIBMGA010976 | IPR002018 | Carboxylesterase, |
| contig00400 | BGIBMGA004206 | IPR002018 | Carboxylesterase, |
| contig00400 | BGIBMGA011893 | IPR002018 | Carboxylesterase, |
| contig00400 | BGIBMGA007546 | IPR002018 | Carboxylesterase, |
| contig00400 | BGIBMGA005195 | IPR002110 | Ankyrin, |
| contig00400 | BGIBMGA000777 | IPR002018 | Carboxylesterase, |
| contig00400 | BGIBMGA007547 | IPR002018 | Carboxylesterase, |
| contig00400 | BGIBMGA014599 | IPR002018 | Carboxylesterase, |
| contig00400 | BGIBMGA010986 | IPR002018 | Carboxylesterase, |
| contig00400 | BGIBMGA010328 | IPR002018 | Carboxylesterase, |
| contig00400 | BGIBMGA010505 | IPR002290 | Serine/threonine |
| contig00425 | BGIBMGA004112 | IPR005822 | Ribosomal |
| contig00432 | BGIBMGA010877 | IPR004943 | Lepidopteran |
| contig00432 | BGIBMGA009573 | IPR004943 | Lepidopteran |
| contig00432 | BGIBMGA004468 | IPR004943 | Lepidopteran |
| contig00432 | BGIBMGA004464 | IPR004943 | Lepidopteran |
| contig00432 | BGIBMGA009621 | undefined |  |
| contig00432 | BGIBMGA008165 | IPR004943 | Lepidopteran |
| contig00432 | BGIBMGA010876 | IPR004943 | Lepidopteran |
| contig00432 | BGIBMGA010168 | IPR004943 | Lepidopteran |
| contig00432 | BGIBMGA004463 | IPR004943 | Lepidopteran |
| contig00432 | BGIBMGA008164 | IPR004943 | Lepidopteran |
| contig00432 | BGIBMGA004395 | IPR004943 | Lepidopteran |
| contig00432 | BGIBMGA004396 | IPR004943 | Lepidopteran |
| contig00432 | BGIBMGA010204 | IPR004943 | Lepidopteran |
| contig00432 | BGIBMGA004397 | IPR004943 | Lepidopteran |
| contig00432 | BGIBMGA004404 | IPR004943 | Lepidopteran |
| contig00432 | BGIBMGA004455 | IPR004943 | Lepidopteran |
| contig00432 | BGIBMGA004456 | IPR004943 | Lepidopteran |
| contig00432 | BGIBMGA004403 | IPR004943 | Lepidopteran |
| contig00432 | BGIBMGA004467 | IPR004943 | Lepidopteran |
| contig00432 | BGIBMGA004465 | IPR004943 | Lepidopteran |
| contig00432 | BGIBMGA004399 | IPR004943 | Lepidopteran |
| contig00432 | BGIBMGA004400 | IPR004943 | Lepidopteran |
| contig00432 | BGIBMGA004394 | IPR004943 | Lepidopteran |
| contig00433 | BGIBMGA011786 | IPR004045 | Glutathione |
| contig00449 | BGIBMGA014202 | IPR009947 | NADH:ubiquinone |
| contig00460 | BGIBMGA007228 | IPR000175 | Sodium:neurotransmitter |
| contig00460 | BGIBMGA000927 | IPR000175 | Sodium:neurotransmitter |
| contig00460 | BGIBMGA006164 | IPR000175 | Sodium:neurotransmitter |
| contig00460 | BGIBMGA006857 | IPR000175 | Sodium:neurotransmitter |
| contig00460 | BGIBMGA004570 | IPR000175 | Sodium:neurotransmitter |
| contig00460 | BGIBMGA014231 | IPR000175 | Sodium:neurotransmitter |
| contig00460 | BGIBMGA006619 | IPR000175 | Sodium:neurotransmitter |
| contig00460 | BGIBMGA004216 | IPR000175 | Sodium:neurotransmitter |
| contig00460 | BGIBMGA004312 | IPR000433 | Zinc |
| contig00472 | BGIBMGA008892 | IPR000361 | HesB/YadR/YfhF |
| contig00476 | BGIBMGA005853 | IPR002048 | Calcium-binding |
| contig00481 | BGIBMGA002981 | IPR002097 | Profilin/allergen, |
| contig00493 | BGIBMGA005615 | IPR013154 | Alcohol |
| contig00507 | BGIBMGA001276 | IPR001128 | Cytochrome |
| contig00507 | BGIBMGA001277 | IPR001128 | Cytochrome |
| contig00507 | BGIBMGA005356 | IPR001128 | Cytochrome |
| contig00509 | BGIBMGA012385 | IPR001128 | Cytochrome |
| contig00509 | BGIBMGA012089 | IPR001128 | Cytochrome |
| contig00509 | BGIBMGA010854 | IPR001128 | Cytochrome |
| contig00509 | BGIBMGA012386 | IPR001128 | Cytochrome |
| contig00509 | BGIBMGA006691 | IPR002403 | E-class |
| contig00509 | BGIBMGA013241 | IPR001128 | Cytochrome |
| contig00509 | BGIBMGA013237 | IPR002401 | E-class |
| contig00509 | BGIBMGA013239 | IPR001128 | Cytochrome |
| contig00509 | BGIBMGA013238 | IPR002401 | E-class |
| contig00509 | BGIBMGA001573 | IPR001128 | Cytochrome |
| contig00509 | BGIBMGA006785 | IPR001128 | Cytochrome |
| contig00509 | BGIBMGA003944 | IPR001128 | Cytochrome |
| contig00509 | BGIBMGA003943 | IPR001128 | Cytochrome |
| contig00509 | BGIBMGA003945 | IPR001128 | Cytochrome |
| contig00509 | BGIBMGA003926 | IPR001128 | Cytochrome |
| contig00509 | BGIBMGA003957 | IPR001128 | Cytochrome |
| contig00509 | BGIBMGA003293 | IPR001128 | Cytochrome |
| contig00509 | BGIBMGA001419 | IPR001128 | Cytochrome |
| contig00509 | BGIBMGA011545 | IPR001128 | Cytochrome |
| contig00509 | BGIBMGA002178 | IPR001128 | Cytochrome |
| contig00512 | BGIBMGA010139 | IPR000592 | Ribosomal |
| contig00515 | BGIBMGA005318 | IPR000372 | Leucine-rich |
| contig00516 | BGIBMGA004687 | IPR000719 | Protein |
| contig00518 | BGIBMGA002984 | IPR006195 | Aminoacyl-transfer |
| contig00519 | BGIBMGA009760 | IPR000804 | Clathrin |
| contig00522 | BGIBMGA008059 | IPR006025 | Peptidase |
| contig00526 | BGIBMGA007553 | IPR001304 | C-type |
| contig00531 | BGIBMGA000068 | IPR000172 | Glucose-methanol-choline |
| contig00531 | BGIBMGA005710 | IPR000172 | Glucose-methanol-choline |
| contig00531 | BGIBMGA000158 | IPR000172 | Glucose-methanol-choline |
| contig00531 | BGIBMGA010448 | IPR012132 | Glucose-methanol-choline |
| contig00531 | BGIBMGA005711 | IPR000172 | Glucose-methanol-choline |
| contig00531 | BGIBMGA010461 | IPR000172 | Glucose-methanol-choline |
| contig00531 | BGIBMGA012999 | IPR000172 | Glucose-methanol-choline |
| contig00531 | BGIBMGA013000 | IPR000172 | Glucose-methanol-choline |
| contig00531 | BGIBMGA009242 | IPR012132 | Glucose-methanol-choline |
| contig00531 | BGIBMGA010515 | IPR012132 | Glucose-methanol-choline |
| contig00531 | BGIBMGA012997 | IPR000172 | Glucose-methanol-choline |
| contig00531 | BGIBMGA012115 | IPR000172 | Glucose-methanol-choline |
| contig00531 | BGIBMGA012998 | IPR000172 | Glucose-methanol-choline |
| contig00531 | BGIBMGA014539 | IPR000172 | Glucose-methanol-choline |
| contig00531 | BGIBMGA005703 | IPR000172 | Glucose-methanol-choline |
| contig00531 | BGIBMGA010516 | IPR012132 | Glucose-methanol-choline |
| contig00531 | BGIBMGA012586 | IPR012132 | Glucose-methanol-choline |
| contig00531 | BGIBMGA013788 | IPR000172 | Glucose-methanol-choline |
| contig00531 | BGIBMGA013003 | IPR000172 | Glucose-methanol-choline |
| contig00531 | BGIBMGA013951 | IPR000172 | Glucose-methanol-choline |
| contig00531 | BGIBMGA012618 | IPR012132 | Glucose-methanol-choline |
| contig00531 | BGIBMGA005609 | IPR000172 | Glucose-methanol-choline |
| contig00532 | BGIBMGA010365 | IPR001522 | Fatty |
| contig00532 | BGIBMGA010681 | IPR001522 | Fatty |
| contig00532 | BGIBMGA009556 | IPR010257 | Fatty |
| contig00532 | BGIBMGA008171 | IPR001522 | Fatty |
| contig00532 | BGIBMGA005471 | IPR005804 | Fatty |
| contig00532 | BGIBMGA009568 | IPR001522 | Fatty |
| contig00532 | BGIBMGA010614 | IPR001522 | Fatty |
| contig00532 | BGIBMGA010676 | IPR001522 | Fatty |
| contig00532 | BGIBMGA011563 | IPR001522 | Fatty |
| contig00532 | BGIBMGA006471 | IPR001522 | Fatty |
| contig00532 | BGIBMGA010611 | IPR001522 | Fatty |
| contig00532 | BGIBMGA004868 | IPR012196 | Acyl-CoA |
| contig00532 | BGIBMGA006470 | IPR012196 | Acyl-CoA |
| contig00532 | BGIBMGA014550 | IPR001522 | Fatty |
| contig00532 | BGIBMGA006469 | IPR001522 | Fatty |
| contig00538 | BGIBMGA004001 | IPR008260 | Hydroxymethylglutaryl-coenzyme |
| contig00551 | BGIBMGA006733 | IPR000463 | Cytosolic |
| contig00563 | BGIBMGA005506 | IPR011032 | GroES-like, |
| contig00563 | BGIBMGA005505 | IPR013149 | Alcohol |
| contig00563 | BGIBMGA007100 | IPR013149 | Alcohol |
| contig00563 | BGIBMGA007098 | IPR011032 | GroES-like, |
| contig00565 | BGIBMGA005808 | IPR002198 | Short-chain |
| contig00565 | BGIBMGA005763 | IPR002198 | Short-chain |
| contig00566 | BGIBMGA011658 | IPR005442 | Glutathione |
| contig00566 | BGIBMGA011820 | IPR005442 | Glutathione |
| contig00566 | BGIBMGA011819 | IPR005442 | Glutathione |
| contig00567 | BGIBMGA011438 | IPR012335 | Thioredoxin |
| contig00567 | BGIBMGA013994 | IPR012336 | Thioredoxin-like |
| contig00573 | BGIBMGA005161 | IPR000182 | GCN5-related |
| contig00575 | BGIBMGA011334 | IPR001152 | Thymosin |
| contig00581 | BGIBMGA007516 | IPR009001 | EF-Tu/eEF-1alpha/eIF2-gamma, |
| contig00581 | BGIBMGA003608 | IPR000795 | Protein |
| contig00587 | BGIBMGA004331 | IPR001179 | Peptidylprolyl |
| contig00588 | BGIBMGA003608 | IPR000795 | Protein |
| contig00588 | BGIBMGA007516 | IPR009001 | EF-Tu/eEF-1alpha/eIF2-gamma, |
| contig00594 | BGIBMGA014214 | IPR002108 | Actin-binding, |
| contig00603 | BGIBMGA007517 | IPR000583 | Glutamine |
| contig00605 | BGIBMGA004806 | IPR002345 | Lipocalin, |
| contig00607 | BGIBMGA007915 | IPR006025 | Peptidase |
| contig00607 | BGIBMGA007916 | IPR006025 | Peptidase |
| contig00609 | BGIBMGA011416 | IPR001593 | Ribosomal |
| contig00610 | BGIBMGA002670 | IPR000734 | Lipase, |
| contig00610 | BGIBMGA002669 | IPR000734 | Lipase, |
| contig00610 | BGIBMGA001508 | IPR000734 | Lipase, |
| contig00610 | BGIBMGA001507 | IPR000734 | Lipase, |
| contig00610 | BGIBMGA001506 | IPR013818 | Lipase, |
| contig00610 | BGIBMGA011895 | IPR013818 | Lipase, |
| contig00610 | BGIBMGA003664 | IPR008262 | Lipase, |
| contig00610 | BGIBMGA010400 | IPR000734 | Lipase, |
| contig00612 | BGIBMGA010604 | IPR001975 | Ribosomal |
| contig00613 | BGIBMGA000623 | IPR013098 | Immunoglobulin |
| contig00615 | BGIBMGA008780 | IPR008331 | Ferritin |
| contig00619 | BGIBMGA010541 | IPR000608 | Ubiquitin-conjugating |
| contig00619 | BGIBMGA002918 | IPR000608 | Ubiquitin-conjugating |
| contig00622 | BGIBMGA009028 | IPR013788 | Arthropod |
| contig00622 | BGIBMGA009027 | IPR000896 | Hemocyanin, |
| contig00623 | BGIBMGA009027 | IPR000896 | Hemocyanin, |
| contig00623 | BGIBMGA009028 | IPR013788 | Arthropod |
| contig00624 | BGIBMGA009027 | IPR000896 | Hemocyanin, |
| contig00624 | BGIBMGA009028 | IPR013788 | Arthropod |
| contig00639 | BGIBMGA008768 | IPR001519 | Ferritin, |
| contig00646 | BGIBMGA005954 | IPR001251 | Cellular |
| contig00646 | BGIBMGA005953 | IPR001251 | Cellular |
| contig00655 | BGIBMGA014177 | IPR003959 | AAA |
| contig00659 | BGIBMGA013895 | IPR001369 | Purine |
| contig00660 | BGIBMGA003057 | IPR006589 | Glycosyl |
| contig00660 | BGIBMGA003056 | IPR006047 | Glycosyl |
| contig00660 | BGIBMGA003055 | IPR006589 | Glycosyl |
| contig00660 | BGIBMGA006066 | IPR006047 | Glycosyl |
| contig00667 | BGIBMGA004059 | IPR002130 | Peptidyl-prolyl |
| contig00677 | BGIBMGA013413 | IPR000217 | Tubulin, |
| contig00677 | BGIBMGA002103 | IPR000217 | Tubulin, |
| contig00677 | BGIBMGA002542 | IPR002452 | Alpha |
| contig00677 | BGIBMGA004681 | IPR003008 | Tubulin/FtsZ, |
| contig00677 | BGIBMGA009132 | IPR013838 | Beta |
| contig00677 | BGIBMGA004603 | IPR002453 | Beta |
| contig00677 | BGIBMGA003442 | IPR002453 | Beta |
| contig00677 | BGIBMGA003296 | IPR000217 | Tubulin, |
| contig00677 | BGIBMGA001683 | IPR002453 | Beta |
| contig00677 | BGIBMGA009133 | IPR002453 | Beta |
| contig00677 | BGIBMGA001707 | IPR002453 | Beta |
| contig00677 | BGIBMGA004542 | IPR002453 | Beta |
| contig00677 | BGIBMGA013500 | IPR000217 | Tubulin, |
| contig00677 | BGIBMGA009131 | IPR002453 | Beta |
| contig00693 | BGIBMGA011936 | IPR008689 | ATPase, |
| contig00693 | BGIBMGA004942 | IPR008689 | ATPase, |
| contig00694 | BGIBMGA003943 | IPR001128 | Cytochrome |
| contig00694 | BGIBMGA003944 | IPR001128 | Cytochrome |
| contig00694 | BGIBMGA003945 | IPR001128 | Cytochrome |
| contig00694 | BGIBMGA003957 | IPR001128 | Cytochrome |
| contig00694 | BGIBMGA002171 | IPR001128 | Cytochrome |
| contig00703 | BGIBMGA008082 | IPR006088 | Sterol |
| contig00704 | BGIBMGA011822 | IPR012971 | NGP1, |
| contig00706 | BGIBMGA008504 | IPR001611 | Leucine-rich |
| contig00713 | BGIBMGA002429 | IPR002130 | Peptidyl-prolyl |
| contig00713 | BGIBMGA003522 | IPR002130 | Peptidyl-prolyl |
| contig00713 | BGIBMGA004059 | IPR002130 | Peptidyl-prolyl |
| contig00713 | BGIBMGA006771 | IPR002130 | Peptidyl-prolyl |
| contig00713 | BGIBMGA003415 | IPR002130 | Peptidyl-prolyl |
| contig00720 | BGIBMGA012089 | IPR001128 | Cytochrome |
| contig00720 | BGIBMGA012385 | IPR001128 | Cytochrome |
| contig00720 | BGIBMGA010854 | IPR001128 | Cytochrome |
| contig00720 | BGIBMGA013237 | IPR002401 | E-class |
| contig00720 | BGIBMGA006691 | IPR002403 | E-class |
| contig00720 | BGIBMGA012386 | IPR001128 | Cytochrome |
| contig00720 | BGIBMGA013241 | IPR001128 | Cytochrome |
| contig00720 | BGIBMGA013239 | IPR001128 | Cytochrome |
| contig00720 | BGIBMGA013238 | IPR002401 | E-class |
| contig00723 | BGIBMGA002381 | IPR001023 | Heat |
| contig00723 | BGIBMGA006313 | IPR001023 | Heat |
| contig00723 | BGIBMGA014536 | IPR013126 | Heat |
| contig00723 | BGIBMGA007950 | IPR000886 | Endoplasmic |
| contig00723 | BGIBMGA001635 | IPR001023 | Heat |
| contig00733 | BGIBMGA013700 | undefined |  |
| contig00737 | BGIBMGA007098 | IPR011032 | GroES-like, |
| contig00737 | BGIBMGA007100 | IPR013149 | Alcohol |
| contig00737 | BGIBMGA007099 | IPR013149 | Alcohol |
| contig00737 | BGIBMGA005506 | IPR011032 | GroES-like, |
| contig00737 | BGIBMGA005505 | IPR013149 | Alcohol |
| contig00738 | BGIBMGA011061 | IPR007853 | Zinc |
| contig00748 | BGIBMGA003469 | IPR000504 | RNA-binding |
| contig00749 | BGIBMGA010198 | undefined |  |
| contig00750 | BGIBMGA010970 | IPR008991 | Translation |
| contig00754 | BGIBMGA012134 | IPR008974 | TRAF-like, |
| contig00757 | BGIBMGA003474 | IPR000719 | Protein |
| contig00758 | BGIBMGA014483 | IPR008992 | Bacterial |
| contig00772 | BGIBMGA009055 | IPR011701 | Major |
| contig00774 | BGIBMGA011281 | IPR007194 | Transport |
| contig00776 | BGIBMGA010751 | IPR002715 | Nascent |
| contig00777 | BGIBMGA013497 | IPR008092 | Death |
| contig00778 | BGIBMGA002570 | IPR007676 | Ribophorin |
| contig00785 | BGIBMGA009864 | IPR008417 | B-cell |
| contig00787 | BGIBMGA012089 | IPR001128 | Cytochrome |
| contig00787 | BGIBMGA003945 | IPR001128 | Cytochrome |
| contig00787 | BGIBMGA001573 | IPR001128 | Cytochrome |
| contig00787 | BGIBMGA003943 | IPR001128 | Cytochrome |
| contig00787 | BGIBMGA012385 | IPR001128 | Cytochrome |
| contig00787 | BGIBMGA003944 | IPR001128 | Cytochrome |
| contig00787 | BGIBMGA013237 | IPR002401 | E-class |
| contig00788 | BGIBMGA003693 | IPR000873 | AMP-dependent |
| contig00788 | BGIBMGA009675 | IPR000873 | AMP-dependent |
| contig00788 | BGIBMGA004920 | IPR000871 | Beta-lactamase, |
| contig00788 | BGIBMGA009676 | IPR000873 | AMP-dependent |
| contig00794 | BGIBMGA013945 | IPR004001 | Actin, |
| contig00794 | BGIBMGA009821 | IPR004000 | Actin/actin-like, |
| contig00794 | BGIBMGA005576 | IPR004000 | Actin/actin-like, |
| contig00794 | BGIBMGA012151 | IPR004001 | Actin, |
| contig00794 | BGIBMGA000082 | IPR004000 | Actin/actin-like |
| contig00794 | BGIBMGA002082 | IPR004000 | Actin/actin-like |
| contig00794 | BGIBMGA005577 | IPR004000 | Actin/actin-like, |
| contig00794 | BGIBMGA001921 | IPR004000 | Actin/actin-like |
| contig00794 | BGIBMGA002083 | IPR004000 | Actin/actin-like |
| contig00794 | BGIBMGA009805 | IPR004000 | Actin/actin-like |
| contig00796 | BGIBMGA002617 | IPR001611 | Leucine-rich |
| contig00797 | BGIBMGA006966 | IPR002075 | Nuclear |
| contig00799 | BGIBMGA007881 | IPR008699 | NADH-ubiquinone |
| contig00809 | BGIBMGA003683 | IPR001128 | Cytochrome |
| contig00809 | BGIBMGA014043 | IPR001128 | Cytochrome |
| contig00809 | BGIBMGA001162 | IPR002401 | E-class |
| contig00809 | BGIBMGA007168 | IPR001128 | Cytochrome |
| contig00809 | BGIBMGA001004 | IPR001128 | Cytochrome |
| contig00809 | BGIBMGA001003 | IPR001128 | Cytochrome |
| contig00809 | BGIBMGA007195 | IPR001128 | Cytochrome |
| contig00809 | BGIBMGA014046 | IPR001128 | Cytochrome |
| contig00809 | BGIBMGA001005 | IPR001128 | Cytochrome |
| contig00809 | BGIBMGA002307 | IPR001128 | Cytochrome |
| contig00809 | BGIBMGA002267 | IPR001128 | Cytochrome |
| contig00809 | BGIBMGA004717 | IPR002401 | E-class |
| contig00810 | BGIBMGA007892 | undefined |  |
| contig00812 | BGIBMGA006751 | IPR002041 | GTP-binding |
| contig00813 | BGIBMGA012032 | IPR001476 | Chaperonin |
| contig00821 | BGIBMGA001964 | IPR000886 | Endoplasmic |
| contig00823 | BGIBMGA012373 | IPR000988 | Ribosomal |
| contig00828 | BGIBMGA005559 | IPR011001 | Saposin-like |
| contig00831 | BGIBMGA006154 | IPR006603 | Cystinosin/ERS1p |
| contig00851 | BGIBMGA011524 | IPR001370 | Proteinase |
| contig00853 | BGIBMGA001339 | IPR008518 | Protein |
| contig00867 | BGIBMGA001274 | IPR003137 | Protease-associated |
| contig00871 | BGIBMGA002984 | IPR006195 | Aminoacyl-transfer |
| contig00875 | BGIBMGA004798 | IPR000834 | Peptidase |
| contig00875 | BGIBMGA004797 | IPR000834 | Peptidase |
| contig00875 | BGIBMGA004801 | IPR000834 | Peptidase |
| contig00875 | BGIBMGA008910 | IPR003146 | Proteinase |
| contig00875 | BGIBMGA006715 | IPR000834 | Peptidase |
| contig00875 | BGIBMGA008976 | IPR009020 | Proteinase |
| contig00875 | BGIBMGA004799 | IPR000834 | Peptidase |
| contig00875 | BGIBMGA009487 | IPR009020 | Proteinase |
| contig00875 | BGIBMGA004800 | IPR000834 | Peptidase |
| contig00875 | BGIBMGA006871 | IPR003146 | Proteinase |
| contig00878 | BGIBMGA007824 | IPR005809 | Succinyl-CoA |
| contig00881 | BGIBMGA001194 | IPR001412 | Aminoacyl-tRNA |
| contig00884 | BGIBMGA006823 | IPR005814 | Aminotransferase |
| contig00887 | BGIBMGA004488 | IPR008011 | Complex |
| contig00891 | BGIBMGA008542 | IPR002699 | ATPase, |
| contig00895 | BGIBMGA009953 | IPR000215 | Proteinase |
| contig00895 | BGIBMGA007720 | IPR000215 | Proteinase |
| contig00905 | BGIBMGA003070 | IPR004217 | Zinc |
| contig00909 | BGIBMGA001595 | IPR003010 | Nitrilase/cyanide |
| contig00909 | BGIBMGA001536 | IPR003010 | Nitrilase/cyanide |
| contig00924 | BGIBMGA011593 | IPR000701 | Succinate |
| contig00924 | BGIBMGA011424 | IPR001156 | Peptidase |
| contig00925 | BGIBMGA011783 | IPR004241 | Light |
| contig00929 | BGIBMGA008864 | IPR002110 | Ankyrin |
| contig00931 | BGIBMGA010222 | IPR000608 | Ubiquitin-conjugating |
| contig00932 | BGIBMGA006631 | IPR007248 | Mpv17/PMP22 |
| contig00934 | BGIBMGA005673 | IPR006693 | AB-hydrolase |
| contig00934 | BGIBMGA011777 | IPR008262 | Lipase, |
| contig00934 | BGIBMGA010639 | IPR008262 | Lipase, |
| contig00934 | BGIBMGA010640 | IPR008262 | Lipase, |
| contig00934 | BGIBMGA004200 | IPR006693 | AB-hydrolase |
| contig00934 | BGIBMGA004157 | IPR008262 | Lipase, |
| contig00934 | BGIBMGA011072 | IPR008262 | Lipase, |
| contig00934 | BGIBMGA005695 | IPR006693 | AB-hydrolase |
| contig00934 | BGIBMGA004708 | IPR006693 | AB-hydrolase |
| contig00934 | BGIBMGA006149 | IPR006693 | AB-hydrolase |
| contig00934 | BGIBMGA012745 | IPR006693 | AB-hydrolase |
| contig00934 | BGIBMGA012649 | IPR006693 | AB-hydrolase |
| contig00934 | BGIBMGA014574 | IPR006693 | AB-hydrolase |
| contig00934 | BGIBMGA014575 | IPR006693 | AB-hydrolase |
| contig00934 | BGIBMGA009179 | IPR006693 | AB-hydrolase |
| contig00934 | BGIBMGA014267 | IPR006693 | AB-hydrolase |
| contig00934 | BGIBMGA012648 | IPR006693 | AB-hydrolase |
| contig00934 | BGIBMGA014239 | IPR006693 | AB-hydrolase |
| contig00934 | BGIBMGA012634 | IPR000073 | Alpha/beta |
| contig00934 | BGIBMGA012647 | IPR006693 | AB-hydrolase |
| contig00937 | BGIBMGA008312 | IPR000163 | Prohibitin, |
| contig00941 | BGIBMGA005687 | IPR005002 | Eukaryotic |
| contig00943 | BGIBMGA013757 | IPR002509 | Polysaccharide |
| contig00943 | BGIBMGA013756 | IPR002509 | Polysaccharide |
| contig00943 | BGIBMGA013758 | IPR002509 | Polysaccharide |
| contig00943 | BGIBMGA002696 | IPR002509 | Polysaccharide |
| contig00943 | BGIBMGA006213 | IPR002557 | Chitin |
| contig00943 | BGIBMGA006214 | IPR002172 | Low |
| contig00943 | BGIBMGA008988 | IPR002172 | Low |
| contig00943 | BGIBMGA010573 | IPR002557 | Chitin |
| contig00953 | BGIBMGA009821 | IPR004000 | Actin/actin-like, |
| contig00953 | BGIBMGA005576 | IPR004000 | Actin/actin-like, |
| contig00953 | BGIBMGA013945 | IPR004001 | Actin, |
| contig00953 | BGIBMGA005577 | IPR004000 | Actin/actin-like, |
| contig00955 | BGIBMGA008861 | IPR003299 | Flagellar |
| contig00957 | BGIBMGA004891 | IPR009038 | GOLD |
| contig00961 | BGIBMGA014123 | IPR013657 | UAA |
| contig00963 | BGIBMGA000425 | IPR002123 | Phospholipid/glycerol |
| contig00964 | BGIBMGA001501 | IPR001377 | Ribosomal |
| contig00965 | BGIBMGA009799 | IPR001395 | Aldo/keto |
| contig00965 | BGIBMGA009800 | IPR001395 | Aldo/keto |
| contig00966 | BGIBMGA008881 | IPR002132 | Ribosomal |
| contig00974 | BGIBMGA006842 | IPR012337 | Polynucleotidyl |
| contig00979 | BGIBMGA010934 | IPR011701 | Major |
| contig00982 | BGIBMGA006990 | undefined |  |
| contig00984 | BGIBMGA011099 | IPR001611 | Leucine-rich |
| contig00984 | BGIBMGA011100 | IPR013513 | Integrin |
| contig00987 | BGIBMGA005169 | IPR009088 | Transcription |
| contig00994 | BGIBMGA003197 | IPR001892 | Ribosomal |
| contig01002 | BGIBMGA010571 | IPR000702 | Ribosomal |
| contig01009 | BGIBMGA002582 | IPR011765 | Peptidase |
| contig01009 | BGIBMGA002052 | IPR011765 | Peptidase |
| contig01009 | BGIBMGA002081 | IPR011765 | Peptidase |
| contig01011 | BGIBMGA001386 | IPR000842 | Phosphoribosyl |
| contig01011 | BGIBMGA001570 | IPR000842 | Phosphoribosyl |
| contig01020 | BGIBMGA010423 | IPR008267 | Malate |
| contig01030 | BGIBMGA003725 | IPR000903 | Myristoyl-CoA:protein |
| contig01034 | BGIBMGA011712 | IPR011697 | Peptidase |
| contig01037 | BGIBMGA002288 | IPR001304 | C-type |
| contig01037 | BGIBMGA002289 | IPR001304 | C-type |
| contig01040 | BGIBMGA010247 | IPR002842 | ATPase, |
| contig01041 | BGIBMGA009359 | IPR008968 | Mu2 |
| contig01042 | BGIBMGA008556 | IPR000873 | AMP-dependent |
| contig01042 | BGIBMGA008557 | IPR000873 | AMP-dependent |
| contig01047 | BGIBMGA014094 | IPR002784 | Ribosomal |
| contig01048 | BGIBMGA009901 | IPR001878 | Zinc |
| contig01049 | BGIBMGA006369 | IPR006977 | Yip1 |
| contig01051 | BGIBMGA009332 | undefined |  |
| contig01055 | BGIBMGA001587 | IPR000533 | Tropomyosin, |
| contig01060 | BGIBMGA000470 | IPR002933 | Peptidase |
| contig01060 | BGIBMGA010352 | IPR001261 | ArgE/dapE/ACY1/CPG2/yscS, |
| contig01060 | BGIBMGA011002 | IPR001261 | ArgE/dapE/ACY1/CPG2/yscS, |
| contig01060 | BGIBMGA011000 | IPR001261 | ArgE/dapE/ACY1/CPG2/yscS, |
| contig01066 | BGIBMGA010471 | IPR001715 | Calponin-like |
| contig01067 | BGIBMGA002704 | IPR002345 | Lipocalin, |
| contig01072 | BGIBMGA010684 | IPR003177 | Cytochrome |
| contig01074 | BGIBMGA004375 | IPR012988 | Ribosomal |
| contig01076 | BGIBMGA006794 | IPR006214 | Protein |
| contig01085 | BGIBMGA003658 | IPR000683 | Oxidoreductase, |
| contig01085 | BGIBMGA003631 | IPR000683 | Oxidoreductase, |
| contig01090 | BGIBMGA002381 | IPR001023 | Heat |
| contig01090 | BGIBMGA014536 | IPR013126 | Heat |
| contig01090 | BGIBMGA004614 | IPR001023 | Heat |
| contig01090 | BGIBMGA014618 | IPR013126 | Heat |
| contig01090 | BGIBMGA004613 | IPR001023 | Heat |
| contig01090 | BGIBMGA006313 | IPR001023 | Heat |
| contig01094 | BGIBMGA009402 | IPR005533 | AMOP, |
| contig01101 | BGIBMGA007701 | IPR001564 | Nucleoside |
| contig01102 | BGIBMGA001173 | IPR001604 | DNA/RNA |
| contig01102 | BGIBMGA009447 | IPR003006 | Immunoglobulin/major |
| contig01104 | BGIBMGA010812 | IPR001360 | Glycoside |
| contig01104 | BGIBMGA010811 | IPR001360 | Glycoside |
| contig01106 | BGIBMGA008096 | IPR002198 | Short-chain |
| contig01106 | BGIBMGA008095 | IPR002198 | Short-chain |
| contig01106 | BGIBMGA001323 | IPR002347 | Glucose/ribitol |
| contig01106 | BGIBMGA010193 | IPR002347 | Glucose/ribitol |
| contig01106 | BGIBMGA009033 | IPR002198 | Short-chain |
| contig01106 | BGIBMGA008509 | IPR002198 | Short-chain |
| contig01106 | BGIBMGA013160 | IPR002347 | Glucose/ribitol |
| contig01106 | BGIBMGA008889 | IPR002198 | Short-chain |
| contig01106 | BGIBMGA013220 | IPR002198 | Short-chain |
| contig01106 | BGIBMGA013221 | IPR002198 | Short-chain |
| contig01118 | BGIBMGA013129 | IPR001139 | Glycoside |
| contig01118 | BGIBMGA013130 | IPR001139 | Glycoside |
| contig01118 | BGIBMGA001129 | IPR009057 | Homeodomain-like, |
| contig01119 | BGIBMGA005944 | IPR010990 | Transcription |
| contig01122 | BGIBMGA005599 | IPR001611 | Leucine-rich |
| contig01124 | BGIBMGA004612 | IPR009079 | Four-helical |
| contig01124 | BGIBMGA012753 | IPR001404 | Heat |
| contig01127 | BGIBMGA013680 | IPR013288 | Cytochrome |
| contig01127 | BGIBMGA013666 | IPR013288 | Cytochrome |
| contig01130 | BGIBMGA009487 | IPR009020 | Proteinase |
| contig01130 | BGIBMGA009486 | IPR009020 | Proteinase |
| contig01130 | BGIBMGA004830 | IPR000834 | Peptidase |
| contig01130 | BGIBMGA009478 | IPR009020 | Proteinase |
| contig01130 | BGIBMGA009477 | IPR009020 | Proteinase |
| contig01130 | BGIBMGA013275 | IPR000834 | Peptidase |
| contig01130 | BGIBMGA001891 | IPR000834 | Peptidase |
| contig01131 | BGIBMGA011482 | IPR000008 | C2 |
| contig01137 | BGIBMGA008402 | IPR013179 | Protein |
| contig01152 | BGIBMGA005329 | IPR007594 | RFT1 |
| contig01156 | BGIBMGA000585 | IPR002502 | N-acetylmuramoyl-L-alanine |
| contig01156 | BGIBMGA000584 | IPR002502 | N-acetylmuramoyl-L-alanine |
| contig01160 | BGIBMGA009051 | IPR013766 | Thioredoxin |
| contig01167 | BGIBMGA012934 | IPR011576 | Pyridoxamine |
| contig01174 | BGIBMGA002222 | IPR012335 | Thioredoxin |
| contig01174 | BGIBMGA002211 | IPR012335 | Thioredoxin |
| contig01174 | BGIBMGA006537 | IPR012335 | Thioredoxin |
| contig01177 | BGIBMGA000387 | IPR001611 | Leucine-rich |
| contig01178 | BGIBMGA013267 | IPR008914 | PEBP |
| contig01178 | BGIBMGA013261 | IPR008914 | PEBP |
| contig01179 | BGIBMGA008514 | IPR001254 | Peptidase |
| contig01179 | BGIBMGA007377 | IPR009003 | Peptidase, |
| contig01179 | BGIBMGA008515 | IPR009003 | Peptidase, |
| contig01179 | BGIBMGA014022 | IPR001254 | Peptidase |
| contig01179 | BGIBMGA009526 | IPR001254 | Peptidase |
| contig01180 | BGIBMGA001195 | undefined |  |
| contig01189 | BGIBMGA007940 | IPR001529 | DNA-directed |
| contig01190 | BGIBMGA001490 | IPR001179 | Peptidylprolyl |
| contig01193 | BGIBMGA011074 | IPR004911 | Gamma |
| contig01194 | BGIBMGA012887 | IPR001251 | Cellular |
| contig01196 | BGIBMGA010472 | IPR002017 | Spectrin |
| contig01200 | BGIBMGA002698 | IPR002018 | Carboxylesterase, |
| contig01200 | BGIBMGA001841 | IPR002018 | Carboxylesterase, |
| contig01200 | BGIBMGA004870 | IPR002018 | Carboxylesterase, |
| contig01200 | BGIBMGA007546 | IPR002018 | Carboxylesterase, |
| contig01200 | BGIBMGA010505 | IPR002290 | Serine/threonine |
| contig01200 | BGIBMGA007547 | IPR002018 | Carboxylesterase, |
| contig01200 | BGIBMGA012728 | IPR002018 | Carboxylesterase, |
| contig01200 | BGIBMGA004205 | IPR002018 | Carboxylesterase, |
| contig01200 | BGIBMGA004147 | IPR002168 | Lipolytic |
| contig01200 | BGIBMGA012729 | IPR002018 | Carboxylesterase, |
| contig01200 | BGIBMGA007545 | IPR002018 | Carboxylesterase, |
| contig01200 | BGIBMGA004684 | IPR002018 | Carboxylesterase, |
| contig01200 | BGIBMGA011218 | IPR002018 | Carboxylesterase, |
| contig01200 | BGIBMGA004683 | IPR002018 | Carboxylesterase, |
| contig01200 | BGIBMGA006081 | IPR002018 | Carboxylesterase, |
| contig01200 | BGIBMGA012031 | IPR002018 | Carboxylesterase, |
| contig01200 | BGIBMGA012122 | IPR002018 | Carboxylesterase, |
| contig01200 | BGIBMGA004206 | IPR002018 | Carboxylesterase, |
| contig01200 | BGIBMGA010328 | IPR002018 | Carboxylesterase, |
| contig01200 | BGIBMGA010987 | IPR002018 | Carboxylesterase, |
| contig01200 | BGIBMGA014599 | IPR002018 | Carboxylesterase, |
| contig01200 | BGIBMGA010976 | IPR002018 | Carboxylesterase, |
| contig01200 | BGIBMGA010988 | IPR002018 | Carboxylesterase, |
| contig01200 | BGIBMGA009874 | IPR000886 | Endoplasmic |
| contig01205 | BGIBMGA009392 | IPR004119 | Protein |
| contig01213 | BGIBMGA013756 | IPR002509 | Polysaccharide |
| contig01213 | BGIBMGA013757 | IPR002509 | Polysaccharide |
| contig01213 | BGIBMGA013758 | IPR002509 | Polysaccharide |
| contig01213 | BGIBMGA002696 | IPR002509 | Polysaccharide |
| contig01213 | BGIBMGA006213 | IPR002557 | Chitin |
| contig01213 | BGIBMGA008988 | IPR002172 | Low |
| contig01213 | BGIBMGA006214 | IPR002172 | Low |
| contig01213 | BGIBMGA010573 | IPR002557 | Chitin |
| contig01214 | BGIBMGA000532 | IPR001487 | Bromodomain |
| contig01218 | BGIBMGA013930 | IPR000639 | Epoxide |
| contig01218 | BGIBMGA013994 | IPR012336 | Thioredoxin-like |
| contig01218 | BGIBMGA009211 | IPR000639 | Epoxide |
| contig01220 | BGIBMGA012550 | IPR001733 | Peptidase |
| contig01222 | BGIBMGA012788 | IPR001314 | Peptidase |
| contig01222 | BGIBMGA012787 | IPR001254 | Peptidase |
| contig01222 | BGIBMGA012777 | IPR009003 | Peptidase, |
| contig01232 | BGIBMGA012485 | IPR000192 | Aminotransferase, |
| contig01233 | BGIBMGA013244 | IPR001345 | Phosphoglycerate/bisphosphoglycerate |
| contig01234 | BGIBMGA012852 | IPR000608 | Ubiquitin-conjugating |
| contig01237 | BGIBMGA001043 | IPR001196 | Ribosomal |
| contig01238 | BGIBMGA003580 | IPR001254 | Peptidase |
| contig01239 | BGIBMGA001136 | IPR007653 | Signal |
| contig01247 | BGIBMGA006066 | IPR006047 | Glycosyl |
| contig01248 | BGIBMGA003196 | IPR002843 | ATPase, |
| contig01258 | BGIBMGA005769 | IPR008856 | Translocon-associated |
| contig01262 | BGIBMGA003923 | IPR001976 | Ribosomal |
| contig01267 | BGIBMGA003726 | IPR001380 | Ribosomal |
| contig01268 | BGIBMGA002330 | IPR011078 | Protein |
| contig01270 | BGIBMGA011282 | IPR012606 | Ribosomal |
| contig01272 | BGIBMGA007516 | IPR009001 | EF-Tu/eEF-1alpha/eIF2-gamma, |
| contig01272 | BGIBMGA003608 | IPR000795 | Protein |
| contig01274 | BGIBMGA008348 | IPR000731 | Sterol-sensing |
| contig01274 | BGIBMGA009049 | IPR004765 | Niemann-Pick |
| contig01275 | BGIBMGA007477 | IPR011047 | Quinonprotein |
| contig01275 | BGIBMGA007459 | IPR005935 | Diphosphomevalonate |
| contig01276 | BGIBMGA005519 | IPR003591 | Leucine-rich |
| contig01277 | BGIBMGA012152 | IPR001395 | Aldo/keto |
| contig01277 | BGIBMGA014453 | IPR001395 | Aldo/keto |
| contig01279 | BGIBMGA010057 | IPR011211 | Tumor |
| contig01282 | BGIBMGA002405 | IPR001971 | Ribosomal |
| contig01284 | BGIBMGA000244 | IPR000061 | SWAP/Surp, |
| contig01287 | BGIBMGA000292 | IPR013027 | FAD-dependent |
| contig01290 | BGIBMGA001508 | IPR000734 | Lipase, |
| contig01290 | BGIBMGA001507 | IPR000734 | Lipase, |
| contig01290 | BGIBMGA001506 | IPR013818 | Lipase, |
| contig01290 | BGIBMGA002669 | IPR000734 | Lipase, |
| contig01290 | BGIBMGA002670 | IPR000734 | Lipase, |
| contig01290 | BGIBMGA010400 | IPR000734 | Lipase, |
| contig01293 | BGIBMGA005550 | IPR000164 | Histone |
| contig01293 | BGIBMGA009648 | IPR007125 | Histone |
| contig01293 | BGIBMGA014584 | IPR007125 | Histone |
| contig01295 | BGIBMGA003337 | IPR002143 | Ribosomal |
| contig01297 | BGIBMGA001842 | undefined |  |
| contig01301 | BGIBMGA000228 | undefined |  |
| contig01303 | BGIBMGA003028 | IPR000426 | Proteasome |
| contig01309 | BGIBMGA004647 | IPR000904 | SEC7-like |
| contig01311 | BGIBMGA013792 | IPR000266 | Ribosomal |
| contig01315 | BGIBMGA004068 | IPR005055 | Insect |
| contig01327 | BGIBMGA013945 | IPR004001 | Actin, |
| contig01327 | BGIBMGA009821 | IPR004000 | Actin/actin-like, |
| contig01327 | BGIBMGA005576 | IPR004000 | Actin/actin-like, |
| contig01327 | BGIBMGA005577 | IPR004000 | Actin/actin-like, |
| contig01327 | BGIBMGA012151 | IPR004001 | Actin, |
| contig01327 | BGIBMGA000082 | IPR004000 | Actin/actin-like |
| contig01329 | BGIBMGA011467 | IPR002670 | Ribosomal |
| contig01330 | BGIBMGA013511 | IPR006808 | ATPase, |
| contig01333 | BGIBMGA003943 | IPR001128 | Cytochrome |
| contig01333 | BGIBMGA003944 | IPR001128 | Cytochrome |
| contig01333 | BGIBMGA003945 | IPR001128 | Cytochrome |
| contig01333 | BGIBMGA003957 | IPR001128 | Cytochrome |
| contig01333 | BGIBMGA003926 | IPR001128 | Cytochrome |
| contig01333 | BGIBMGA002171 | IPR001128 | Cytochrome |
| contig01333 | BGIBMGA012386 | IPR001128 | Cytochrome |
| contig01333 | BGIBMGA012385 | IPR001128 | Cytochrome |
| contig01333 | BGIBMGA013241 | IPR001128 | Cytochrome |
| contig01333 | BGIBMGA001419 | IPR001128 | Cytochrome |
| contig01333 | BGIBMGA013238 | IPR002401 | E-class |
| contig01333 | BGIBMGA012089 | IPR001128 | Cytochrome |
| contig01334 | BGIBMGA003945 | IPR001128 | Cytochrome |
| contig01334 | BGIBMGA003957 | IPR001128 | Cytochrome |
| contig01335 | BGIBMGA008027 | IPR000175 | Sodium:neurotransmitter |
| contig01335 | BGIBMGA000927 | IPR000175 | Sodium:neurotransmitter |
| contig01335 | BGIBMGA004570 | IPR000175 | Sodium:neurotransmitter |
| contig01335 | BGIBMGA006619 | IPR000175 | Sodium:neurotransmitter |
| contig01335 | BGIBMGA006164 | IPR000175 | Sodium:neurotransmitter |
| contig01335 | BGIBMGA004312 | IPR000433 | Zinc |
| contig01335 | BGIBMGA007228 | IPR000175 | Sodium:neurotransmitter |
| contig01335 | BGIBMGA006857 | IPR000175 | Sodium:neurotransmitter |
| contig01335 | BGIBMGA014231 | IPR000175 | Sodium:neurotransmitter |
| contig01338 | BGIBMGA004827 | undefined |  |
| contig01339 | BGIBMGA001477 | undefined |  |
| contig01340 | BGIBMGA006937 | IPR002048 | Calcium-binding |
| contig01340 | BGIBMGA006742 | IPR002048 | Calcium-binding |
| contig01344 | BGIBMGA013114 | IPR000073 | Alpha/beta |
| contig01345 | BGIBMGA008242 | IPR001254 | Peptidase |
| contig01345 | BGIBMGA008280 | IPR001254 | Peptidase |
| contig01345 | BGIBMGA008281 | IPR001254 | Peptidase |
| contig01345 | BGIBMGA008279 | IPR001254 | Peptidase |
| contig01346 | BGIBMGA007517 | IPR000583 | Glutamine |
| contig01353 | BGIBMGA009103 | IPR002155 | Thiolase, |
| contig01355 | BGIBMGA001008 | IPR007114 | Major |
| contig01359 | BGIBMGA009411 | IPR000754 | Ribosomal |
| contig01360 | BGIBMGA009411 | IPR000754 | Ribosomal |
| contig01361 | BGIBMGA008875 | IPR000120 | Amidase |
| contig01367 | BGIBMGA013458 | IPR010591 | ATP11 |
| contig01368 | BGIBMGA005673 | IPR006693 | AB-hydrolase |
| contig01368 | BGIBMGA011777 | IPR008262 | Lipase, |
| contig01368 | BGIBMGA010640 | IPR008262 | Lipase, |
| contig01368 | BGIBMGA010639 | IPR008262 | Lipase, |
| contig01383 | BGIBMGA007547 | IPR002018 | Carboxylesterase, |
| contig01383 | BGIBMGA007546 | IPR002018 | Carboxylesterase, |
| contig01383 | BGIBMGA010505 | IPR002290 | Serine/threonine |
| contig01383 | BGIBMGA007545 | IPR002018 | Carboxylesterase, |
| contig01386 | BGIBMGA014136 | IPR013143 | PCI/PINT |
| contig01395 | BGIBMGA010738 | IPR002347 | Glucose/ribitol |
| contig01395 | BGIBMGA012574 | IPR002198 | Short-chain |
| contig01395 | BGIBMGA012527 | IPR002198 | Short-chain |
| contig01395 | BGIBMGA005208 | IPR002198 | Short-chain |
| contig01396 | BGIBMGA013567 | IPR000597 | Ribosomal |
| contig01404 | BGIBMGA007984 | IPR001020 | Phosphotransferase |
| contig01405 | BGIBMGA003517 | IPR002018 | Carboxylesterase, |
| contig01405 | BGIBMGA000837 | IPR002018 | Carboxylesterase, |
| contig01405 | BGIBMGA002796 | IPR002018 | Carboxylesterase, |
| contig01405 | BGIBMGA002902 | IPR002018 | Carboxylesterase, |
| contig01405 | BGIBMGA002901 | IPR002018 | Carboxylesterase, |
| contig01405 | BGIBMGA008141 | IPR002018 | Carboxylesterase, |
| contig01405 | BGIBMGA007670 | IPR002018 | Carboxylesterase, |
| contig01405 | BGIBMGA001629 | IPR002018 | Carboxylesterase, |
| contig01405 | BGIBMGA001630 | IPR002018 | Carboxylesterase, |
| contig01405 | BGIBMGA007671 | IPR008262 | Lipase, |
| contig01405 | BGIBMGA000875 | IPR002018 | Carboxylesterase, |
| contig01405 | BGIBMGA002899 | IPR002168 | Lipolytic |
| contig01405 | BGIBMGA012729 | IPR002018 | Carboxylesterase, |
| contig01405 | BGIBMGA004229 | IPR002018 | Carboxylesterase, |
| contig01405 | BGIBMGA012728 | IPR002018 | Carboxylesterase, |
| contig01405 | BGIBMGA012722 | IPR002018 | Carboxylesterase, |
| contig01405 | BGIBMGA004205 | IPR002018 | Carboxylesterase, |
| contig01405 | BGIBMGA011218 | IPR002018 | Carboxylesterase, |
| contig01405 | BGIBMGA010987 | IPR002018 | Carboxylesterase, |
| contig01405 | BGIBMGA004684 | IPR002018 | Carboxylesterase, |
| contig01405 | BGIBMGA004147 | IPR002168 | Lipolytic |
| contig01405 | BGIBMGA004683 | IPR002018 | Carboxylesterase, |
| contig01405 | BGIBMGA010988 | IPR002018 | Carboxylesterase, |
| contig01405 | BGIBMGA007547 | IPR002018 | Carboxylesterase, |
| contig01405 | BGIBMGA004870 | IPR002018 | Carboxylesterase, |
| contig01405 | BGIBMGA004934 | IPR002018 | Carboxylesterase, |
| contig01405 | BGIBMGA004206 | IPR002018 | Carboxylesterase, |
| contig01405 | BGIBMGA002698 | IPR002018 | Carboxylesterase, |
| contig01405 | BGIBMGA005195 | IPR002110 | Ankyrin, |
| contig01405 | BGIBMGA014599 | IPR002018 | Carboxylesterase, |
| contig01405 | BGIBMGA012122 | IPR002018 | Carboxylesterase, |
| contig01405 | BGIBMGA010976 | IPR002018 | Carboxylesterase, |
| contig01405 | BGIBMGA007546 | IPR002018 | Carboxylesterase, |
| contig01405 | BGIBMGA010328 | IPR002018 | Carboxylesterase, |
| contig01405 | BGIBMGA001964 | IPR000886 | Endoplasmic |
| contig01405 | BGIBMGA006081 | IPR002018 | Carboxylesterase, |
| contig01405 | BGIBMGA000776 | IPR002018 | Carboxylesterase, |
| contig01405 | BGIBMGA000777 | IPR002018 | Carboxylesterase, |
| contig01405 | BGIBMGA000774 | IPR002018 | Carboxylesterase, |
| contig01405 | BGIBMGA007545 | IPR002018 | Carboxylesterase, |
| contig01405 | BGIBMGA012585 | IPR002018 | Carboxylesterase, |
| contig01405 | BGIBMGA001841 | IPR002018 | Carboxylesterase, |
| contig01405 | BGIBMGA010505 | IPR002290 | Serine/threonine |
| contig01405 | BGIBMGA011893 | IPR002018 | Carboxylesterase, |
| contig01405 | BGIBMGA000772 | IPR002018 | Carboxylesterase, |
| contig01405 | BGIBMGA009874 | IPR000886 | Endoplasmic |
| contig01405 | BGIBMGA009544 | IPR002018 | Carboxylesterase, |
| contig01410 | BGIBMGA006867 | IPR000630 | Ribosomal |
| contig01411 | BGIBMGA003165 | undefined |  |
| contig01412 | BGIBMGA005696 | IPR013148 | Glycosyl |
| contig01413 | BGIBMGA000476 | undefined |  |
| contig01415 | BGIBMGA001507 | IPR000734 | Lipase, |
| contig01415 | BGIBMGA002670 | IPR000734 | Lipase, |
| contig01415 | BGIBMGA002669 | IPR000734 | Lipase, |
| contig01415 | BGIBMGA001508 | IPR000734 | Lipase, |
| contig01415 | BGIBMGA001506 | IPR013818 | Lipase, |
| contig01420 | BGIBMGA010291 | IPR006146 | 5'-Nucleotidase, |
| contig01420 | BGIBMGA010290 | IPR006146 | 5'-Nucleotidase, |
| contig01427 | BGIBMGA009106 | IPR010987 | Glutathione |
| contig01427 | BGIBMGA009107 | IPR012335 | Thioredoxin |
| contig01433 | BGIBMGA007900 | IPR002557 | Chitin |
| contig01438 | BGIBMGA007278 | IPR011701 | Major |
| contig01438 | BGIBMGA007281 | IPR011701 | Major |
| contig01444 | BGIBMGA001173 | IPR001604 | DNA/RNA |
| contig01444 | BGIBMGA009447 | IPR003006 | Immunoglobulin/major |
| contig01450 | BGIBMGA004165 | IPR000795 | Protein |
| contig01455 | BGIBMGA003805 | IPR008914 | PEBP, |
| contig01456 | BGIBMGA011581 | IPR000626 | Ubiquitin |
| contig01458 | BGIBMGA012527 | IPR002198 | Short-chain |
| contig01458 | BGIBMGA005208 | IPR002198 | Short-chain |
| contig01458 | BGIBMGA000813 | IPR002198 | Short-chain |
| contig01458 | BGIBMGA012375 | IPR002347 | Glucose/ribitol |
| contig01458 | BGIBMGA012574 | IPR002198 | Short-chain |
| contig01474 | BGIBMGA007743 | IPR008991 | Translation |
| contig01477 | BGIBMGA014022 | IPR001254 | Peptidase |
| contig01477 | BGIBMGA008514 | IPR001254 | Peptidase |
| contig01477 | BGIBMGA007377 | IPR009003 | Peptidase, |
| contig01477 | BGIBMGA014428 | IPR001254 | Peptidase |
| contig01477 | BGIBMGA014429 | IPR001254 | Peptidase |
| contig01477 | BGIBMGA014524 | IPR009003 | Peptidase, |
| contig01477 | BGIBMGA014431 | IPR001254 | Peptidase |
| contig01477 | BGIBMGA014430 | IPR001254 | Peptidase |
| contig01479 | BGIBMGA004515 | IPR002068 | Heat |
| contig01479 | BGIBMGA004541 | IPR001436 | Alpha |
| contig01479 | BGIBMGA004540 | IPR001436 | Alpha |
| contig01479 | BGIBMGA004630 | IPR001436 | Alpha |
| contig01479 | BGIBMGA004606 | IPR001436 | Alpha |
| contig01479 | BGIBMGA004103 | IPR001436 | Alpha |
| contig01480 | BGIBMGA006507 | IPR006095 | Glu/Leu/Phe/Val |
| contig01483 | BGIBMGA011712 | IPR011697 | Peptidase |
| contig01488 | BGIBMGA003567 | IPR001254 | Peptidase |
| contig01488 | BGIBMGA003568 | IPR001254 | Peptidase |
| contig01488 | BGIBMGA003580 | IPR001254 | Peptidase |
| contig01488 | BGIBMGA003566 | IPR001254 | Peptidase |
| contig01488 | BGIBMGA003569 | IPR001254 | Peptidase |
| contig01488 | BGIBMGA010590 | IPR001314 | Peptidase |
| contig01488 | BGIBMGA010061 | IPR001254 | Peptidase |
| contig01492 | BGIBMGA008616 | IPR010285 | Protein |
| contig01497 | BGIBMGA005270 | IPR004728 | Translocation |
| contig01504 | BGIBMGA001162 | IPR002401 | E-class |
| contig01506 | BGIBMGA012626 | IPR001912 | Ribosomal |
| contig01509 | BGIBMGA005994 | IPR003639 | Mov34-1, |
| contig01510 | BGIBMGA006709 | IPR006662 | Thioredoxin-related, |
| contig01511 | BGIBMGA005346 | IPR001128 | Cytochrome |
| contig01512 | BGIBMGA002811 | IPR001197 | Ribosomal |
| contig01514 | BGIBMGA006497 | IPR012493 | Renin |
| contig01518 | BGIBMGA001853 | IPR005294 | ATPase, |
| contig01519 | BGIBMGA002217 | IPR000198 | RhoGAP, |
| contig01520 | BGIBMGA001340 | undefined |  |
| contig01521 | BGIBMGA008525 | IPR012336 | Thioredoxin-like |
| contig01531 | BGIBMGA008670 | IPR001063 | Ribosomal |
| contig01533 | BGIBMGA007879 | IPR005485 | Eukaryotic |
| contig01534 | BGIBMGA009395 | IPR001680 | WD-40 |
| contig01540 | BGIBMGA006508 | IPR000348 | emp24/gp25L/p24, |
| contig01545 | BGIBMGA001525 | IPR008994 | Nucleic |
| contig01547 | BGIBMGA012772 | IPR011545 | DEAD/DEAH |
| contig01547 | BGIBMGA010673 | IPR014001 | DEAD-like |
| contig01547 | BGIBMGA004822 | IPR014014 | DEAD-box |
| contig01547 | BGIBMGA003186 | IPR014014 | DEAD-box |
| contig01548 | BGIBMGA008709 | IPR001579 | Glycoside |
| contig01551 | BGIBMGA003788 | IPR000873 | AMP-dependent |
| contig01556 | BGIBMGA007956 | IPR000990 | Innexin |
| contig01556 | BGIBMGA000452 | IPR000990 | Innexin |
| contig01556 | BGIBMGA004189 | IPR000990 | Innexin |
| contig01559 | BGIBMGA005995 | IPR000299 | Band |
| contig01560 | BGIBMGA004800 | IPR000834 | Peptidase |
| contig01560 | BGIBMGA004801 | IPR000834 | Peptidase |
| contig01564 | BGIBMGA009671 | IPR000868 | Isochorismatase |
| contig01574 | BGIBMGA007702 | IPR010982 | Lambda |
| contig01575 | BGIBMGA009010 | IPR000555 | Mov34/MPN/PAD-1, |
| contig01576 | BGIBMGA011544 | IPR011009 | Protein |
| contig01578 | BGIBMGA008514 | IPR001254 | Peptidase |
| contig01578 | BGIBMGA008515 | IPR009003 | Peptidase, |
| contig01578 | BGIBMGA014022 | IPR001254 | Peptidase |
| contig01578 | BGIBMGA007377 | IPR009003 | Peptidase, |
| contig01578 | BGIBMGA008513 | IPR001254 | Peptidase |
| contig01578 | BGIBMGA009526 | IPR001254 | Peptidase |
| contig01578 | BGIBMGA010584 | IPR001254 | Peptidase |
| contig01582 | BGIBMGA004661 | IPR010920 | Like-Sm |
| contig01583 | BGIBMGA000399 | IPR005812 | Ribosomal |
| contig01590 | BGIBMGA007245 | undefined |  |
| contig01593 | BGIBMGA007733 | undefined |  |
| contig01594 | BGIBMGA004162 | undefined |  |
| contig01598 | BGIBMGA011090 | IPR011701 | Major |
| contig01598 | BGIBMGA011016 | IPR011038 | Calycin-like, |
| contig01601 | BGIBMGA003833 | IPR002478 | PUA, |
| contig01609 | BGIBMGA009953 | IPR000215 | Proteinase |
| contig01609 | BGIBMGA007720 | IPR000215 | Proteinase |
| contig01611 | BGIBMGA008001 | IPR004148 | BAR, |
| contig01621 | BGIBMGA011273 | IPR007087 | Zinc |
| contig01624 | BGIBMGA005560 | IPR003903 | Ubiquitin |
| contig01625 | BGIBMGA012029 | IPR005746 | Thioredoxin, |
| contig01628 | BGIBMGA000701 | IPR011614 | Catalase, |
| contig01628 | BGIBMGA012691 | IPR002226 | Catalase, |
| contig01628 | BGIBMGA014510 | IPR002226 | Catalase, |
| contig01628 | BGIBMGA011431 | IPR002226 | Catalase, |
| contig01628 | BGIBMGA011430 | IPR002226 | Catalase, |
| contig01628 | BGIBMGA014511 | IPR002226 | Catalase, |
| contig01631 | BGIBMGA004612 | IPR009079 | Four-helical |
| contig01640 | BGIBMGA007710 | IPR005716 | Ribosomal |
| contig01642 | BGIBMGA004728 | IPR000215 | Proteinase |
| contig01645 | BGIBMGA012134 | IPR008974 | TRAF-like, |
| contig01652 | BGIBMGA008959 | undefined |  |
| contig01656 | BGIBMGA011687 | IPR005062 | SAC3/GANP/Nin1/mts3/eIF-3 |
| contig01664 | BGIBMGA010876 | IPR004943 | Lepidopteran |
| contig01664 | BGIBMGA010168 | IPR004943 | Lepidopteran |
| contig01664 | BGIBMGA009621 | undefined |  |
| contig01664 | BGIBMGA009573 | IPR004943 | Lepidopteran |
| contig01664 | BGIBMGA008165 | IPR004943 | Lepidopteran |
| contig01664 | BGIBMGA010877 | IPR004943 | Lepidopteran |
| contig01664 | BGIBMGA008164 | IPR004943 | Lepidopteran |
| contig01664 | BGIBMGA004464 | IPR004943 | Lepidopteran |
| contig01664 | BGIBMGA004463 | IPR004943 | Lepidopteran |
| contig01667 | BGIBMGA008815 | IPR002048 | Calcium-binding |
| contig01667 | BGIBMGA006444 | IPR002048 | Calcium-binding |
| contig01667 | BGIBMGA008814 | IPR002048 | Calcium-binding |
| contig01667 | BGIBMGA008813 | IPR002048 | Calcium-binding |
| contig01669 | BGIBMGA006083 | IPR002198 | Short-chain |
| contig01669 | BGIBMGA002979 | IPR002198 | Short-chain |
| contig01669 | BGIBMGA013364 | IPR002347 | Glucose/ribitol |
| contig01669 | BGIBMGA014138 | IPR002347 | Glucose/ribitol |
| contig01678 | BGIBMGA001347 | IPR009911 | Fibroin |
| contig01683 | BGIBMGA009130 | IPR005729 | Ribosomal |
| contig01690 | BGIBMGA004200 | IPR006693 | AB-hydrolase |
| contig01693 | BGIBMGA011819 | IPR005442 | Glutathione |
| contig01695 | BGIBMGA003522 | IPR002130 | Peptidyl-prolyl |
| contig01697 | BGIBMGA007950 | IPR000886 | Endoplasmic |
| contig01697 | BGIBMGA002381 | IPR001023 | Heat |
| contig01697 | BGIBMGA004614 | IPR001023 | Heat |
| contig01697 | BGIBMGA014536 | IPR013126 | Heat |
| contig01697 | BGIBMGA001635 | IPR001023 | Heat |
| contig01697 | BGIBMGA006313 | IPR001023 | Heat |
| contig01697 | BGIBMGA014618 | IPR013126 | Heat |
| contig01700 | BGIBMGA010471 | IPR001715 | Calponin-like |
| contig01701 | BGIBMGA014087 | IPR013740 | Redoxin, |
| contig01703 | BGIBMGA011936 | IPR008689 | ATPase, |
| contig01710 | BGIBMGA000897 | IPR001509 | NAD-dependent |
| contig01716 | BGIBMGA011209 | IPR011032 | GroES-like, |
| contig01717 | BGIBMGA007739 | IPR008978 | HSP20-like |
| contig01722 | BGIBMGA010227 | IPR003034 | DNA-binding |
| contig01723 | BGIBMGA006141 | IPR003639 | Mov34-1, |
| contig01725 | BGIBMGA003833 | IPR002478 | PUA, |
| contig01726 | BGIBMGA011074 | IPR004911 | Gamma |
| contig01730 | BGIBMGA011504 | IPR000711 | ATPase, |
| contig01735 | BGIBMGA009052 | IPR006140 | D-isomer |
| contig01737 | BGIBMGA005462 | IPR008011 | Complex |
| contig01743 | BGIBMGA009698 | IPR002715 | Nascent |
| contig01745 | BGIBMGA007453 | IPR001189 | Manganese |
| contig01749 | BGIBMGA003669 | IPR000717 | Proteasome |
| contig01752 | BGIBMGA000971 | IPR008012 | Proteasome |
| contig01758 | BGIBMGA007889 | IPR011046 | WD40-like, |
| contig01761 | BGIBMGA008595 | IPR006746 | 26S |
| contig01781 | BGIBMGA009088 | undefined |  |
| contig01785 | BGIBMGA010437 | IPR006599 | Adenylate |
| contig01787 | BGIBMGA011323 | undefined |  |
| contig01795 | BGIBMGA009814 | IPR005834 | Haloacid |
| contig01796 | BGIBMGA010471 | IPR001715 | Calponin-like |
| contig01799 | BGIBMGA006835 | IPR005633 | Ribosomal |
| contig01800 | BGIBMGA009476 | IPR009020 | Proteinase |
| contig01803 | BGIBMGA004801 | IPR000834 | Peptidase |
| contig01803 | BGIBMGA004800 | IPR000834 | Peptidase |
| contig01803 | BGIBMGA004798 | IPR000834 | Peptidase |
| contig01803 | BGIBMGA006715 | IPR000834 | Peptidase |
| contig01803 | BGIBMGA004797 | IPR000834 | Peptidase |
| contig01803 | BGIBMGA008910 | IPR003146 | Proteinase |
| contig01803 | BGIBMGA008976 | IPR009020 | Proteinase |
| contig01803 | BGIBMGA009486 | IPR009020 | Proteinase |
| contig01803 | BGIBMGA006871 | IPR003146 | Proteinase |
| contig01803 | BGIBMGA009478 | IPR009020 | Proteinase |
| contig01803 | BGIBMGA004830 | IPR000834 | Peptidase |
| contig01803 | BGIBMGA004799 | IPR000834 | Peptidase |
| contig01803 | BGIBMGA009487 | IPR009020 | Proteinase |
| contig01803 | BGIBMGA009477 | IPR009020 | Proteinase |
| contig01807 | BGIBMGA006819 | IPR004806 | UV |
| contig01835 | BGIBMGA011186 | IPR001683 | Phox-like |
| contig01837 | BGIBMGA007889 | IPR011046 | WD40-like, |
| contig01839 | BGIBMGA013449 | IPR002119 | Histone |
| contig01841 | BGIBMGA010621 | IPR013346 | Ribonucleoside-diphosphate |
| contig01844 | BGIBMGA009863 | IPR006646 | KOW |
| contig01853 | BGIBMGA004612 | IPR009079 | Four-helical |
| contig01853 | BGIBMGA012753 | IPR001404 | Heat |
| contig01855 | BGIBMGA004356 | IPR000554 | Ribosomal |
| contig01859 | BGIBMGA012961 | IPR011992 | EF-Hand |
| contig01865 | BGIBMGA004546 | IPR007110 | Immunoglobulin-like, |
| contig01868 | BGIBMGA004176 | IPR000648 | Oxysterol-binding |
| contig01871 | BGIBMGA002940 | IPR002198 | Short-chain |
| contig01871 | BGIBMGA002941 | IPR002198 | Short-chain |
| contig01871 | BGIBMGA002944 | IPR002198 | Short-chain |
| contig01871 | BGIBMGA002942 | IPR002198 | Short-chain |
| contig01873 | BGIBMGA002309 | IPR004301 | Nucleoplasmin |
| contig01876 | BGIBMGA013930 | IPR000639 | Epoxide |
| contig01879 | BGIBMGA005727 | IPR004455 | NADP |
| contig01880 | BGIBMGA007935 | IPR000031 | 1-(5-Phosphoribosyl)-5-amino-4-imidazole-carboxylate |
| contig01882 | BGIBMGA002919 | IPR000301 | CD9/CD37/CD63 |
| contig01885 | BGIBMGA011756 | undefined |  |
| contig01887 | BGIBMGA000336 | IPR000618 | Insect |
| contig01892 | BGIBMGA009559 | IPR007305 | Got1-like |
| contig01896 | BGIBMGA003562 | IPR006806 | ETC |
| contig01899 | BGIBMGA005928 | IPR001854 | Ribosomal |
| contig01902 | BGIBMGA001964 | IPR000886 | Endoplasmic |
| contig01902 | BGIBMGA001963 | IPR000886 | Endoplasmic |
| contig01906 | BGIBMGA008514 | IPR001254 | Peptidase |
| contig01906 | BGIBMGA008515 | IPR009003 | Peptidase, |
| contig01906 | BGIBMGA014022 | IPR001254 | Peptidase |
| contig01906 | BGIBMGA007377 | IPR009003 | Peptidase, |
| contig01906 | BGIBMGA009526 | IPR001254 | Peptidase |
| contig01906 | BGIBMGA010584 | IPR001254 | Peptidase |
| contig01911 | BGIBMGA002493 | IPR000994 | Peptidase |
| contig01915 | BGIBMGA001395 | IPR001353 | 20S |
| contig01926 | BGIBMGA002644 | IPR000308 | 14-3-3 |
| contig01926 | BGIBMGA013201 | IPR000308 | 14-3-3 |
| contig01929 | BGIBMGA002594 | IPR000850 | Adenylate |
| contig01938 | BGIBMGA006066 | IPR006047 | Glycosyl |
| contig01938 | BGIBMGA003057 | IPR006589 | Glycosyl |
| contig01938 | BGIBMGA003056 | IPR006047 | Glycosyl |
| contig01939 | BGIBMGA006981 | IPR008568 | Protein |
| contig01945 | BGIBMGA005494 | undefined |  |
| contig01956 | BGIBMGA007637 | IPR004154 | Anticodon-binding, |
| contig01960 | BGIBMGA004798 | IPR000834 | Peptidase |
| contig01960 | BGIBMGA004797 | IPR000834 | Peptidase |
| contig01965 | BGIBMGA001943 | IPR003348 | Anion-transporting |
| contig01968 | BGIBMGA005439 | IPR006138 | NADH |
| contig01968 | BGIBMGA005137 | IPR006138 | NADH |
| contig01976 | BGIBMGA006523 | IPR012913 | Glucosidase |
| contig01979 | BGIBMGA007349 | IPR008950 | GroEL-like |
| contig01990 | BGIBMGA003186 | IPR014014 | DEAD-box |
| contig01990 | BGIBMGA004822 | IPR014014 | DEAD-box |
| contig01992 | BGIBMGA011029 | IPR002155 | Thiolase |
| contig02006 | BGIBMGA000959 | IPR000054 | Ribosomal |
| contig02010 | BGIBMGA014059 | IPR002624 | Deoxynucleoside |
| contig02013 | BGIBMGA010671 | IPR009374 | Eukaryotic |
| contig02015 | BGIBMGA006541 | IPR000504 | RNA-binding |
| contig02035 | BGIBMGA013782 | IPR000437 | Prokaryotic |
| contig02048 | BGIBMGA011578 | IPR001441 | Di-trans-poly-cis-decaprenylcistransferase |
| contig02057 | BGIBMGA004009 | IPR006885 | ETC |
| contig02058 | BGIBMGA012690 | IPR012948 | AARP2CN, |
| contig02066 | BGIBMGA012936 | IPR007087 | Zinc |
| contig02071 | BGIBMGA008516 | IPR002717 | MOZ/SAS-like |
| contig02071 | BGIBMGA006995 | IPR002717 | MOZ/SAS-like |
| contig02082 | BGIBMGA005930 | IPR011046 | WD40-like, |
| contig02083 | BGIBMGA003048 | IPR009582 | Microsomal |
| contig02092 | BGIBMGA001549 | IPR000626 | Ubiquitin |
| contig02092 | BGIBMGA001415 | IPR000626 | Ubiquitin |
| contig02098 | BGIBMGA008459 | IPR008940 | Protein |
| contig02110 | BGIBMGA011131 | IPR001135 | NADH-ubiquinone |
| contig02116 | BGIBMGA009319 | IPR009019 | KH, |
| contig02124 | BGIBMGA005480 | IPR004687 | Golgi |
| contig02137 | BGIBMGA005576 | IPR004000 | Actin/actin-like, |
| contig02137 | BGIBMGA005577 | IPR004000 | Actin/actin-like, |
| contig02137 | BGIBMGA013945 | IPR004001 | Actin, |
| contig02145 | BGIBMGA008875 | IPR000120 | Amidase |
| contig02149 | BGIBMGA008921 | IPR001326 | Elongation |
| contig02153 | BGIBMGA006426 | IPR013069 | BTB/POZ, |
| contig02154 | BGIBMGA003643 | IPR007116 | 6-pyruvoyl |
| contig02157 | BGIBMGA007676 | IPR001607 | Zinc |
| contig02158 | BGIBMGA010318 | IPR006596 | Nucleotide |
| contig02174 | BGIBMGA007412 | IPR008195 | Ribosomal |
| contig02175 | BGIBMGA010569 | IPR000086 | NUDIX |
| contig02176 | BGIBMGA010475 | IPR008940 | Protein |
| contig02177 | BGIBMGA002572 | IPR000196 | Ribosomal |
| contig02180 | BGIBMGA010959 | IPR009038 | GOLD, |
| contig02181 | BGIBMGA008856 | IPR000595 | Cyclic |
| contig02183 | BGIBMGA007723 | IPR000801 | Putative |
| contig02187 | BGIBMGA003018 | IPR007121 | RNA |
| contig02191 | BGIBMGA001022 | IPR008952 | Tetraspanin, |
| contig02200 | BGIBMGA007645 | IPR008994 | Nucleic |
| contig02202 | BGIBMGA007838 | IPR002123 | Phospholipid/glycerol |
| contig02203 | BGIBMGA001803 | IPR011356 | Peptidase |
| contig02216 | BGIBMGA014135 | IPR007087 | Zinc |
| contig02219 | BGIBMGA004847 | IPR001680 | WD-40 |
| contig02222 | BGIBMGA001470 | IPR000313 | PWWP |
| contig02226 | BGIBMGA003186 | IPR014014 | DEAD-box |
| contig02229 | BGIBMGA010537 | IPR001360 | Glycoside |
| contig02229 | BGIBMGA010536 | IPR001360 | Glycoside |
| contig02229 | BGIBMGA014178 | IPR001360 | Glycoside |
| contig02229 | BGIBMGA002660 | IPR001360 | Glycoside |
| contig02229 | BGIBMGA005602 | IPR001360 | Glycoside |
| contig02229 | BGIBMGA010812 | IPR001360 | Glycoside |
| contig02229 | BGIBMGA003512 | IPR001360 | Glycoside |
| contig02229 | BGIBMGA014191 | IPR001360 | Glycoside |
| contig02229 | BGIBMGA002450 | IPR001360 | Glycoside |
| contig02230 | BGIBMGA005035 | IPR008709 | Neurochondrin |
| contig02237 | BGIBMGA004982 | IPR001202 | WW/Rsp5/WWP |
| contig02241 | BGIBMGA005391 | IPR000659 | Pyridoxamine |
| contig02247 | BGIBMGA001119 | IPR002005 | Rab |
| contig02264 | BGIBMGA008189 | IPR001753 | Enoyl-CoA |
| contig02277 | BGIBMGA006161 | IPR001893 | Cysteine |
| contig02279 | BGIBMGA010664 | IPR000715 | Glycosyl |
| contig02283 | BGIBMGA013816 | IPR011009 | Protein |
| contig02289 | BGIBMGA010577 | IPR007051 | CHORD, |
| contig02297 | BGIBMGA011777 | IPR008262 | Lipase, |
| contig02297 | BGIBMGA010639 | IPR008262 | Lipase, |
| contig02297 | BGIBMGA010640 | IPR008262 | Lipase, |
| contig02298 | BGIBMGA013974 | IPR000449 | Ubiquitin-associated, |
| contig02302 | BGIBMGA010471 | IPR001715 | Calponin-like |
| contig02304 | BGIBMGA005064 | IPR001965 | Zinc |
| contig02306 | BGIBMGA002944 | IPR002198 | Short-chain |
| contig02309 | BGIBMGA008059 | IPR006025 | Peptidase |
| contig02310 | BGIBMGA000188 | IPR003038 | Defender |
| contig02311 | BGIBMGA002455 | undefined |  |
| contig02317 | BGIBMGA007645 | IPR008994 | Nucleic |
| contig02334 | BGIBMGA010536 | IPR001360 | Glycoside |
| contig02335 | BGIBMGA001498 | IPR005988 | Synaptic |
| contig02352 | BGIBMGA001232 | IPR001841 | Zinc |
| contig02355 | BGIBMGA014046 | IPR001128 | Cytochrome |
| contig02380 | BGIBMGA012672 | IPR007846 | MPPN |
| contig02389 | BGIBMGA001708 | undefined |  |
| contig02392 | BGIBMGA005312 | IPR012972 | NLE, |
| contig02402 | BGIBMGA006583 | IPR004360 | Glyoxalase/bleomycin |
| contig02431 | BGIBMGA009934 | IPR001648 | Ribosomal |
| contig02434 | BGIBMGA011604 | IPR000571 | Zinc |
| contig02437 | BGIBMGA002718 | IPR000705 | Galactokinase, |
| contig02442 | BGIBMGA006705 | IPR001440 | Tetratricopeptide |
| contig02460 | BGIBMGA002669 | IPR000734 | Lipase, |
| contig02460 | BGIBMGA002670 | IPR000734 | Lipase, |
| contig02463 | BGIBMGA004703 | IPR005225 | Small |
| contig02473 | BGIBMGA000800 | IPR011012 | Longin-like, |
| contig02479 | BGIBMGA004496 | IPR013026 | Tetratricopeptide |
| contig02481 | BGIBMGA010593 | IPR008914 | PEBP |
| contig02488 | BGIBMGA012089 | IPR001128 | Cytochrome |
| contig02488 | BGIBMGA012386 | IPR001128 | Cytochrome |
| contig02488 | BGIBMGA012385 | IPR001128 | Cytochrome |
| contig02496 | BGIBMGA005664 | IPR001661 | Glycoside |
| contig02503 | BGIBMGA000624 | IPR001452 | Src |
| contig02513 | BGIBMGA003978 | IPR000911 | Ribosomal |
| contig02521 | BGIBMGA006800 | undefined |  |
| contig02522 | BGIBMGA001206 | IPR002423 | Chaperonin |
| contig02524 | BGIBMGA006613 | IPR011047 | Quinonprotein |
| contig02525 | BGIBMGA010471 | IPR001715 | Calponin-like |
| contig02534 | BGIBMGA004612 | IPR009079 | Four-helical |
| contig02536 | BGIBMGA004612 | IPR009079 | Four-helical |
| contig02544 | BGIBMGA011508 | IPR002423 | Chaperonin |
| contig02546 | BGIBMGA014340 | IPR012486 | N1221-like |
| contig02548 | BGIBMGA008815 | IPR002048 | Calcium-binding |
| contig02576 | BGIBMGA012994 | IPR003010 | Nitrilase/cyanide |
| contig02585 | BGIBMGA008342 | IPR000594 | UBA/THIF-type |
| contig02586 | BGIBMGA013030 | IPR000222 | Protein |
| contig02592 | BGIBMGA000683 | IPR001715 | Calponin-like |
| contig02593 | BGIBMGA009262 | IPR002125 | CMP/dCMP |
| contig02594 | BGIBMGA002660 | IPR001360 | Glycoside |
| contig02606 | BGIBMGA013150 | IPR007268 | Rad9 |
| contig02641 | BGIBMGA010409 | IPR001715 | Calponin-like |
| contig02658 | BGIBMGA008210 | IPR001478 | PDZ/DHR/GLGF |
| contig02667 | BGIBMGA005146 | undefined |  |
| contig02686 | BGIBMGA000624 | IPR001452 | Src |
| contig02689 | BGIBMGA010200 | IPR001296 | Glycosyl |
| contig02699 | BGIBMGA006192 | IPR003957 | Histone-like |
| contig02721 | BGIBMGA014337 | IPR002319 | Phenylalanyl-tRNA |
| contig02724 | BGIBMGA007060 | IPR010418 | ECSIT |
| contig02746 | BGIBMGA005076 | IPR009316 | COG |
| contig02763 | BGIBMGA013829 | IPR002213 | UDP-glucuronosyl/UDP-glucosyltransferase |
| contig02763 | BGIBMGA013831 | IPR002213 | UDP-glucuronosyl/UDP-glucosyltransferase |
| contig02763 | BGIBMGA013860 | IPR002213 | UDP-glucuronosyl/UDP-glucosyltransferase |
| contig02763 | BGIBMGA013830 | IPR002213 | UDP-glucuronosyl/UDP-glucosyltransferase |
| contig02763 | BGIBMGA013859 | IPR002213 | UDP-glucuronosyl/UDP-glucosyltransferase |
| contig02763 | BGIBMGA013833 | IPR002213 | UDP-glucuronosyl/UDP-glucosyltransferase |
| contig02778 | BGIBMGA007795 | IPR004038 | Ribosomal |
| contig02780 | BGIBMGA007637 | IPR004154 | Anticodon-binding, |
| contig02783 | BGIBMGA008059 | IPR006025 | Peptidase |
| contig02818 | BGIBMGA004927 | IPR007018 | MED6 |
| contig02863 | BGIBMGA013998 | IPR002051 | Haem |
| contig02870 | BGIBMGA001415 | IPR000626 | Ubiquitin |
| contig02870 | BGIBMGA001549 | IPR000626 | Ubiquitin |
| contig02904 | BGIBMGA011272 | IPR001047 | Ribosomal |
| contig02956 | BGIBMGA001159 | IPR007261 | Vacuolar |
| contig02969 | BGIBMGA013756 | IPR002509 | Polysaccharide |
| contig02969 | BGIBMGA013757 | IPR002509 | Polysaccharide |
| contig02987 | BGIBMGA011190 | IPR011009 | Protein |
| contig02994 | BGIBMGA011746 | IPR000629 | ATP-dependent |
| contig02994 | BGIBMGA011965 | IPR000629 | ATP-dependent |
| contig02994 | BGIBMGA001336 | IPR014001 | DEAD-like |
| contig02994 | BGIBMGA011754 | IPR000629 | ATP-dependent |
| contig02994 | BGIBMGA012013 | IPR014001 | DEAD-like |
| contig03008 | BGIBMGA013923 | IPR005825 | Ribosomal |
| contig03071 | BGIBMGA000947 | undefined |  |
| contig03072 | BGIBMGA011898 | IPR001251 | Cellular |
| contig03079 | BGIBMGA000666 | IPR001251 | Cellular |
| contig03098 | BGIBMGA013893 | IPR001747 | Lipid |
| contig03126 | BGIBMGA008023 | IPR013766 | Thioredoxin |
| contig03137 | BGIBMGA005499 | IPR004240 | Nonaspanin |
| contig03166 | BGIBMGA011316 | undefined |  |
| contig03184 | BGIBMGA006237 | IPR001510 | Zinc |
| contig03300 | BGIBMGA006243 | IPR007330 | MIT, |
| contig03325 | BGIBMGA004870 | IPR002018 | Carboxylesterase, |
| contig03325 | BGIBMGA003073 | IPR001983 | Translationally |
| contig03325 | BGIBMGA007547 | IPR002018 | Carboxylesterase, |
| contig03325 | BGIBMGA010505 | IPR002290 | Serine/threonine |
| contig03325 | BGIBMGA007546 | IPR002018 | Carboxylesterase, |
| contig03325 | BGIBMGA007545 | IPR002018 | Carboxylesterase, |
| contig03325 | BGIBMGA012728 | IPR002018 | Carboxylesterase, |
| contig03325 | BGIBMGA012729 | IPR002018 | Carboxylesterase, |
| contig03325 | BGIBMGA004205 | IPR002018 | Carboxylesterase, |
| contig03325 | BGIBMGA006081 | IPR002018 | Carboxylesterase, |
| contig03325 | BGIBMGA002698 | IPR002018 | Carboxylesterase, |
| contig03325 | BGIBMGA004206 | IPR002018 | Carboxylesterase, |
| contig03325 | BGIBMGA012031 | IPR002018 | Carboxylesterase, |
| contig03325 | BGIBMGA004684 | IPR002018 | Carboxylesterase, |
| contig03325 | BGIBMGA004147 | IPR002168 | Lipolytic |
| contig03325 | BGIBMGA000777 | IPR002018 | Carboxylesterase, |
| contig03325 | BGIBMGA010987 | IPR002018 | Carboxylesterase, |
| contig03325 | BGIBMGA001841 | IPR002018 | Carboxylesterase, |
| contig03325 | BGIBMGA003517 | IPR002018 | Carboxylesterase, |
| contig03341 | BGIBMGA011029 | IPR002155 | Thiolase |
| contig03343 | BGIBMGA009953 | IPR000215 | Proteinase |
| contig03343 | BGIBMGA004728 | IPR000215 | Proteinase |
| contig03370 | BGIBMGA003397 | IPR001047 | Ribosomal |
| contig03379 | BGIBMGA005024 | IPR010629 | Insect |
| contig03379 | BGIBMGA005025 | IPR010629 | Insect |
| contig03383 | BGIBMGA010487 | IPR000552 | Ribosomal |
| contig03388 | BGIBMGA011895 | IPR013818 | Lipase, |
| contig03388 | BGIBMGA002669 | IPR000734 | Lipase, |
| contig03392 | BGIBMGA007795 | IPR004038 | Ribosomal |
| contig03393 | BGIBMGA011446 | IPR000626 | Ubiquitin, |
| contig03396 | BGIBMGA001991 | IPR000509 | Ribosomal |
| contig03398 | BGIBMGA009751 | IPR000218 | Ribosomal |
| contig03420 | BGIBMGA007915 | IPR006025 | Peptidase |
| contig03420 | BGIBMGA007916 | IPR006025 | Peptidase |
| contig03423 | BGIBMGA008050 | IPR001715 | Calponin-like |
| contig03423 | BGIBMGA007092 | IPR001715 | Calponin-like |
| contig03430 | BGIBMGA000867 | IPR005711 | Ribosomal |
| contig03431 | BGIBMGA001471 | IPR005713 | Ribosomal |
| contig03443 | BGIBMGA005024 | IPR010629 | Insect |
| contig03443 | BGIBMGA005025 | IPR010629 | Insect |
| contig03444 | BGIBMGA008011 | IPR005772 | ATPase, |
| contig03447 | BGIBMGA007363 | IPR008957 | Fibronectin, |
| contig03449 | BGIBMGA001106 | IPR008991 | Translation |
| contig03450 | BGIBMGA008903 | IPR002674 | Ribosomal |
| contig03479 | BGIBMGA008050 | IPR001715 | Calponin-like |
| contig03479 | BGIBMGA007092 | IPR001715 | Calponin-like |
| contig03490 | BGIBMGA000829 | IPR004871 | CPSF |
| contig03491 | BGIBMGA001483 | IPR001210 | Ribosomal |
| contig03494 | BGIBMGA011009 | undefined |  |
| contig03502 | BGIBMGA013923 | IPR005825 | Ribosomal |
| contig03512 | BGIBMGA007729 | IPR000215 | Proteinase |
| contig03516 | BGIBMGA006964 | IPR003439 | ABC |
| contig03541 | BGIBMGA009012 | IPR002327 | Cytochrome |
| contig03542 | BGIBMGA005240 | IPR013772 | Alpha-amylase, |
| contig03542 | BGIBMGA001876 | IPR006046 | Glycoside |
| contig03542 | BGIBMGA005239 | IPR013772 | Alpha-amylase, |
| contig03552 | BGIBMGA008709 | IPR001579 | Glycoside |
| contig03558 | BGIBMGA007228 | IPR000175 | Sodium:neurotransmitter |
| contig03566 | BGIBMGA007228 | IPR000175 | Sodium:neurotransmitter |
| contig03587 | BGIBMGA009953 | IPR000215 | Proteinase |
| contig03587 | BGIBMGA007720 | IPR000215 | Proteinase |
| contig03587 | BGIBMGA004726 | IPR000215 | Proteinase |
| contig03592 | BGIBMGA007879 | IPR005485 | Eukaryotic |
| contig03610 | BGIBMGA002670 | IPR000734 | Lipase, |
| contig03610 | BGIBMGA002669 | IPR000734 | Lipase, |
| contig03627 | BGIBMGA013792 | IPR000266 | Ribosomal |
| contig03628 | BGIBMGA004374 | IPR000530 | Ribosomal |
| contig03632 | BGIBMGA003475 | IPR001326 | Translation |
| contig03641 | BGIBMGA010684 | IPR003177 | Cytochrome |
| contig03658 | BGIBMGA004905 | IPR003959 | AAA |
| contig03659 | BGIBMGA009477 | IPR009020 | Proteinase |
| contig03659 | BGIBMGA009478 | IPR009020 | Proteinase |
| contig03679 | BGIBMGA004331 | IPR001179 | Peptidylprolyl |
| contig03690 | BGIBMGA010402 | IPR002086 | Aldehyde |
| contig03728 | BGIBMGA000387 | IPR001611 | Leucine-rich |
| contig00003 | BGIBMGA013930 | IPR000639 | Epoxide |
