# Supplementary material for: Transcriptome Sequencing, and Rapid Development and Application of SNP Markers for the Legume Pod Borer Maruca vitrata (Lepidoptera: Crambidae)
Source: PLoS One. 2011 Jul 6;6(7):e21388. doi: 10.1371/journal.pone.0021388 (PMC3130784; doi:10.1371/journal.pone.0021388)
Supplement: Data S2 — Derived amino acid sequence of Maruca vitrata EST contigs assigned putative functional annotation as alanine aminopeptidase (APN) encoding gene sequences. (DOC) [file pone.0021388.s002.doc]

**Supplemental Data S2:** Derived amino acid sequence of *Maruca vitrata* EST contigs assigned putative functional annotation as alanine aminopeptidase (APN) encoding gene sequences.

**A)** Contig00544

>contig00544 length=535 numreads=12 (Note: nucleotide deleted from coding frame are shaded), and nucleotides inserted are underlined).

TCGGAGAAtACCGCTGACATGGTcGTCATCCTTCGtGCCCTCGCTTGCACCAgggAC**a**gCCCTCTTGCAGacTTATttGGGTCAGACCCTGAGCAACGACAGGAtccGCGCCCACGACCGCACTAATGCCTTCTTGTACGccctcCAAgggaaccgggAGAGCCgggACATCGtCcTGGACTtCTTGTTcgacAACTTCGAAGCCATCAGGGAAGAGcAcGGTGgACAGGCCCGTCTCAACGTGAACATCAACAACTGCGCTGCATTCTTGACACAGTTCACACAGATCCAAAGGTTCCA**n**ACCTGGGCCTACGCCAACcAGAtAGCTTTGGGCAGTGGcaTTCTCCAGtGctGTGTCGGCCATCagcTCAGCTCAgtCcAACCTGGcTtGGGCCAACagcAaCGCCTGGCCATCcACACCACCCTCCTCGCctAGGgCTCcGCTGCCTCCATCgTaGCTCCCTTCGCCCTTATtgTGGCCGCTCTGGTCTCTCACCtccTaCGctAAGaTTTAGTCTACCGCGCaTACCATCGGC

>contig00544_AminoAcidTranslation

SENTADMVVILRALACTRDALLQTYLGQTLSNDRIRAHDRTNAFLYALQGNRESRDIVLDFLFDNFEAIREEHGGQARLNVNINNCAAFLTQFTQIQRFXTWAYANQIALGS

VIRTUAL RIBOSOME

>contig00544 length=535 Reading frame: 1 Translation table: Standard SGC0

S E N T A D M V V I L R A L A C T R D A L L Q T Y L G Q T L

5' TCGGAGAAtACCGCTGACATGGTcGTCATCCTTCGtGCCCTCGCTTGCACCAgggACgCCCTCTTGCAGacTTATttGGGTCAGACCCTG 90

..................>>>..........................................))).........))).........)))

S N D R I R A H D R T N A F L Y A L Q G N R E S R D I V L D

5' AGCAACGACAGGAtccGCGCCCACGACCGCACTAATGCCTTCTTGTACGccctcCAAgggaaccgggAGAGCCgggACATCGtCcTGGAC 180

..........................................))).......................................)))...

F L F D N F E A I R E E H G G Q A R L N V N I N N C A A F L

5' TtCTTGTTcgacAACTTCGAAGCCATCAGGGAAGAGcAcGGTGgACAGGCCCGTCTCAACGTGAACATCAACAACTGCGCTGCATTCTTG 270

...))).................................................................................)))

T Q F T Q I Q R F X T W A Y A N Q I A L G S

5' ACACAGTTCACACAGATCCAAAGGTTCCAnACCTGGGCCTACGCCAACcAGAtAGCTTTGGGCAGTGGcaTTCTCCAGtGctGTGTCGGC 360

.........................................................)))......

5' CATCagcTCAGCTCAgtCcAACCTGGcTtGGGCCAACagcAaCGCCTGGCCATCcACACCACCCTCCTCGCctAGGgCTCcGCTGCCTCC 450

5' ATCgTaGCTCCCTTCGCCCTTATtgTGGCCGCTCTGGTCTCTCACCtccTaCGctAAGaTTTAGTCTACCGCGCaTACCATCGGC 535

**B)** Contig01109

>MvContig01109 length=584 numreads=5

AGTATTtGAACATATtGCTGAGCGACGAAaTcAAGGCTCAAGACCGAGTGAACGGgTTCACTTTCCTGTTCATGGGCAACAGGGACAACGCTAAGGCAGCGCTGCCctCccTGAAGGCACGGCTCAaCGAGATCAGGACAGCTGtCGtgCTGCCTGCgaaCTTCAATtCtGTtCTGAGCAACACTGCTGcTtActtGGATGAAGAAGGTTtGGACGATATGGAGGAaTGGCTCCGAAGCAACGAAGCcACGATACCaGAAGCGGCTGCcGGtCTGAGCGCGATCGCgTCCGCtCGTTCCAGCCAGCAgTGGGgCACCACCAAagCCGATGAGAtCCTGAGTGCtGTCCGAGGATCCGCtGCcGTCATGGCTCCCACgCtCACCCtCcTCcTCATtGCAGCCATCGCCGCCATCTTGAAATaGAGCTGTCAtcGTCGATGACAGTCCAGCATTACAGATTCATaGatACAGCTATGGAATttGTCGGTGTACTGGTTACCATTGTTACAATGtaTTTTTtGTGCCTTTAAAAaTATATAAATATTAACATTTAgTATAAGCGAATCATGTATATTGTACGAGTAG

>MvContig01109 _AminoAcidTranslation

YLNILLSDEIKAQDRVNGFTFLFMGNRDNAKAALPSLKARLNEIRTAVVLPANFNSVLSNTAAYLDEEGLDDMEEWLRSNEATIPEAAAGLSAIASARSSQQWGTTKADEILSAVRGSAAVM

VIRTUAL RIBOSOME

>MvContig01109 Reading frame: 3 Translation table: Standard SGC0

Y L N I L L S D E I K A Q D R V N G F T F L F M G N R D N

5' AGTATTtGAACATATtGCTGAGCGACGAAaTcAAGGCTCAAGACCGAGTGAACGGgTTCACTTTCCTGTTCATGGGCAACAGGGACAACG 90

.....)))......)))))).............................................)))...>>>................

A K A A L P S L K A R L N E I R T A V V L P A N F N S V L S

5' CTAAGGCAGCGCTGCCctCccTGAAGGCACGGCTCAaCGAGATCAGGACAGCTGtCGtgCTGCCTGCgaaCTTCAATtCtGTtCTGAGCA 180

...........)))......)))....................................))).....................)))....

N T A A Y L D E E G L D D M E E W L R S N E A T I P E A A A

5' ACACTGCTGcTtActtGGATGAAGAAGGTTtGGACGATATGGAGGAaTGGCTCCGAAGCAACGAAGCcACGATACCaGAAGCGGCTGCcG 270

..............)))............)))......>>>.................................................

G L S A I A S A R S S Q Q W G T T K A D E I L S A V R G S A

5' GtCTGAGCGCGATCGCgTCCGCtCGTTCCAGCCAGCAgTGGGgCACCACCAAagCCGATGAGAtCCTGAGTGCtGTCCGAGGATCCGCtG 360

..)))............................................................)))......................

A V M

5' CcGTCATGGCTCCCACgCtCACCCtCcTCcTCATtGCAGCCATCGCCGCCATCTTGAAATaGAGCTGTCAtcGTCGATGACAGTCCAGCA 450

.....>>>

5' TTACAGATTCATaGatACAGCTATGGAATttGTCGGTGTACTGGTTACCATTGTTACAATGtaTTTTTtGTGCCTTTAAAAaTATATAAA 540

5' TATTAACATTTAgTATAAGCGAATCATGTATATTGTACGAGTAG 584

**C)** Contig01244

>Contig01244 length=253 numreads=2

GTTCCTtGCATCATAGTTTACTCTGTAGAAACCTGAGGCTTGTTTGTTGAAAACGaCCCATTcGTTtCCTTGTGTCCcACGCTCAATgACGGTCACTTGtGCAGAAAGGAActGGGAcGGTTTtAGATTTTGAAATCGGCGTCATTCTGTCGAGTCCACGTGATAGGAATGTCCcACAGACTAGGgAGGgTGGA

>Contig01244_AminoAcidTranslation

FQNLKPSQFLSAQVTVIERGTQGNEQVVFNKQASGFYRVNYD

VIRTUAL RIBOSOME

>MvContig01244 Reading frame: -3 Translation table: Standard SGC0

..........................................................................................

3' CAAGGAaCGTAGTATCAAATGAGACATCTTTGGACTCCGAACAAACAACTTTTGCtGGGTAAgCAAaGGAACACAGGgTGCGAGTTAcTG 90

D Y N V R Y F G S A Q K N F V V W E N G Q T G R E I V

.............................................

3' CCAGTGAACaCGTCTTTCCTTgaCCCTgCCAAAaTCTAAAACTTTAGCCGCAGTAAGACAGCTCAGGTGCACTATCCTTACAGGgTGTCT 180

T V Q A S L F Q S P K L N Q F

3' GATCCcTCCcACCT 194

**D)** Contig01676

>Contig01676 length=309 numreads=2

ACATGAACaCCGGAGCGATCACCTTCcAACAAGAACGATTCCTGCTCTCCGGAGCCACCCcACCcGACACCcTCTGGCACATCCCcATCACCTGGACCCACAGTGGgAACCCCAACTTCCAGAGCaCGaGGCCAaGCTTCATCCTCTCCACTCGGCAGCACACCATCAGCAACACCCcTGGTCACTTCTGGGTtATGCTCAACaTCGCTCAGTCAGGTTTGTACCGTGTCAACTACGATGACCACAACTGGGAAATGATTGCCGcTtaCCTTCGCAACGACAACACCAGgAACAATGTCcACAAGCTGA

>Contig01676_AminoAcidTranslation

MNTGAITFQQERFLLSGATPPDTLWHIPITWTHSGNPNFQSTRPSFILSTRQHTISNTPGHFWVMLNIAQSGLYRVNYDDHNWEMIAAYLRNDNTRNNVHKL

VIRTUAL RIBOSOME

>Contig01676 Reading frame: 3 Translation table: Standard SGC0

M N T G A I T F Q Q E R F L L S G A T P P D T L W H I P I

5' ACATGAACACCGGAGCGATCACCTTCCAACAAGAACGATTCCTGCTCTCCGGAGCCACCCCACCCGACACCCTCTGGCACATCCCCATCA 90

..>>>....................................)))..............................................

T W T H S G N P N F Q S T R P S F I L S T R Q H T I S N T P

5' CCTGGACCCACAGTGGGAACCCCAACTTCCAGAGCACGAGGCCAAGCTTCATCCTCTCCACTCGGCAGCACACCATCAGCAACACCCCTG 180

..........................................................................................

G H F W V M L N I A Q S G L Y R V N Y D D H N W E M I A A Y

5' GTCACTTCTGGGTTATGCTCAACATCGCTCAGTCAGGTTTGTACCGTGTCAACTACGATGACCACAACTGGGAAATGATTGCCGCTTACC 270

..............>>>.....................))).................................>>>.............

L R N D N T R N N V H K L

5' TTCGCAACGACAACACCAGGAACAATGTCCACAAGCTGA 309

...................................))).

**E)** Contig02305

>contig02305 length=413 numreads=3

TAGAAaGTACGCGAaaCatcGTTAGAAGTGCACCcGAGAGCATTGAGCAGGATGGACTGCTCACTGGAGTCAGAGgTGAGCAGGTACCGGCTCCACAAGAACTCGAAGTGGTCTGAGGAACCACcACGGAGACCGGAACAGAACACTGTCGTCTGGATGTCTGGGTGAGGGAGTTGACCAGTAGTCCTGTAGCTCTGCAGCAAATtcTCAGCGGTGTTGACGCAGTCCTCGTTGCCATAGCGACAGTTGAaGTTGAGGATGATGGTCCGGAAGTAaGCGTCGACGAACTCGTCTGTCGCCTGCTGGgCGAAGCCGAGCCTCTCGTACTGaGGAGcGgtCAGCTCTAGCACGTATCTCTGCCAAAGGTCATACACTTCAGTGCttgAAAGAACGATGTCAatGTAGTtGAAAGC

>contig02305_AminoAcidTranslation

AFNYIDIVLSSTEVYDLWQRYVLELTAPQYERLGFAQQATDEFVDAYFRTIILNFNCRYGNEDCVNTAENLLQSYRTTGQLPHPDIQTTVFCSGLRGGSSDHFEFLWSRYLLTSDSSEQSILLNALGCTSNDVSRTF

VIRTUAL RIBOSOME

>contig02305 Reading frame: -1 Translation table: Standard SGC0

...............................................(((..............................(((.......

3' ATCTTtCATGCGCTttGtagCAATCTTCACGTGGgCTCTCGTAACTCGTCCTACCTGACGAGTGACCTCAGTCTCcACTCGTCCATGGCC 90

F T R S V D N S T C G L A N L L I S Q E S S D S T L L Y R

.....(((..................................................................................

3' GAGGTGTTCTTGAGCTTCACCAGACTCCTTGGTGgTGCCTCTGGCCTTGTCTTGTGACAGCAGACCTACAGACCCACTCCCTCAACTGGT 180

S W L F E F H D S S G G R L G S C F V T T Q I D P H P L Q G

.................((((((...................................................................

3' CATCAGGACATCGAGACGTCGTTTAagAGTCGCCACAACTGCGTCAGGAGCAACGGTATCGCTGTCAACTtCAACTCCTACTACCAGGCC 270

T T R Y S Q L L N E A T N V C D E N G Y R C N F N L I I T R

....................................................................(((...................

3' TTCATtCGCAGCTGCTTGAGCAGACAGCGGACGACCcGCTTCGGCTCGGAGAGCATGACtCCTCgCcaGTCGAGATCGTGCATAGAGACG 360

F Y A D V F E D T A Q Q A F G L R E Y Q P A T L E L V Y R Q

.....................................................

3' GTTTCCAGTATGTGAAGTCACGaacTTTCTTGCTACAGTtaCATCAaCTTTCG 413

W L D Y V E T S S L V I D I Y N F A

**F)** Assembly of contig00522, contig00524, and contig 02783 using CAP3 (Huang and Madden 1999).

>MvContig00522 length=925 numreads=10

CTACGTAGAGCGCGATGGTGAATCCTTCATGGACGgCATtGGCCGGATGTAtGTCATGAACTtCCtGTGtGACATtGgAGACGAACAGTGCACcGTCACAGGCAAGACCTACTTCGATAACTGGAAGGATGGAGCATTtAtCCCaGCCAACaTGCGCCcTTGGGTGTACTGCgTtGGTCTCCGCGAGGGTAACGCTACGGATTTCGACTTCTTCTGGAACCAGTACTTGGCcGTAGACCTGGCCAGCGAGCAGGTGGTtATGCTGCAAGCCGCCGGGTGCACCAGTGACGTCGAAAGCCTGGAGAAGTTCTTGGATGCCATCGTTGCCGACGAAGACCTGGTCAGACCACAAGAcTtCACCACCGCtcTGGCGTCTGCCGTGAGAAGGAATGAATACAACACGCTTAGAGTATTTGATTGGTTGAAgaGGAGCTTGCCACAAGCTACTgCTACCCTTGGTGGCGTCGGTACTCTTCTGAGCTACATCGCTGCACGCCTTTTGAATGAGAACGATATTCaGGAGTTCGAAGCATGGCTGAACGAGAACCAAGCTGCTTtAGGCGCCGCTTACAACACCGGTATAAaCGGAGCTAACTCTGCAAGGAACAACCTGCAATGGTCTGAGCAGCGTctGCCGgAGTTTGTTAAGTACTTCGAGACGGGTTACCTTGAAGAtAATGTCGATGAACCGACCACTGCTGCCCCCGACCAAGAGACAGACGCGACTCTTGACCCCGAAGACAACTTCaCGACGGaGACACCCACAACTCTCCCTGATTCTGCCAACCTTGCCACCcTAaGCGTCTtAACGCTTGTGaTAACGTTGGCCATCAATCTtGTTAActAAATAGTATTTaTTGTTTTGTAAaTTAAATGATACAtAtCAtGtATTTTTtGtAaTTTCGTATTAAATGaTTGTTGAAAG

>MvContig00524 length=250 numreads=1

GAAGttcttggatgccatcGTCGCTGATGAAGACCTCGTCAGACCACAagactttacCACCGCATTGGCGTCTGCCGTGAGAAGGaatgaaTACAACAcccTCAGAGTAtttGAttggttgaagaggagcttgcCacaaGCTactgCTAcccTTGGTGGCGTCGGTACTCTTctgaGCTACATCGCTGCAcGCCttttgaatgagagcgatattcAggagtTCGAATCATGGCTGaacgagaaccaagca

>MvContig02783 length=645 numreads=11

TGCACcAGTGATGtCGACAGcctGGAGAAGTTCTTGGAtGCCATCGTCGCTGATGAAGACCTCGTCAGACCACAAGACTTCACCACCGCTTTGGCGTCTGCCGTGAGAAGGAAtGAATACAACACGCTCAGAGTGTTTGATtGGTTGAAGAGGAGCTTGCCACAagCTACTGCTACCCTTGGTGGCgTCGGTACTCTTCTGaGCTACATCGCTGCACGacTTTtGAATGAGAGCGATATTCAGGAGTTCGAATCATGGCTAAaCGAGAACCAAGCTGCtCTCGGCTCCGCTTACAaCACCGGTATAAaCGGAGCTAACTCTGCAAGGAACAACCTgCAATGGTCTGAGCTGCGTTtGCctGAGTTTGTTAAGTTCTTCGAGACGGGAtACGTtGACGACGATATCGATGAACCGGCCACTCCTGCCCCCATCGACCCTGAGACAGACGcGACTCTGGAACCCGAAGaTCCCTTcACAACGGAAACACcAACGACTCTACCtGaTTCTGCcAaCCTTGCGACCcTGAGCGTCCTAACGCTTATGGTAGCGTTAgCcATCAATCTTGTTAaCTAaagTATTTATTGTATtGTAAATTTAatGATGCATaTCATGtatATTTTtGTAaTTTCGtATTAAaTGATtGTT

Number of segment pairs = 56; number of pairwise comparisons = 3

'+' means given segment; '-' means reverse complement

Overlaps Containments No. of Constraints Supporting Overlap

******************* Contig 1 ********************

contig00522+

contig02783+ is in contig00522+

contig00524+ is in contig02783+

DETAILED DISPLAY OF CONTIGS

******************* Contig 1 ********************

. : . : . : . : . : . :

contig00522+ CTACGTAGAGCGCGATGGTGAATCCTTCATGGACGgCATtGGCCGGATGTAtGTCATGAA

____________________________________________________________

consensus CTACGTAGAGCGCGATGGTGAATCCTTCATGGACGGCATTGGCCGGATGTATGTCATGAA

. : . : . : . : . : . :

contig00522+ CTtCCtGTGtGACATtGgAGACGAACAGTGCACcGTCACAGGCAAGACCTACTTCGATAA

____________________________________________________________

consensus CTTCCTGTGTGACATTGGAGACGAACAGTGCACCGTCACAGGCAAGACCTACTTCGATAA

. : . : . : . : . : . :

contig00522+ CTGGAAGGATGGAGCATTtAtCCCaGCCAACaTGCGCCcTTGGGTGTACTGCgTtGGTCT

____________________________________________________________

consensus CTGGAAGGATGGAGCATTTATCCCAGCCAACATGCGCCCTTGGGTGTACTGCGTTGGTCT

. : . : . : . : . : . :

contig00522+ CCGCGAGGGTAACGCTACGGATTTCGACTTCTTCTGGAACCAGTACTTGGCcGTAGACCT

____________________________________________________________

consensus CCGCGAGGGTAACGCTACGGATTTCGACTTCTTCTGGAACCAGTACTTGGCCGTAGACCT

. : . : . : . : . : . :

contig00522+ GGCCAGCGAGCAGGTGGTtATGCTGCAAGCCGCCGGGTGCACCAGTGACGTCGAAAGCCT

contig02783+ TGCACcAGTGATGtCGACAGcct

____________________________________________________________

consensus GGCCAGCGAGCAGGTGGTTATGCTGCAAGCCGCCGGGTGCACCAGTGACGTCGAAAGCCT

. : . : . : . : . : . :

contig00522+ GGAGAAGTTCTTGGATGCCATCGTTGCCGACGAAGACCTGGTCAGACCACAAGAcTtCAC

contig02783+ GGAGAAGTTCTTGGAtGCCATCGTCGCTGATGAAGACCTCGTCAGACCACAAGACTTCAC

contig00524+ GAAGttcttggatgccatcGTCGCTGATGAAGACCTCGTCAGACCACAagactttac

____________________________________________________________

consensus GGAGAAGTTCTTGGATGCCATCGTCGCTGATGAAGACCTCGTCAGACCACAAGACTTCAC

. : . : . : . : . : . :

contig00522+ CACCGCtcTGGCGTCTGCCGTGAGAAGGAATGAATACAACACGCTTAGAGTATTTGATTG

contig02783+ CACCGCTTTGGCGTCTGCCGTGAGAAGGAAtGAATACAACACGCTCAGAGTGTTTGATtG

contig00524+ CACCGCATTGGCGTCTGCCGTGAGAAGGaatgaaTACAACAcccTCAGAGTAtttGAttg

____________________________________________________________

consensus CACCGCTTTGGCGTCTGCCGTGAGAAGGAATGAATACAACACGCTCAGAGTATTTGATTG

. : . : . : . : . : . :

contig00522+ GTTGAAgaGGAGCTTGCCACAAGCTACTgCTACCCTTGGTGGCGTCGGTACTCTTCTGAG

contig02783+ GTTGAAGAGGAGCTTGCCACAagCTACTGCTACCCTTGGTGGCgTCGGTACTCTTCTGaG

contig00524+ gttgaagaggagcttgcCacaaGCTactgCTAcccTTGGTGGCGTCGGTACTCTTctgaG

____________________________________________________________

consensus GTTGAAGAGGAGCTTGCCACAAGCTACTGCTACCCTTGGTGGCGTCGGTACTCTTCTGAG

. : . : . : . : . : . :

contig00522+ CTACATCGCTGCACGCCTTTTGAATGAGAACGATATTCaGGAGTTCGAAGCATGGCTGAA

contig02783+ CTACATCGCTGCACGacTTTtGAATGAGAGCGATATTCAGGAGTTCGAATCATGGCTAAa

contig00524+ CTACATCGCTGCAcGCCttttgaatgagagcgatattcAggagtTCGAATCATGGCTGaa

____________________________________________________________

consensus CTACATCGCTGCACGCCTTTTGAATGAGAGCGATATTCAGGAGTTCGAATCATGGCTGAA

. : . : . : . : . : . :

contig00522+ CGAGAACCAAGCTGCTTtAGGCGCCGCTTACAACACCGGTATAAaCGGAGCTAACTCTGC

contig02783+ CGAGAACCAAGCTGCtCTCGGCTCCGCTTACAaCACCGGTATAAaCGGAGCTAACTCTGC

contig00524+ cgagaaccaagc

____________________________________________________________

consensus CGAGAACCAAGCTGCTCTAGGCGCCGCTTACAACACCGGTATAAACGGAGCTAACTCTGC

. : . : . : . : . : . :

contig00522+ AAGGAACAACCTGCAATGGTCTGAGCAGCGTctGCCGgAGTTTGTTAAGTACTTCGAGAC

contig02783+ AAGGAACAACCTgCAATGGTCTGAGCTGCGTTtGCctGAGTTTGTTAAGTTCTTCGAGAC

____________________________________________________________

consensus AAGGAACAACCTGCAATGGTCTGAGCAGCGTCTGCCGGAGTTTGTTAAGTACTTCGAGAC

. : . : . : . : . : . :

contig00522+ GGGTTACCTTGAAGAtAATGTCGATGAACCGACCACTGCTGCCCCC---GACCAAGAGAC

contig02783+ GGGAtACGTtGACGACGATATCGATGAACCGGCCACTCCTGCCCCCATCGACCCTGAGAC

____________________________________________________________

consensus GGGATACCTTGAAGACAATATCGATGAACCGACCACTCCTGCCCCCATCGACCAAGAGAC

. : . : . : . : . : . :

contig00522+ AGACGCGACTCTTGACCCCGAAGACAACTTCaCGACGGaGACACCCACAACTCTCCCTGA

contig02783+ AGACGcGACTCTGGAACCCGAAGaTCCCTTcACAACGGAAACACcAACGACTCTACCtGa

____________________________________________________________

consensus AGACGCGACTCTGGAACCCGAAGACAACTTCACAACGGAAACACCAACAACTCTACCTGA

. : . : . : . : . : . :

contig00522+ TTCTGCCAACCTTGCCACCcTAaGCGTCTtAACGCTTGTGaTAACGTTGGCCATCAATCT

contig02783+ TTCTGCcAaCCTTGCGACCcTGAGCGTCCTAACGCTTATGGTAGCGTTAgCcATCAATCT

____________________________________________________________

consensus TTCTGCCAACCTTGCCACCCTAAGCGTCCTAACGCTTATGATAACGTTAGCCATCAATCT

. : . : . : . : . : . :

contig00522+ tGTTAActAAATAGTATTTaTTGTTTTGTAAaTTAAATGATACAtAtCAtGtAT-TTTTt

contig02783+ TGTTAaCTAaa--gTATTTATTGTATtGTAAATTTAatGATGCATaTCATGtatATTTTt

____________________________________________________________

consensus TGTTAACTAAATAGTATTTATTGTATTGTAAATTAAATGATACATATCATGTATATTTTT

. : . : . : . : . : . :

contig00522+ GtAaTTTCGTATTAAATGaTTGTTGAAAG

contig02783+ GTAaTTTCGtATTAAaTGATtGTT

____________________________________________________________

consensus GTAATTTCGTATTAAATGATTGTTGAAAG

>Contig00522_00524_027831_consensus

CTACGTAGAGCGCGATGGTGAATCCTTCATGGACGGCATTGGCCGGATGTATGTCATGAACTTCCTGTGTGACATTGGAGACGAACAGTGCACCGTCACAGGCAAGACCTACTTCGATAACTGGAAGGATGGAGCATTTATCCCAGCCAACATGCGCCCTTGGGTGTACTGCGTTGGTCTCCGCGAGGGTAACGCTACGGATTTCGACTTCTTCTGGAACCAGTACTTGGCCGTAGACCTGGCCAGCGAGCAGGTGGTTATGCTGCAAGCCGCCGGGTGCACCAGTGACGTCGAAAGCCTGGAGAAGTTCTTGGATGCCATCGTCGCTGATGAAGACCTCGTCAGACCACAAGACTTCACCACCGCTTTGGCGTCTGCCGTGAGAAGGAATGAATACAACACGCTCAGAGTATTTGATTGGTTGAAGAGGAGCTTGCCACAAGCTACTGCTACCCTTGGTGGCGTCGGTACTCTTCTGAGCTACATCGCTGCACGCCTTTTGAATGAGAGCGATATTCAGGAGTTCGAATCATGGCTGAACGAGAACCAAGCTGCTCTAGGCGCCGCTTACAACACCGGTATAAACGGAGCTAACTCTGCAAGGAACAACCTGCAATGGTCTGAGCAGCGTCTGCCGGAGTTTGTTAAGTACTTCGAGACGGGATACCTTGAAGACAATATCGATGAACCGACCACTCCTGCCCCCATCGACCAAGAGACAGACGCGACTCTGGAACCCGAAGACAACTTCACAACGGAAACACCAACAACTCTACCTGATTCTGCCAACCTTGCCACCCTAAGCGTCCTAACGCTTATGATAACGTTAGCCATCAATCTTGTTAACTAAATAGTATTTATTGTATTGTAAATTAAATGATACATATCATGTATATTTTTGTAATTTCGTATTAAATGATTGTTGAAAG

>Contig_00522,00524,027831_consensus_AminoAcidTranslation

YVERDGESFMDGIGRMYVMNFLCDIGDEQCTVTGKTYFDNWKDGAFIPANMRPWVYCVGLREGNATDFDFFWNQYLAVDLASEQVVMLQAAGCTSDVESLEKFLDAIVADEDLVRPQDFTTALASAVRRNEYNTLRVFDWLKRSLPQATATLGGVGTLLSYIAARLLNESDIQEFESWLNENQAALGAAYNTGINGANSARNNLQWSEQRLPEFVKYFETGYLEDNIDEPTTPAPIDQETDATLEPEDNFTTETPTTLPDSANLATLSVLTLMITLAINLVN

VIRTUAL RIBOSOME

>Contig_00522,00524,027831_consensus Reading frame: 2

Translation table: Standard SGC0

Y V E R D G E S F M D G I G R M Y V M N F L C D I G D E Q C

5' CTACGTAGAGCGCGATGGTGAATCCTTCATGGACGGCATTGGCCGGATGTATGTCATGAACTTCCTGTGTGACATTGGAGACGAACAGTG 90

............................>>>...............>>>......>>>......))).......................

T V T G K T Y F D N W K D G A F I P A N M R P W V Y C V G L

5' CACCGTCACAGGCAAGACCTACTTCGATAACTGGAAGGATGGAGCATTTATCCCAGCCAACATGCGCCCTTGGGTGTACTGCGTTGGTCT 180

.............................................................>>>..........................

R E G N A T D F D F F W N Q Y L A V D L A S E Q V V M L Q A

5' CCGCGAGGGTAACGCTACGGATTTCGACTTCTTCTGGAACCAGTACTTGGCCGTAGACCTGGCCAGCGAGCAGGTGGTTATGCTGCAAGC 270

..............................................))).........)))..................>>>))).....

A G C T S D V E S L E K F L D A I V A D E D L V R P Q D F T

5' CGCCGGGTGCACCAGTGACGTCGAAAGCCTGGAGAAGTTCTTGGATGCCATCGTCGCTGATGAAGACCTCGTCAGACCACAAGACTTCAC 360

............................))).........)))...............................................

T A L A S A V R R N E Y N T L R V F D W L K R S L P Q A T A

5' CACCGCTTTGGCGTCTGCCGTGAGAAGGAATGAATACAACACGCTCAGAGTATTTGATTGGTTGAAGAGGAGCTTGCCACAAGCTACTGC 450

.......)))...................................................))).........)))..............

T L G G V G T L L S Y I A A R L L N E S D I Q E F E S W L N

5' TACCCTTGGTGGCGTCGGTACTCTTCTGAGCTACATCGCTGCACGCCTTTTGAATGAGAGCGATATTCAGGAGTTCGAATCATGGCTGAA 540

.........................))).....................))).................................)))..

E N Q A A L G A A Y N T G I N G A N S A R N N L Q W S E Q R

5' CGAGAACCAAGCTGCTCTAGGCGCCGCTTACAACACCGGTATAAACGGAGCTAACTCTGCAAGGAACAACCTGCAATGGTCTGAGCAGCG 630

......................................................................))).................

L P E F V K Y F E T G Y L E D N I D E P T T P A P I D Q E T

5' TCTGCCGGAGTTTGTTAAGTACTTCGAGACGGGATACCTTGAAGACAATATCGATGAACCGACCACTCCTGCCCCCATCGACCAAGAGAC 720

.)))......................................................................................

D A T L E P E D N F T T E T P T T L P D S A N L A T L S V L

5' AGACGCGACTCTGGAACCCGAAGACAACTTCACAACGGAAACACCAACAACTCTACCTGATTCTGCCAACCTTGCCACCCTAAGCGTCCT 810

..........))).............................................................................

T L M I T L A I N L V N *

5' AACGCTTATGATAACGTTAGCCATCAATCTTGTTAACTAAATAGTATTTATTGTATTGTAAATTAAATGATACATATCATGTATATTTTT 900

.......>>>...........................***

5' GTAATTTCGTATTAAATGATTGTTGAAAG
