# Supplementary material for: Transcriptome Sequencing, and Rapid Development and Application of SNP Markers for the Legume Pod Borer Maruca vitrata (Lepidoptera: Crambidae)
Source: PLoS One. 2011 Jul 6;6(7):e21388. doi: 10.1371/journal.pone.0021388 (PMC3130784; doi:10.1371/journal.pone.0021388)
Supplement: Data S3 — CLUSTAL 2.0.12 multiple sequence alignments of derived Maruca vitrata alanine aminopeptidase (APN) amino acid sequences with nine Bombyx mori APNs from the GLEAN-predicted gene model v. 2.3. (DOC) [file pone.0021388.s003.doc]

**Supplemental Data S3:** CLUSTAL 2.0.12 multiple sequence alignments of derived *Maruca vitrata* alanine aminopeptidase (APN) amino acid sequences with nine *Bombyx mori* APNs from the GLEAN-predicted gene model v. 2.3.

**A)** Contig00544_AminoAcidTranslation

contig00544 -------------------------------SENTADMVVILRALACTRD 19

BGIBMGA008060 VPVNSRRYVYCVGVRQGNSSDYNFLFERYNASQNTADMVVMLRALACTRD 744

BGIBMGA008061 S-PQIRRNVYMTGMREGDSSDFEYLLNRFRQSNFANDQLEMLRGLGASKD 743

BGIBMGA008062 N-PNLRRHVFCSGLLEGGYNEWRFLYERRKNSNNQGDEVAMLRSLGCTTN 808

BGIBMGA008059 IPANMRPWVYCAGLRHGTAEDFNFFWNRYLQEDLSSEKVVMLNVAGCTTD 1649

BGIBMGA008017 LNPDLQNTVYCSGLRGGDRDNFNFLWEQYLASSDSSEQNILLNALGC--- 783

BGIBMGA008063 S-PSLRPVTYCSGLRFGDASDYEFLWERMSTTNVANEARVISGVLGCSTD 784

BGIBMGA008018 -NNEEVPAVYCTMVREGDEDVIEALKARLEVEANHYERVVILESLACSDD 827

BGIBMGA001642 P-EIYQEAAFTAGVRTHGRVAWRACWRALVDSYSAP-------------- 491

BGIBMGA001641 ----LLAQQWFGGLVSPRWWASQWLMEALTS------------------- 511

contig00544 A-LLQTYLGQTLSN-DRIRAHDRTNAFLYALQ------------------ 49

BGIBMGA008060 TNSLQHYMFQSMHN-DRIRIHDRTNAFSYALQ------------------ 775

BGIBMGA008061 SQLLTRYLQLTLT--REVRSHDKATSFNYALL------------------ 773

BGIBMGA008062 PQARQEYLSMILS--DDVKAQDRVNALTFFYM------------------ 838

BGIBMGA008059 QASLNRFLDAIVSGNDDIRPQDYNAALTSAIT------------------ 1681

BGIBMGA008017 ------YMNQVIDANSPVREQDRHTILVSVIN------------------ 809

BGIBMGA008063 EGSLEKFLFSVKEENSPIRTQDLTVPLTGVLSNHSHIYIVMDSLKQNYSA 834

BGIBMGA008018 QNFIRNYLEETIAAGNEYGVEERVRIFRAVAES----------------- 860

BGIBMGA001642 ------------------RPTYSHRALLAALAS----------------- 506

BGIBMGA001641 -----------------LIAEKAPPFKNSALK------------------ 526

contig00544 -------------------GNRESRDIVLDFLFDNFEAIREEHGGQARLN 80

BGIBMGA008060 -------------------GNRENLPIVLNFLYQNFAAIRTSYGGEARLV 806

BGIBMGA008061 -------------------GNQENANTVLQFVKNNIAAIRTAYIEDAPPT 804

BGIBMGA008062 -------------------GDRSNANVALQYLKENFEEIRQG---VVLPA 866

BGIBMGA008059 -------------------SNEINTLRAFQWLRNNVDQATRTLG---SVS 1709

BGIBMGA008017 -------------------SSPENMDAALEFVIENFHRIQPRVQGLTGTT 840

BGIBMGA008063 WSSIKCSNYHICMVSKLNILNGVNQFIVFAFLKRGRGVVQDDLVGFAGAN 884

BGIBMGA008018 --------------------SYENARVALSFISMRTNEIRDNYGGPKKLE 890

BGIBMGA001642 ---------------------PEDDWLFYRFAFTVLSTEAQRGRDWTEWI 535

BGIBMGA001641 ----------------------QEEALLLDHVLPALR------------- 541

.

contig00544 VNINNCAAFLTQFTQIQR-------------------------------- 98

BGIBMGA008060 TAVNAISGFLTDFAIIRENTWGYQIYNGQNVQVFGLDTSGSLVVTLAEGL 856

BGIBMGA008061 PVHTALSNLAAYLDESGLD------------------------------- 823

BGIBMGA008062 WFDNVISNLASYLNEEGLE------------------------------- 885

BGIBMGA008059 TILNTIIGRLLNEEQIN--------------------------------- 1726

BGIBMGA008017 NILNAFARRLTTETHAER-------------------------------- 858

BGIBMGA008063 FDRVGCKHQCFRARQERTTPKRFHRENRTDKIHDLQETLRRRTIPKFKFK 934

BGIBMGA008018 EILFVLGENMANDILSED-------------------------------- 908

BGIBMGA001642 TALYTSTCR----------------------------------------- 544

BGIBMGA001641 --------------------------------------------------

contig00544 --------------------------------------FQTWAYANQIAL 110

BGIBMGA008060 LDEGGCMITDNYYTSDPLT--EFMLSRNTDLCGTIREKFQSWVYANQLAL 904

BGIBMGA008061 -------------------------------------EYETWLRSTQTNI 836

BGIBMGA008062 -------------------------------------DMESWLRANQNMI 898

BGIBMGA008059 -------------------------------------EVSNWLTANQNTL 1739

BGIBMGA008017 --------------------------------------INQLISRHQAIL 870

BGIBMGA008063 FKYFRMFFALLLFVLFSYSNANFIVEEECLNYTVYPIQYELTIIPYIYKD 984

BGIBMGA008018 --------------------------------------FRIWVRSQSNDL 920

BGIBMGA001642 --------------------------------------------------

BGIBMGA001641 --------------------------------------------------

contig00544 GS------------------------------------------------ 112

BGIBMGA008060 GTAFSTGVSVINSAISN-LEWG---------------------------- 925

BGIBMGA008061 PQYN-SALSAINSARSN-IAWG---------------------------- 856

BGIBMGA008062 PNFN-VGLNAINSARTS-MQWG---------------------------- 918

BGIBMGA008059 GATYSTALRAIETTRSN-LVWSQQRISEFTNYFESGYVEDVIEEITEAPP 1788

BGIBMGA008017 TAGEQASISAIREHIAASIAWGK-----------------------DNAA 897

BGIBMGA008063 NSYYHGDITITVIANANVREIELDAKDLDIQSGSIKVLDGSTDLVNGARP 1034

BGIBMGA008018 DDSQGAANRALAIVLENVNWIER--------------------------- 943

BGIBMGA001642 --------------------------------------------------

BGIBMGA001641 --------------------------------------------------

**B)** Contig01109_AminoAcidTranslation

MvContig01109 ---------YLNILLS--DEIKAQDRVNGFTFLFM--------------- 24

BGIBMGA008062 TTNPQARQEYLSMILS--DDVKAQDRVNALTFFYM--------------- 838

BGIBMGA008061 SKDSQLLTRYLQLTLT--REVRSHDKATSFNYALL--------------- 773

BGIBMGA008060 TRDTNSLQHYMFQSMHN-DRIRIHDRTNAFSYALQ--------------- 775

BGIBMGA008059 TTDQASLNRFLDAIVSGNDDIRPQDYNAALTSAIT--------------- 1681

BGIBMGA008017 ---------YMNQVIDANSPVREQDRHTILVSVIN--------------- 809

BGIBMGA008063 STDEGSLEKFLFSVKEENSPIRTQDLTVPLTGVLSNHSHIYIVMDSLKQN 831

BGIBMGA008018 SDDQNFIRNYLEETIAAGNEYGVEERVRIFRAVAES-------------- 860

BGIBMGA001642 PEDDWLFYRFAFTVLST-EAQRGRDWTEWITALYT--------------- 540

BGIBMGA001641 KQEEALLLDHVLPALR---------------------------------- 541

.

MvContig01109 ----------------------GNRDNAKAALPSLKARLNEIRTAVVLPA 52

BGIBMGA008062 ----------------------GDRSNANVALQYLKENFEEIRQGVVLPA 866

BGIBMGA008061 ----------------------GNQENANTVLQFVKNNIAAIRTAYIEDA 801

BGIBMGA008060 ----------------------GNRENLPIVLNFLYQNFAAIRTSYGGEA 803

BGIBMGA008059 ----------------------SNEINTLRAFQWLRNNVDQATRTLGSVS 1709

BGIBMGA008017 ----------------------SSPENMDAALEFVIENFHRIQPRVQGLT 837

BGIBMGA008063 YSAWSSIKCSNYHICMVSKLNILNGVNQFIVFAFLKRGRGVVQDDLVGFA 881

BGIBMGA008018 -----------------------SYENARVALSFISMRTNEIRDNYGGPK 887

BGIBMGA001642 ---------------------------------------STCR------- 544

BGIBMGA001641 --------------------------------------------------

MvContig01109 N------------------------------------------------- 53

BGIBMGA008062 W------------------------------------------------- 867

BGIBMGA008061 PP------------------------------------------------ 803

BGIBMGA008060 RL------------------------------------------------ 805

BGIBMGA008059 T------------------------------------------------- 1710

BGIBMGA008017 G------------------------------------------------- 838

BGIBMGA008063 GANFDRVGCKHQCFRARQERTTPKRFHRENRTDKIHDLQETLRRRTIPKF 931

BGIBMGA008018 K------------------------------------------------- 888

BGIBMGA001642 --------------------------------------------------

BGIBMGA001641 --------------------------------------------------

MvContig01109 --------------------------------------------------

BGIBMGA008062 --------------------------------------------------

BGIBMGA008061 --------------------------------------------------

BGIBMGA008060 --------------------------------------------------

BGIBMGA008059 --------------------------------------------------

BGIBMGA008017 --------------------------------------------------

BGIBMGA008063 KFKFKYFRMFFALLLFVLFSYSNANFIVEEECLNYTVYPIQYELTIIPYI 981

BGIBMGA008018 --------------------------------------------------

BGIBMGA001642 --------------------------------------------------

BGIBMGA001641 --------------------------------------------------

MvContig01109 -------------------------------------------FNSVLS- 59

BGIBMGA008062 -------------------------------------------FDNVIS- 873

BGIBMGA008061 -----------------------------------------TPVHTALS- 811

BGIBMGA008060 -----------------------------------------VTAVNAISG 814

BGIBMGA008059 ------------------------------------------ILNTIIG- 1717

BGIBMGA008017 -------------------------------------------TTNILN- 844

BGIBMGA008063 YKDNSYYHGDITITVIANANVREIELDAKDLDIQSGSIKVLDGSTDLVNG 1031

BGIBMGA008018 -------------------------------------------LEEILF- 894

BGIBMGA001642 --------------------------------------------------

BGIBMGA001641 --------------------------------------------------

MvContig01109 ------------------------------------NTAAYLDEEG---- 69

BGIBMGA008062 ------------------------------------NLASYLNEEG---- 883

BGIBMGA008061 ------------------------------------NLAAYLDESG---- 821

BGIBMGA008060 FLTDFAIIRENTWGYQIYNGQNVQVFGLDTSGSLVVTLAEGLLDEGGCMI 864

BGIBMGA008059 ---------------------------------------RLLNEEQ---- 1724

BGIBMGA008017 ------------------------------------AFARRLTTET---- 854

BGIBMGA008063 ARPYEYDKTNGKLFIHLREPLKVYSQNNRQFYYIKMSFNKYIKEDSAGLF 1081

BGIBMGA008018 ------------------------------------VLGENMANDILS-- 906

BGIBMGA001642 --------------------------------------------------

BGIBMGA001641 --------------------------------------------------

MvContig01109 ---------------------------------------LDDMEEWLRSN 80

BGIBMGA008062 ---------------------------------------LEDMESWLRAN 894

BGIBMGA008061 ---------------------------------------LDEYETWLRST 832

BGIBMGA008060 TDNYYTSDPL--------------TEFMLSRNTDLCGTIREKFQSWVYAN 900

BGIBMGA008059 ---------------------------------------INEVSNWLTAN 1735

BGIBMGA008017 --------------------------------------HAERINQLISRH 866

BGIBMGA008063 LVNYYEDDVKNAKSLYATRLSPNKAKFMFPCFDNPRFEAVFKFKVYILSD 1131

BGIBMGA008018 ----------------------------------------EDFRIWVRSQ 916

BGIBMGA001642 --------------------------------------------------

BGIBMGA001641 --------------------------------------------------

MvContig01109 EATIP-EAAAGLSAIASARSS----------------------------- 100

BGIBMGA008062 QNMIP-NFNVGLNAINSARTS----------------------------- 914

BGIBMGA008061 QTNIP-QYNSALSAINSARSN----------------------------- 852

BGIBMGA008060 QLALGTAFSTGVSVINSAISN----------------------------- 921

BGIBMGA008059 QNTLGATYSTALRAIETTRSN----------------------------- 1756

BGIBMGA008017 QAILTAGEQASISAIREHIAAS---------------------------- 888

BGIBMGA008063 HPGMQYTNSSLVIAEEMKRVSSKDYTIIEYIPSPQVALHQVGFHYSQFTN 1181

BGIBMGA008018 SNDLDDSQGAANRALAIVLEN----------------------------- 937

BGIBMGA001642 --------------------------------------------------

BGIBMGA001641 --------------------------------------------------

MvContig01109 -----------QQWG--------------------------------TTK 107

BGIBMGA008062 -----------MQWG--------------------------------TDR 921

BGIBMGA008061 -----------IAWG--------------------------------TAN 859

BGIBMGA008060 -----------LEWG--------------------------------NAE 928

BGIBMGA008059 -----------LVWSQQRISEFTNYFESGYVEDVIEEITEAPPTAPPTAP 1795

BGIBMGA008017 -----------IAWGK-----------------------DNAAVVEDWLE 904

BGIBMGA008063 KSVKGKNNDTLVIWAPGDKLYCYDYIQRFGISMIDLMHQYEGTKRPITAG 1231

BGIBMGA008018 -----------VNWIER--------------------------------H 944

BGIBMGA001642 --------------------------------------------------

BGIBMGA001641 --------------------------------------------------

MvContig01109 ADEILSAVRGSAAVM----------------------------------- 122

BGIBMGA008062 AQEILKAARGSAVTVLPTFMLLVPTLAMLVLK------------------ 953

BGIBMGA008061 AEMLLAAARDSATAVVTSAALLAITTLFALIM------------------ 891

BGIBMGA008060 ATDIYNFLLARSSSTTVTSSFILMITALVVKMFH---------------- 962

BGIBMGA008059 PTEAPAVTPAPDSANVAALSFITLIITLAVNLA----------------- 1828

BGIBMGA008017 DNYGEPKPEEPSAAHSTTAGFIVLLSAFVAFFNIH--------------- 939

BGIBMGA008063 SINLVAVPTNLQGYEIGSWNLLTNGANRIANLVYITSIQQIEVMRFELAQ 1281

BGIBMGA008018 QDYVYDWIDENDAPTLAASLILICITIFVTLFNN---------------- 978

BGIBMGA001642 --------------------------------------------------

BGIBMGA001641 --------------------------------------------------

**C)** Contig01244

Contig01244 ----------FQNLKPSQFLSAQVTVIERGTQGNEQVVFNKQASGFYRVN 40

BGIBMGA008059 ITWTRAGAPEFEDLKPSQFISQQVTSINRGTTGLEWVIFNKQEAGFYRVN 1450

BGIBMGA008061 --------------------------------------------------

BGIBMGA00806 --------------------------------------------------

BGIBMGA008060 --------------------------------------------------

BGIBMGA008063 ISYTIQNSKNCFNCYRPRFVIGSQPYTFIENLDGGWIILNSNGSGYYRVN 1537

BGIBMGA008017 --------------------------------------------------

BGIBMGA008018 --------------------------------------------------

**D)** Contig01676_AminoAcidTranslation

MvContig01676 --------------------------MNTGAITFQQERFLLS-GATPPDT 23

BGIBMGA008060 PNVDVGEILDSWVQNPGSPVINVEFNTNNGVITLTQERFLLT-GSR--DQ 490

BGIBMGA008061 SNFNFEEYYRIWVNEPGYPLLTVTVDHGDGTITLSQERFFLSASATPTDH 492

BGIBMGA008062 TTFDVEEFMKYWVDEPGFPLLDVAVNTETGVISLKQERFFISTSATQTNQ 555

BGIBMGA008063 G-ISITDVMKTWITQAGHPLLHVHVNYTDDTVTLTQKRFYIN-SSHSSNE 528

BGIBMGA008059 PDFSFVDYYKSWTEQSGHPVLNVQVNHQTGDMTIYQRRFNINTGYSNVNT 511

BGIBMGA008017 IPYSISNVMNRWVNQGGFPVLNVRKSAPNANSVFISQERYLTDRSLTSTD 529

BGIBMGA008018 MIDDIESFLEPWIENNGYPMIHVGLRQ-DGSVFIMQERFSFT---PQAHV 571

BGIBMGA001642 ---TMHNGTWIAVIVGNWPWPSAELSPRLGDTGMLQIVLHPN----AMIR 256

BGIBMGA001641 ADQTYIEFKEKIRRKYNYTLSLRFITRLERSDKQRGFFLTGNQRHRCAVS 303

MvContig01676 LWHIPITWTHSGNPNFQSTRPSFILSTRQHTISNT----PGHFWVMLNIA 69

BGIBMGA008060 LWRIPITWTDATTRNFSNTRPSLIMNTRTVNIQGN----AGQHWVMLNIA 536

BGIBMGA008061 IYPIPITYSSKSNNNFDNVRPVHIMTTKTDAFNVG----VKGEWVIFNNL 538

BGIBMGA008062 IWPIPLTYTTGSNPNWNSLRPLHIMTAQNDEIRIT----PGNQWVIFNVQ 601

BGIBMGA008063 TYSIPITYTTGQSPDFENTKPVFVMTGKTHEFQITNI-SKTHTWVIFNIQ 577

BGIBMGA008059 NYIVPITFATARNPNFANTKPTHVLTKAVTVINRG---SVGDEWVIFNKQ 558

BGIBMGA008017 RWHVPVNWVLSSNIDFSDTKPQGWIPPSFPATSIDIPGLANAEWFIFNKQ 579

BGIBMGA008018 NYEIPITYTTKASPNFNNIRPILMMDATTN-LNVR---LTGDEWVLFNIQ 617

BGIBMGA001642 RYDIGNETKPLQTSRWTENTTHLVWMNDTEMVIPDLG---KHKWIRYNVG 303

BGIBMGA001641 RFWLTHARSTFPCFDEPNLRASFKLTIVRDRFHVS-----LTNMPIVATE 348

MvContig01676 QSGLYRVNYDDHNWEMIAAYLRNDNTRNNVHKL----------------- 102

BGIBMGA008060 QSGLYRVNYDDSTWQRIAAFLRTN--REAVHKLNRAQIVNDVLFFIRAGK 584

BGIBMGA008061 QHGHYRVTYDDTTWNLIADALLNE--RESIHHLNRAEIVDDIFALMRSGR 586

BGIBMGA008062 QKGIYRVNYNQENWERLANALSED--HTNIHHLNRAQIVDDVFALMRSEK 649

BGIBMGA008063 ETGFYRVTYDEHTWEHIGGALKGS-SREKIHHLNRAKIVNDLFDLYYADE 626

BGIBMGA008059 QTGFYRVNYDDYTWNLIVIALRGP-QRTQIHEYNRAQIVNDVFQFARSGL 607

BGIBMGA008017 QTGYYRVNYDPENWAALARVLQTN--HAVIHLLNRAQILDDSFNMARNGR 627

BGIBMGA008018 GQSYYRVNYDDDIWDRILEALEDPEDRKVIHPLNRAKLVDDALNLARSGK 667

BGIBMGA001642 ARGLYRVAPQDRAGEEASDAARVY---DGASAAERALILDDAFVLSRAGR 350

BGIBMGA001641 EAGFYLGHRLLQDEFATSPPMPPHMVAVAVCRLQRASAPTPEANTTDATE 398

**E)** Contig02305_AminoAcidTranslation

MvContig02305 ------------------------------------------------AF 2

BGIBMGA008017 IHLLNRAQILDDSFNMARNGRLNYNLPFEISRYLINEKDYIPWAAINPAF 656

BGIBMGA008061 IHHLNRAEIVDDIFALMRSGRMTYSFGFKILRFLRSESNYHVWDAAITGY 615

BGIBMGA008062 IHHLNRAQIVDDVFALMRSEKLSFDLGFRVLDFLKKDTSYYVWYPAVTGF 678

BGIBMGA008060 VHKLNRAQIVNDVLFFIRAGKITTSRAFDVLSFLENERDYYVWAGAITQL 613

BGIBMGA008059 IHQLNRAQIVDDIFQLARANVMKYNRAFNILSFLQFEDEYAPWLAAISGF 1520

BGIBMGA008063 IHHLNRAKIVNDLFDLYYADEVSFSLLIETLEFLKEETENAVWFAAIKGL 655

BGIBMGA008018 IHPLNRAKLVDDALNLARSGKLDYEIAFKVVLSMEHETEYAVWKAFVRNM 696

BGIBMGA001642 ASAAERALILDDAFVLSRAGRLPASRAIVVAARIRGEQHWAPWRVVLSHM 379

BGIBMGA001641 SPPMPPHMVAVAVCRLQRAS--APTPEANTTDATEAETDGDTSTAPEISL 413

MvContig02305 NYID-IVLSSTEV-----YDLWQRYVLELTAPQYERLGFAQQA--TDEFV 44

BGIBMGA008017 NYLD-IVLTGSSV-----YNLFREYLLTLTAPLYDEIGWEATA--NEEHV 698

BGIBMGA008061 TWLRNRLRHLPD------QATFDAYILELMETVINTYGFDAAA--NEPPT 657

BGIBMGA008062 GWLRNRFLHMPDV-----LAEFNTILYTFLEAVIADLGYDVVD--GEPLT 721

BGIBMGA008060 EWIRRRLEHLPQA-----HEAFTAYTLDLLRNVINHLGYNERA--TDSTS 656

BGIBMGA008059 NFLIRRLAHDSTN-----AALLQKLILELSPAVVAKLGYLEPE--NGSYM 1563

BGIBMGA008063 NKLWNSYLGDSAL-----EDIER-LALKFIDNAITTIGYEVRA--TDDFS 697

BGIBMGA008018 DFLRKRLIAHVTEDDDLDPDIYMRMVRRTVGALEDEIGFNPDTSLTEPAM 746

BGIBMGA001642 SWWRELLREGAAAP----------ALARLLATLHPPIALRHQR-DADSDD 418

BGIBMGA001641 YTDHPSILQESGP------------LLEWLQKTIQQFSYELNTSYPLPKF 451

.

MvContig02305 DAYFRTIILNFNCRYGNEDCVNTAENLLQSYR-TTGQLPHPDIQTTVFCS 93

BGIBMGA008017 MAYHRNIILDINCRLGNQRCVTRAQELLEQFRNNPTQRLNPDLQNTVYCS 748

BGIBMGA008061 TSMARQTVLQFACNLGHTKCIQESYDKFREMR--SGKWVSPQIRRNVYMT 705

BGIBMGA008062 RTLNRFFVLSFACNIGHDGCISNAIQKFNALRT-SGTSVNPNLRRHVFCS 770

BGIBMGA008060 TILNRMQILNLACNLGHSGCISDSLQKWRQFRNNPTNLVPVNSRRYVYCV 706

BGIBMGA008059 TDLQRMYVMEFLCNVGHEECNNFGTQAFRRWST--GTFIPANMRPWVYCA 1611

BGIBMGA008063 TLTNRLQLLEFACKIGHQGCIENTVSAFKRFKD-TGVSVSPSLRPVTYCS 746

BGIBMGA008018 VSLTRGLVMDHACRANYEPCIAAAIDLFYNPNN-------NEEVPAVYCT 789

BGIBMGA001642 HLWLRGALLASGVEWGNQGITNEAVQLFDLWME-KNHTIPEIYQEAAFTA 467

BGIBMGA001641 DVVVVDSANHYSEGWGLITLAPATLSDTKTIAR---------LLAQQWFG 492

MvContig02305 GLRGGS----SDHFEFLWSRYLLTSDSSEQSILLNALGCTSNDVSRTF-- 137

BGIBMGA008017 GLRGGD----RDNFNFLWEQYLASSDSSEQNILLNALGCYMNQVIDANSP 794

BGIBMGA008061 GMREGD----SSDFEYLLNRFRQSNFANDQLEMLRGLGASKDSQLLTRYL 751

BGIBMGA008062 GLLEGG----YNEWRFLYERRKNSNNQGDEVAMLRSLGCTTNPQARQEYL 816

BGIBMGA008060 GVRQGN----SSDYNFLFERYNASQNTADMVVMLRALACTRDTNSLQHYM 752

BGIBMGA008059 GLRHGT----AEDFNFFWNRYLQEDLSSEKVVMLNVAGCTTDQASLNRFL 1657

BGIBMGA008063 GLRFGD----ASDYEFLWERMSTTNVANEARVISGVLGCSTDEGSLEKFL 792

BGIBMGA008018 MVREGD----EDVIEALKARLEVEANHYERVVILESLACSDDQNFIRNYL 835

BGIBMGA001642 GVRTHGRVAWRACWRALVDSYSAPRPTYSHRALLAALASPEDDWLFYRFA 517

BGIBMGA001641 GLVSPR------WWASQWLMEALTSLIAEKAPPFKNSALKQEEALLLDHV 536

**F)** Assembly of contig00522, contig00524, and contig 02783

00522_00524_027831 --------------------------------------YVERD--GESFM 10

BGIBMGA008059 NFLIRRLAHDSTNAALLQ-----KLILELSPAVVAKLGYLEPE--NGSYM 1563

BGIBMGA008061 TWLRNRLRHLPD-QATFD-----AYILELMETVINTYGFDAAA--NEPPT 657

BGIBMGA008062 GWLRNRFLHMPDVLAEFN-----TILYTFLEAVIADLGYDVVD--GEPLT 721

BGIBMGA008060 EWIRRRLEHLPQAHEAFT-----AYTLDLLRNVINHLGYNERA--TDSTS 656

BGIBMGA008063 NKLWN-SYLGDSALEDIE-----RLALKFIDNAITTIGYEVRA--TDDFS 697

BGIBMGA008017 N-YLDIVLTGSSVYNLFR-----EYLLTLTAPLYDEIGWEATA--NEEHV 698

BGIBMGA008018 DFLRKRLIAHVTEDDDLDPDIYMRMVRRTVGALEDEIGFNPDTSLTEPAM 746

BGIBMGA001642 SWWRELLREGAAAPALAR----------LLATLHPPIALRHQR-DADSDD 418

BGIBMGA001641 -----------------------GPLLEWLQKTIQQFSYELNTSYPLPKF 451

00522_00524_027831 DGIGRMYVMNFLCDIGDEQCTVTGKTYFDNWKD--GAFIPANMRPWVYCV 58

BGIBMGA008059 TDLQRMYVMEFLCNVGHEECNNFGTQAFRRWST--GTFIPANMRPWVYCA 1611

BGIBMGA008061 TSMARQTVLQFACNLGHTKCIQESYDKFREMR--SGKWVSPQIRRNVYMT 705

BGIBMGA008062 RTLNRFFVLSFACNIGHDGCISNAIQKFNALRT-SGTSVNPNLRRHVFCS 770

BGIBMGA008060 TILNRMQILNLACNLGHSGCISDSLQKWRQFRNNPTNLVPVNSRRYVYCV 706

BGIBMGA008063 TLTNRLQLLEFACKIGHQGCIENTVSAFKRFKD-TGVSVSPSLRPVTYCS 746

BGIBMGA008017 MAYHRNIILDINCRLGNQRCVTRAQELLEQFRNNPTQRLNPDLQNTVYCS 748

BGIBMGA008018 VSLTRGLVMDHACRANYEPCIAAAIDLFYNPNN-------NEEVPAVYCT 789

BGIBMGA001642 HLWLRGALLASGVEWGNQGITNEAVQLFDLWME-KNHTIPEIYQEAAFTA 467

BGIBMGA001641 DVVVVDSANHYSEGWGLITLAPATLSDTKTIAR---------LLAQQWFG 492

00522_00524_027831 GLREGN----ATDFDFFWNQYLAVDLASEQVVMLQAAGCTSDVESLEKFL 104

BGIBMGA008059 GLRHGT----AEDFNFFWNRYLQEDLSSEKVVMLNVAGCTTDQASLNRFL 1657

BGIBMGA008061 GMREGD----SSDFEYLLNRFRQSNFANDQLEMLRGLGASKDSQLLTRYL 751

BGIBMGA008062 GLLEGG----YNEWRFLYERRKNSNNQGDEVAMLRSLGCTTNPQARQEYL 816

BGIBMGA008060 GVRQGN----SSDYNFLFERYNASQNTADMVVMLRALACTRDTNSLQHYM 752

BGIBMGA008063 GLRFGD----ASDYEFLWERMSTTNVANEARVISGVLGCSTDEGSLEKFL 792

BGIBMGA008017 GLRGGD----RDNFNFLWEQYLASSDSSEQNILLNALGC---------YM 785

BGIBMGA008018 MVREGD----EDVIEALKARLEVEANHYERVVILESLACSDDQNFIRNYL 835

BGIBMGA001642 GVRTHGRVAWRACWRALVDSYSAPRPTYSHRALLAALASPEDDWLFYRFA 517

BGIBMGA001641 GLVSPR------WWASQWLMEALTSLIAEKAPPFKNSALKQEEALLLDHV 536

00522_00524_027831 DAIVADEDLVRPQDFTTALASAVRRNEYNTLRVFDWLKRSLPQATATLGG 154

BGIBMGA008059 DAIVSGNDDIRPQDYNAALTSAITSNEINTLRAFQWLRNNVDQATRTLGS 1707

BGIBMGA008061 QLTLT--REVRSHDKATSFNYALLGNQENANTVLQFVKNNIAAIRTAYIE 799

BGIBMGA008062 SMILS--DDVKAQDRVNALTFFYMGDRSNANVALQYLKENFEEIRQG--- 861

BGIBMGA008060 FQSMHN-DRIRIHDRTNAFSYALQGNRENLPIVLNFLYQNFAAIRTSYGG 801

BGIBMGA008063 FSVKEENSPIRTQDLTVPLTGVLS-NHSHIYIVMDSLKQNYSAWSSIKCS 841

BGIBMGA008017 NQVIDANSPVREQDRHTILVSVINSSPENMDAALEFVIENFHRIQPRVQG 835

BGIBMGA008018 EETIAAGNEYGVEERVRIFRAVAESSYENARVALSFISMRTNEIRDNYGG 885

BGIBMGA001642 FTVLST-EAQRGRDWTEWITALYTSTCR---------------------- 544

BGIBMGA001641 LPALR--------------------------------------------- 541

00522_00524_027831 VGTLLS-------------------------------------------- 160

BGIBMGA008059 VSTILN-------------------------------------------- 1713

BGIBMGA008061 DAPPTPVHTALS-------------------------------------- 811

BGIBMGA008062 VVLPAWFDNVIS-------------------------------------- 873

BGIBMGA008060 EARLVTAVNAISGF-----------LTDFAIIRENTWGYQIYNGQNVQVF 840

BGIBMGA008063 NYHICMVSKLNILNGVNQFIVFAFLKRGRGVVQDDLVGFAGANFDRVGCK 891

BGIBMGA008017 LTGTTNILN----------------------------------------- 844

BGIBMGA008018 PKKLEEILFVLG-------------------------------------- 897

BGIBMGA001642 --------------------------------------------------

BGIBMGA001641 --------------------------------------------------

00522_00524_027831 ----------YIAARLLNESD----------------------------- 171

BGIBMGA008059 ----------TIIGRLLNEEQ----------------------------- 1724

BGIBMGA008061 ----------NLAAYLDESG------------------------------ 821

BGIBMGA008062 ----------NLASYLNEEG------------------------------ 883

BGIBMGA008060 GLDTSGSLVVTLAEGLLDEGGCMITDNYYTSD-----PLTEFMLSRNTDL 885

BGIBMGA008063 HQCFRARQERTTPKRFHRENRTDKIHDLQETLRRRTIPKFKFKFKYFRMF 941

BGIBMGA008017 ----------AFARRLTTETH----------------------------- 855

BGIBMGA008018 ---------ENMANDILSED------------------------------ 908

BGIBMGA001642 --------------------------------------------------

BGIBMGA001641 --------------------------------------------------

00522_00524_027831 ----IQEFESWLNENQAALGAAYNTGINGANSARNN-LQWSEQRLPEFVK 216

BGIBMGA008059 ----INEVSNWLTANQNTLGATYSTALRAIETTRSN-LVWSQQRISEFTN 1769

BGIBMGA008061 ----LDEYETWLRSTQTNIP-QYNSALSAINSARSN-IAWGTANAEMLL- 864

BGIBMGA008062 ----LEDMESWLRANQNMIP-NFNVGLNAINSARTS-MQWGTDRAQEIL- 926

BGIBMGA008060 CGTIREKFQSWVYANQLALGTAFSTGVSVINSAISN-LEWGNAEATDIYN 934

BGIBMGA008063 FALLLFVLFSYSNANFIVEEECLNYTVYPIQYELTI-IPYIYKDNSYYHG 990

BGIBMGA008017 ----AERINQLISRHQAILTAGEQASISAIREHIAASIAWGKDNAAVVED 901

BGIBMGA008018 -------FRIWVRSQSNDLDDSQGAANRALAIVLEN-VNWIERHQDYVYD 950

BGIBMGA001642 --------------------------------------------------

BGIBMGA001641 --------------------------------------------------

00522_00524_027831 YFETGYLEDNIDEPTTPAPIDQETDATLEPEDNFTTETPTTLPDSANLAT 266

BGIBMGA008059 YFESGYVEDVIEEITEAPPTAPPTAPPTEAP------AVTPAPDSANVAA 1813

BGIBMGA008061 ---------------------------------------AAARDSATAVV 875

BGIBMGA008062 ---------------------------------------KAARGSAVTVL 937

BGIBMGA008060 --------------------------------------FLLARSSSTTVT 946

BGIBMGA008063 DITITVIANANVREIELDAKDLDIQSGSIKVLDGSTDLVNGARPYEYDKT 1040

BGIBMGA008017 WLEDNYGE------------------------------PKPEEPSAAHST 921

BGIBMGA008018 ---------------------------------------WIDENDAPTLA 961

BGIBMGA001642 --------------------------------------------------

BGIBMGA001641 --------------------------------------------------

00522_00524_027831 LSVLTLMITLAINLVN---------------------------------- 282

BGIBMGA008059 LSFITLIITLAVNLA----------------------------------- 1828

BGIBMGA008061 TSAALLAITTLFALIM---------------------------------- 891

BGIBMGA008062 PTFMLLVPTLAMLVLK---------------------------------- 953

BGIBMGA008060 SSFILMITALVVKMFH---------------------------------- 962

BGIBMGA008063 NGKLFIHLREPLKVYSQNNRQFYYIKMSFNKYIKEDSAGLFLVNYYEDDV 1090

BGIBMGA008017 TAGFIVLLSAFVAFFNIH-------------------------------- 939

BGIBMGA008018 ASLILICITIFVTLFNN--------------------------------- 978

BGIBMGA001642 --------------------------------------------------

BGIBMGA001641 --------------------------------------------------
