# Supplementary material for: Transcriptome Sequencing, and Rapid Development and Application of SNP Markers for the Legume Pod Borer Maruca vitrata (Lepidoptera: Crambidae)
Source: PLoS One. 2011 Jul 6;6(7):e21388. doi: 10.1371/journal.pone.0021388 (PMC3130784; doi:10.1371/journal.pone.0021388)
Supplement: Data S4 — Oligonucleotide primers used in Maruca vitrata PCR multiplex W1. (DOC) [file pone.0021388.s004.doc]

**Supplementary Data S4:** Oligonucleotide primers used in *Maruca vitrata* PCR multiplex W1

| Marker | A_1_ | A_2_ | F-primer | R-primer | Interrogating primer |
| --- | --- | --- | --- | --- | --- |
| Contig810_345 | T | C | ACGTTGGATGCCCTGAAGTTCCTGAAGCG | ACGTTGGATGTCATCTGTACCAGCACGTTG | ACGCTTCCTCTTGGC |
| Contig896_1927 | G | A | ACGTTGGATGGAGGTACCAACGGTGGATTC | ACGTTGGATGGAAGGCCTCAAGCTCCTTAG | AGCGAACACGAAGAA |
| Contig702_243 | C | T | ACGTTGGATGGTATACATTACAGCTGACTG | ACGTTGGATGGCGCCCAAGAAATAGCCAAC | AGCCAACAATCCTACG |
| Contig1663_801 | A | G | ACGTTGGATGCAAAAGTAACGCTTTTAAGG | ACGTTGGATGACACGACTCTAATAGTAGGC | aAGGCCTTTACACCCG |
| Contig321_1111 | C | T | ACGTTGGATGCAGATGACTCGTTATACGAC | ACGTTGGATGCGTGTTAGCGACTCTTCGAC | TGAAGGAGAGAGCGAA |
| Contig107_43 | C | T | ACGTTGGATGCACGCACCCATTCTCAAAAC | ACGTTGGATGAGTAAGAACTGCTTACCTGC | CTTACCTGCAGGTGAAT |
| Contig180_816 | A | G | ACGTTGGATGATATACATTCCGTGTACGGC | ACGTTGGATGAAGTCAGTGTTAGCAGCGAG | TCATTTAAGGACCCGAG |
| Contig_1692_472 | A | T | ACGTTGGATGAAGCTGAAGCCAACCAGGTG | ACGTTGGATGTTGGGCCGTTTCCTCCGTTT | CCTCCGTTTCCTGAATGC |
| Contig141_578 | C | A | ACGTTGGATGTGCGCATGCATTGTCGTAAG | ACGTTGGATGTTGTCGACTACAATGGCACC | agCCTGTTTGTGGTACGG |
| Contig1663_832 | C | T | ACGTTGGATGCCTAGAGCAATACAAGTTGA | ACGTTGGATGCGATGTGTCGGGTTATGTTG | CGGGTTATGTTGGAGGGC |
| Contig134_915 | T | C | ACGTTGGATGAGGTACAAGGGTAAAGATGG | ACGTTGGATGCCTTGCTTGGTCTTGGACAG | aTTGGACAGGATGGTGGT |
| Contig355_182 | G | A | ACGTTGGATGTGCCATAATCAAAGAAGAGG | ACGTTGGATGAACAGCTTGTAGGTGTCTCC | ggtTGTCTCCGTTCTGTTC |
| Contig26_1757 | C | A | ACGTTGGATGAATGCTAAGAGAGATGTTCG | ACGTTGGATGTGCCACAAACCCAAATCCTC | AAACCCAAATCCTCTAGAAG |
| Contig179_203 | G | C | ACGTTGGATGCTGGCTGCGGCAAATCCCT | ACGTTGGATGTTCTTGTGGACTTTGCCGTG | caGTGACCTAGGACGACTGG |
| Contig125_295 | A | T | ACGTTGGATGCTTGGTGGAGACCATCGAAC | ACGTTGGATGTCGAACAAGGCAGAGGTATC | gtCAGAGGTATCTGCGGCGG |
| Contig150_844 | C | T | ACGTTGGATGTAGATGTGCATGGAGACGAC | ACGTTGGATGAACGTTCAGCTGATCGTGCC | cggaCCTCAGTGCCTCTACGA |
| Contig143_291 | T | C | ACGTTGGATGCGTTATCACACCAAGGTTCG | ACGTTGGATGAAAAGCAGGGTTCAGTCCAG | ccggCCAGCAGCACGAATTTC |
| Contig141_885 | G | C | ACGTTGGATGGCATGCACTTACGAGTACAG | ACGTTGGATGTCTCACAATCTAGGACGCAC | ggggTAGGACGCACCGAGTGG |
| Contig582_620 | A | G | ACGTTGGATGCATTTTGAGCCCGCTTTGAG | ACGTTGGATGAAAATCCTATCGCGGTGCTC | cttCTCTTCAGTGAGTGTCGAG |
| Contig104_168 | T | C | ACGTTGGATGATGACGATGGTACAACCTAC | ACGTTGGATGAGCAATATCCAGGAAAATC | GAAAATCTGAACCTAAAGAAGT |
| Contig20_95 | G | T | ACGTTGGATGAAGGCCAGGAGGTCAGGTA | ACGTTGGATGACTATCAGTATGCGCTGCGT | accatTATGCGCTGCGTTTCCCA |
| Contig198_550 | C | A | ACGTTGGATGCCCAGTTCTATCAGCTTCTC | ACGTTGGATGCCAGCGAAGTTCTCCTGAAG | tctccGTTCTCCTGAAGTTCAGA |
| Contig174_560 | T | A | ACGTTGGATGTCAGGACATGAAGATGTCGG | ACGTTGGATGAGGATGCACAAAGCGAATGG | AAGCGAATGGTATGTACGAATGA |
| Contig1126_314 | G | T | ACGTTGGATGCCAGATTAACCGAAGGATGT | ACGTTGGATGGGAAACGAAATGCAATCTT | gcacTGCAATCTTAAATTACGGTT |
| Contig27_516 | G | A | ACGTTGGATGTCTAACAACGCCCAAGAATC | ACGTTGGATGGACTCTTGCTCTTCAGCTTC | tgCTCAGAGTTGTTAGAAGATTCC |
| Contig141_638 | C | T | ACGTTGGATGGTAAGCAACTTTTATTGCGGG | ACGTTGGATGTTACGACAATGCATGCGCAC | ggggcACAGACGAAGGAAAAGACG |
| Contig5_210 | C | A | ACGTTGGATGATCGTTGCTTAGGAACCCTG | ACGTTGGATGACACATCGCGCTGCGGTAAG | cataGCGCTGCGGTAAGCACAGATC |
| Contig233_190 | C | G | ACGTTGGATGTTGCTTTGATCGTAGCGTAG | ACGTTGGATGTCTTTCCCGATGATGGTGAG | gacaGATGATGGTGAGGACAAGCAA |
| Contig192_128 | C | A | ACGTTGGATGAGCGTTGCTACAACCTGAAG | ACGTTGGATGGTTCCACCGTGAACAGTTTG | cccaaCGTCAATCGAAAGATCCATAT |
| Contig180_676 | T | C | ACGTTGGATGTAGTGATGTCCCAAGCGAAC | ACGTTGGATGCCATGATAGCGTTCACGATG | cttTTGTTCCGGAGGGCTCTCACAGC |
| Contig1208_377 | A | G | ACGTTGGATGCATCAGGTCCCAAATTATCG | ACGTTGGATGGAAGCAATTGCGAACGGAAG | CAGGATATTTTATCAACTATACAGACG |
| Contig531_693 | T | C | ACGTTGGATGCCCTGAGCCATGTCAACTTG | ACGTTGGATGGCTGCTCTGAAGAATCAACG | gaaatCAAATCGGCAGACGGAGTTAGC |
| Contig702_420 | G | T | ACGTTGGATGGATGATATCTCATCTTCAAAC | ACGTTGGATGGACATCACCTTCACTACGAG | ATATAAAGAAGAAAAGATTAGAAAAGA |
| Contig107_571 | G | C | ACGTTGGATGGGACACTTAGAGTTGCATTC | ACGTTGGATGGTTACGCAGTGAAATATTTA | cccCAATATATTTTAACACGTACAAGTA |
| Contig422_331 | T | C | ACGTTGGATGGAAAAATGGACTGCTCAG | ACGTTGGATGGTCTCACTCTTACTGTACAC | TTATTTTCAAATGTACATTAAATAAAGT |

**Supplementary Data S4:** Oligonucleotide primers used in *Maruca vitrata* PCR multiplex W2

| Marker | A_1_ | A_2_ | F-primer | R-primer | Interrogating primer |
| --- | --- | --- | --- | --- | --- |
| Contig228_794 | C | T | ACGTTGGATGTCAAGAAGCAGCTGAAGACC | ACGTTGGATGTTTCTTGACGGTCTTCTCGG | ACCCTCACCAGCAAAC |
| Contig102_840 | T | C | ACGTTGGATGTCACTCGACCTGATGGTAAG | ACGTTGGATGTACTATTGTGCCTGTCGACC | GGTAAGCGGCCATGAA |
| Contig172_349 | A | G | ACGTTGGATGTATTTGTGGTAGGCACGTCC | ACGTTGGATGCATCCAGCAACAGAGGAATG | aACCACCTCCGGCCACA |
| Contig299_742 | G | A | ACGTTGGATGCATTTTGAGCCCGCTTTGAG | ACGTTGGATGAAAATCCTATCGCGGTGCTC | TAAAATTGTCGGCCCAC |
| Contig1509_742 | G | A | ACGTTGGATGTTGGTAGATGTAAGGGCGGG | ACGTTGGATGACACATGTGGCCTCATGACC | GGAGACGGTCGTAGATC |
| Contig207_963 | G | C | ACGTTGGATGCATTTAAACGACTCCGCGCA | ACGTTGGATGTTACAATTGCCGTCCGTCTC | aACTCCGCGCAAATATAC |
| Contig_1692_590 | T | C | ACGTTGGATGCATCGCTCGATTTGACCTTG | ACGTTGGATGCAAAGTGGGCCGTATCACTG | ttTAGAGGACCGGCAGGA |
| Contig57_204 | T | C | ACGTTGGATGACAGATGGTACACGATCTGC | ACGTTGGATGTCGAAGACATGTGGATGACG | cTCTCGCGGCTCTCTCCAA |
| Contig134_804 | C | T | ACGTTGGATGACAGCGGAGTCAACATCAAG | ACGTTGGATGCCATCTTTACCCTTGTACC | CAACATCAAGAACAACGAC |
| Contig167_588 | C | T | ACGTTGGATGTACCAGGGAGATGGAGCAG | ACGTTGGATGATGTAGAGTCTTGTGCGGTG | CGGCAAGACGTACAACAAC |
| Contig172_430 | G | A | ACGTTGGATGCTTTGCCACAGTTATCGGAC | ACGTTGGATGTGGATGGCAGCACTTTCTTG | cctTTATCGGACACAACCCC |
| Contig780_144 | C | T | ACGTTGGATGGGTGTGGTAGATCGTCATAC | ACGTTGGATGACTGACCAGTGGTAGGATCG | gggCGAAATGCGCTCAGTGC |
| Contig47_687 | A | G | ACGTTGGATGTATACCAACTTGGAGCAACG | ACGTTGGATGCCCAATGGAGATATATTCCAG | aTGGAGCAACGTTAGTGAAA |
| Contig163_401 | C | T | ACGTTGGATGATGTGTGGACTACCAATGCG | ACGTTGGATGGGAAACAAATGCAGCTATCC | ccCCAATGCGCTGAGACTTGC |
| Contig1478_266 | G | A | ACGTTGGATGTTTAGCTTTGTTGGCGTCCG | ACGTTGGATGACACAAAGTAGGACCCAACC | CCAGTTTTACGGCCAAAGAAC |
| Contig20_95 | G | T | ACGTTGGATGACTATCAGTATGCGCTGCGT | ACGTTGGATGAAGGCCAGGAGGTCAGGTA | ccccATGCGCTGCGTTTCCCAC |
| Contig26_1164 | C | T | ACGTTGGATGTAACAGGAGGCTGTGCAGTC | ACGTTGGATGAACTATTCCGCCAGCTCAAC | aaACCGCGGTGATGGTCTGATC |
| Contig192_262 | A | G | ACGTTGGATGGAAATCGACTTTGTCCCTTG | ACGTTGGATGTTCACCCCGAACTGACAGAT | cttgtACCGTGAAGAACGAGGA |
| Contig315_310 | G | A | ACGTTGGATGTATACGCTCACTTCCCCATC | ACGTTGGATGTCTCACCCAAGAAGTTACGG | gccaCATCAACTGTATCACCACC |
| Contig145_212 | A | G | ACGTTGGATGAGGTCACCACCATGGACAGA | ACGTTGGATGTGGTGGTGATGGACGCTCTA | tattCTAAAGTGTCCACCCGAGA |
| Contig1208_419 | G | C | ACGTTGGATGCTGATTTTTCCAATTTTGCC | ACGTTGGATGTAACGATAATTTGGGACCTG | TTCTTTTATTTTCTCCAACAACTC |
| Contig163_323 | C | T | ACGTTGGATGGTCAATGGACGCTGCAAATG | ACGTTGGATGTGCTGCATACATGCTATGTG | ATCTTACGAGTCTAGATATTGTCC |
| Contig531_777 | C | T | ACGTTGGATGCCGTTGATTCTTCAGAGCAG | ACGTTGGATGATTTCTATTGACGACGAGGG | ttcgtCATTGGTACGGTCGGAACC |
| Contig952_1113 | C | T | ACGTTGGATGAGCTGTGGGCCTCTATCATC | ACGTTGGATGTCGGGCACGTGTTACAAATC | ctcccCCCACAATGAAATCGGTTAC |
| Contig278_399 | C | A | ACGTTGGATGATGGGTCTCGGTAATAGTGC | ACGTTGGATGTGAGACCGTTTTACATAACC | gtcgCGGTAATAGTGCAGCGTGAAC |
| Contig392_253 | C | T | ACGTTGGATGAGGGCATCAAGAGGAAAATC | ACGTTGGATGTTCTCGAAGTTGCCTACGTC | cttcTGTTATTGACTGCACACACCCC |
| Contig98_663 | T | C | ACGTTGGATGTTTGCTTATTCATATCTTTC | ACGTTGGATGCTAATACATACATTCCACC | gGCTTATTCATATCTTTCAACAGTGA |
| Contig150_797 | G | T | ACGTTGGATGACATGAACTTCGGCAAGCTC | ACGTTGGATGTGGGCACGATCAGCTGAAC | ggtACTACTTCGAGAGGCCAACCCGG |
| Contig1302_604 | G | A | ACGTTGGATGAAGACCCTGCTAGTTATGAG | ACGTTGGATGGCATTAGCAGGTTCCGTTTG | gaatTGCTAGTTATGAGGATAAAGGC |
| Contig229_289 | T | C | ACGTTGGATGTCAAGGGAGTGGCTATTGAG | ACGTTGGATGACGATGAGATCAAGAAGCTG | ggatTGGCTATTGAGGTGATACAGGA |
| Contig1049_140 | G | A | ACGTTGGATGAGGTGTTCCAGGCTGAGGTG | ACGTTGGATGAGAAGTCCTGTCCGAAGATG | gggacGGCGCTGGCCGACCACGGCATC |
| Contig392_364 | G | C | ACGTTGGATGAGGAATGTCAGCTGTGATGG | ACGTTGGATGAGCACGTCAAAGTTGAAGGC | ctccGTGATGGCGACTTTCGTCTTGTCC |
| Contig586_808 | A | G | ACGTTGGATGCCCTTGCCTTTTCAAATGTG | ACGTTGGATGTACGCAGCTGACACACTCTC | acgTTTCAAATGTGAGAACAAGAAGAGA |
| Contig163_470 | G | A | ACGTTGGATGCAGGTTTCATAGAAAATTAT | ACGTTGGATGAGCTGCATTTGTTTCCTGTG | GTTAATTCAAACTTAAAAACTAGGATTAC |
| Contig1080_1486 | T | C | ACGTTGGATGAACGTTTGTGCTAGGTGTTG | ACGTTGGATGGACTTCGTTAAACTGAGTCT | ctgtTGCTAGGTGTTGATGAAGCTCCACA |
