# Supplementary material for: Transcriptome Sequencing, and Rapid Development and Application of SNP Markers for the Legume Pod Borer Maruca vitrata (Lepidoptera: Crambidae)
Source: PLoS One. 2011 Jul 6;6(7):e21388. doi: 10.1371/journal.pone.0021388 (PMC3130784; doi:10.1371/journal.pone.0021388)
Supplement: Data S5 — Exact test to determine the adherence of Maruca vitrata single nucleotide polymorphism (SNP) markers to Hardy-Weinberg Equilibrium (HWE) proportions within natural populations collected from Africa. Markov chain (for all loci) used a forecasted chain length of 1,000,000 and dememorization steps of 100,000. P-values that surpassed a significance threshold of α = 0.05 are indicated with an asterisk (*). (DOC) [file pone.0021388.s005.doc]

**Supplemental Data S5:** Exact test to determine the adherence of *Maruca vitrata* single nucleotide polymorphism (SNP) markers to Hardy-Weinberg Equilibrium (HWE) proportions within natural populations collected from Africa. Markov chain (for all loci) used a forecasted chain length of 1,000,000 and dememorization steps of 100,000. *P*-values that surpassed a significance threshold of α = 0.05 are indicated with an asterisk (*)

**A)** Sample location: Fada, Burkina Faso year = 2006, host plant = cowpea, sample size (N) =22.

Results are only shown for polymorphic loci

---------------------------------------------------------------------

Num.

gene Num. Obs. Exp.

Locus# copies alleles Het. Het P-value

---------------------------------------------------------------------

2 24 2 0.00000 0.15942 0.04351 ± 0.00020*

4 30 2 0.00000 0.12874 0.03454 ± 0.00019*

7 42 2 0.04762 0.04762 1.00000 ± 0.00000

8 42 2 0.00000 0.09292 0.02457 ± 0.00015*

11 34 2 0.17647 0.16578 1.00000 ± 0.00000

12 40 2 0.30000 0.38462 0.54322 ± 0.00051

13 40 2 0.05000 0.05000 1.00000 ± 0.00000

16 32 2 0.00000 0.12097 0.03202 ± 0.00017*

17 34 2 0.58824 0.42781 0.24055 ± 0.00040

18 42 2 0.00000 0.09292 0.02451 ± 0.00016*

24 42 2 0.00000 0.09292 0.02432 ± 0.00015*

26 42 2 0.04762 0.04762 1.00000 ± 0.00000

27 42 2 0.04762 0.04762 1.00000 ± 0.00000

29 28 2 0.14286 0.25397 0.21757 ± 0.00039

30 42 2 0.00000 0.09292 0.02421 ± 0.00016*

32 36 2 0.00000 0.10794 0.02852 ± 0.00017*

33 42 2 0.04762 0.04762 1.00000 ± 0.00000

34 42 2 0.00000 0.09292 0.02487 ± 0.00015*

39 42 2 0.28571 0.25087 1.00000 ± 0.00000

40 40 2 0.00000 0.09744 0.02552 ± 0.00016

41 38 2 0.10526 0.19346 0.15995 ± 0.00036

---------------------------------------------------------------------

Mean 37.905 2.000 0.08757 0.14267

s.d. 5.495 0.000 0.14677 0.10685

----------------------------------------------------------------------

**B)** Sample location: Farakaba, Burkina Faso. years = 2006 & 2007, host plant = cowpea

Results are only shown for polymorphic loci

-----------------------------------------------------------------

Num.

gene Num. Obs. Exp.

Locus# copies alleles Het. Het P-value

-----------------------------------------------------------------

2 26 2 0.00000 0.14769 0.04032 0.00020*

5 92 2 0.02174 0.02174 1.00000 0.00000

7 90 2 0.02222 0.02222 1.00000 0.00000

11 67 2 0.09091 0.27861 0.54809 0.00050

12 85 2 0.16667 0.19160 0.37962 0.00050

13 94 2 0.08511 0.08236 1.00000 0.00000

17 45 2 0.40000 0.29899 0.54809 0.00050

23 90 2 0.04444 0.04395 1.00000 0.00000

29 64 2 0.09375 0.14633 0.15514 0.00036

34 86 2 0.02326 0.02326 1.00000 0.00000

37 84 2 0.02381 0.02381 1.00000 0.00000

39 78 2 0.20513 0.18648 1.00000 0.00000

41 78 2 0.30769 0.29837 1.00000 0.00000

-----------------------------------------------------------------

Mean 75.308 2.000 0.11421 0.13580

s.d. 20.217 0.000 0.12359 0.10870

-----------------------------------------------------------------

**C)** Sample location: Farakaba, Burkina Faso, year = 2006, host plante = *D. oliveri*

Results are only shown for polymorphic loci

-------------------------------------------------

Num.

gene Num. Obs. Exp.

Locus# copies alleles Het. Het P-value

-----------------------------------------------------------------

2 10 2 0.20000 0.20000 1.00000 0.00000

7 30 2 0.13333 0.12874 1.00000 0.00000

11 12 2 0.16667 0.16667 1.00000 0.00000

12 28 2 0.14286 0.13757 1.00000 0.00000

13 30 2 0.06667 0.06667 1.00000 0.00000

17 18 2 1.00000 0.52941 0.01028 0.00010*

27 22 2 0.36364 0.31169 1.00000 0.00000

39 18 2 0.33333 0.29412 1.00000 0.00000

41 20 2 0.40000 0.33684 1.00000 0.00000

-----------------------------------------------------------------

Mean 20.889 2.000 0.31183 0.24130

s.d. 7.356 0.000 0.28253 0.14190

-----------------------------------------------------------------

**D)** Sample location: Kamboninse, Burkina Faso, year = 2006, host plant = cowpea.

Results are only shown for polymorphic loci

-----------------------------------------------------------------

Num.

gene Num. Obs. Exp.

Locus# copies alleles Het. Het P-value

-----------------------------------------------------------------

7 44 2 0.13636 0.13002 1.00000 0.00000

11 20 2 0.20000 0.33684 0.30564 0.00045

12 44 2 0.22727 0.33298 0.17953 0.00038

15 46 2 0.04348 0.04348 1.00000 0.00000

17 26 2 0.61538 0.44308 0.24531 0.00042

27 42 2 0.14286 0.13589 1.00000 0.00000

38 46 2 0.00000 0.08502 0.02235 0.00015*

39 42 2 0.19048 0.25087 0.33812 0.00048

41 32 2 0.12500 0.12097 1.00000 0.00000

-----------------------------------------------------------------

Mean 38.000 2.000 0.18676 0.20879

s.d. 9.592 0.000 0.17651 0.13709

-----------------------------------------------------------------

**E)** Sample location: Niger, Maradi, year = 2006, host plant = cowpea

Results are only shown for polymorphic loci

-----------------------------------------------------------------

Num.

gene Num. Obs. Exp.

Locus# copies alleles Het. Het P-value

-----------------------------------------------------------------

1 44 2 0.00000 0.16913 0.00176 0.00004*

2 8 2 0.50000 0.42857 1.00000 0.00000

4 20 2 0.10000 0.47895 0.02181 0.00014*

5 46 2 0.00000 0.16232 0.00149 0.00004*

7 44 2 0.04545 0.27378 0.00151 0.00004*

8 44 2 0.00000 0.24101 0.00024 0.00002*

9 44 2 0.04545 0.27378 0.00149 0.00004*

11 18 2 0.00000 0.36601 0.01196 0.00010*

12 42 2 0.14286 0.28455 0.06160 0.00024

13 44 2 0.13636 0.13002 1.00000 0.00000

14 44 2 0.00000 0.08879 0.02347 0.00015*

15 46 2 0.08696 0.16232 0.13173 0.00034

16 18 2 0.11111 0.11111 1.00000 0.00000

17 26 2 0.69231 0.50769 0.27468 0.00045

19 44 2 0.09091 0.08879 1.00000 0.00000

20 44 2 0.00000 0.24101 0.00025 0.00002*

22 46 2 0.13043 0.12464 1.00000 0.00000

23 44 2 0.09091 0.24101 0.02540 0.00016*

24 44 2 0.04545 0.20613 0.00862 0.00009*

26 44 2 0.00000 0.24101 0.00020 0.00001*

27 36 2 0.16667 0.15714 1.00000 0.00000

28 44 2 0.00000 0.16913 0.00183 0.00004*

29 30 2 0.06667 0.06667 1.00000 0.00000

30 46 2 0.00000 0.23188 0.00016 0.00001*

31 42 2 0.04762 0.21487 0.00904 0.00009*

32 22 2 0.09091 0.09091 1.00000 0.00000

35 30 2 0.00000 0.33103 0.00078 0.00003*

36 46 2 0.00000 0.23188 0.00017 0.00001*

37 30 2 0.00000 0.12874 0.03419 0.00017

38 44 2 0.00000 0.16913 0.00161 0.00004*

39 38 2 0.21053 0.27312 0.37214 0.00049

40 44 2 0.09091 0.08879 1.00000 0.00000

41 32 2 0.43750 0.46573 1.00000 0.00000

-----------------------------------------------------------------

Mean 37.515 2.000 0.10088 0.22545

s.d. 10.465 0.000 0.15701 0.11845

-----------------------------------------------------------------

**F)** Sample location: Nigeria, Samuru, year = 2005 & 2006, host plant = cowpea

Results are only shown for polymorphic loci

--------------------------------------------------------------------

Num.

gene Num. Obs. Exp.

Locus# copies alleles Het. Het P-value

--------------------------------------------------------------------

2 32 2 0.06250 0.17540 0.09676 0.00031

7 80 2 0.12500 0.11867 1.00000 0.00000

11 34 2 0.23529 0.47059 0.09922 0.00029

12 78 2 0.41026 0.33034 0.31436 0.00047

13 78 2 0.05128 0.05062 1.00000 0.00000

17 50 2 0.44000 0.39265 1.00000 0.00000

20 78 2 0.00000 0.05062 0.01297 0.00011*

23 82 2 0.00000 0.04818 0.01231 0.00011*

27 76 2 0.10526 0.10105 1.00000 0.00000

29 56 2 0.03571 0.22273 0.00072 0.00003*

35 74 2 0.02703 0.02703 1.00000 0.00000

36 88 2 0.00000 0.04493 0.01144 0.00011*

38 80 2 0.00000 0.04937 0.01306 0.00012*

39 68 2 0.29412 0.33187 0.60015 0.00050

41 46 2 0.56522 0.44928 0.34640 0.00047

--------------------------------------------------------------------

Mean 66.667 2.000 0.15678 0.19089

s.d. 18.247 0.000 0.18705 0.16201

--------------------------------------------------------------------
